# Quantitative analysis reveals how EGFR activation and downregulation are coupled in normal but not in cancer cells

**DOI:** 10.1038/ncomms8999

**Published:** 2015-08-12

**Authors:** Fabrizio Capuani, Alexia Conte, Elisabetta Argenzio, Luca Marchetti, Corrado Priami, Simona Polo, Pier Paolo Di Fiore, Sara Sigismund, Andrea Ciliberto

**Affiliations:** 1 IFOM, Fondazione Istituto FIRC di Oncologia Molecolare, Via Adamello 16, Milan 20139, Italy; 2 The Microsoft Research—University of Trento Centre for Computational and Systems Biology (COSBI), Piazza Manifattura 1, Rovereto (TN) 38068, Italy; 3 Dipartimento di Matematica, Università di Trento, Via Sommarive 14, Povo 38100, Italy; 4 Dipartimento di Scienze della Salute, Università degli Studi di Milano, Via di Rudinì 8, Milan 20122, Italy; 5 Istituto Europeo di Oncologia, Via Ripamonti 435, Milan 20141, Italy

## Abstract

Ubiquitination of the epidermal growth factor receptor (EGFR) that occurs when Cbl and Grb2 bind to three phosphotyrosine residues (pY1045, pY1068 and pY1086) on the receptor displays a sharp threshold effect as a function of EGF concentration. Here we use a simple modelling approach together with experiments to show that the establishment of the threshold requires both the multiplicity of binding sites and cooperative binding of Cbl and Grb2 to the EGFR. While the threshold is remarkably robust, a more sophisticated model predicted that it could be modulated as a function of EGFR levels on the cell surface. We confirmed experimentally that the system has evolved to perform optimally at physiological levels of EGFR. As a consequence, this system displays an intrinsic weakness that causes—at the supraphysiological levels of receptor and/or ligand associated with cancer—uncoupling of the mechanisms leading to signalling through phosphorylation and attenuation through ubiquitination.

## Abstract (web summary)

Cells respond to increasing concentrations of EGF by altering the balance between EGFR phosphorylation and ubiquitination. Here the authors show that the establishment of an EGFR signaling threshold requires both a multiplicity of binding sites and cooperative binding of Cbl and Grb2 to the EGFR.

## Graphical abstract

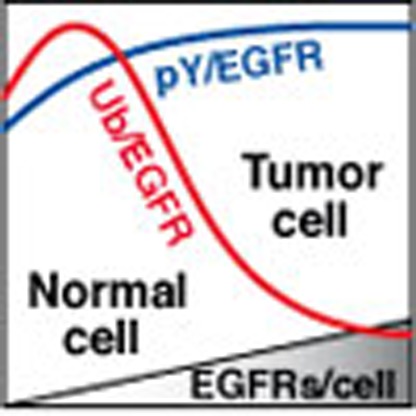

Following engagement by its cognate ligand(s), the epidermal growth factor (EGF) receptor (EGFR) forms dimers capable of autophosphorylation and of phosphorylating other proteins^1^,^2^. After EGFR dimerization the tyrosine kinase domain of one EGFR moiety phosphorylates several Tyr residues in the partner moiety^3^. The extracellular domain of EGFR can adopt two conformations, closed and extended^4^, the latter one being dimerization-competent^5^,^6^. EGF binding stabilizes the extended form, thus favouring dimer formation, and allows the EGFR kinase domain to reach its Tyr substrates^7^,^8^,^9^,^10^,^11^. The kinase activity is contrasted by phosphatase acting at the membrane already at the very early stages of EGFR signalling^12^,^13^,^14^,^15^.

Molecules harbouring modules binding phosphotyrosines of EGFR (pY) are recruited to the plasma membrane (PM) and activate signalling pathways leading to context-dependent biological outputs^1^,^2^. One such molecule is the ubiquitin ligase Cbl, which binds to the EGFR via a pY-mediated mechanism and ubiquitinates the receptor^16^. EGFR ubiquitination is critical for receptor trafficking through endosomal/lysosomal compartments^17^,^18^,^19^, and for the internalization step at the PM^20^,^21^,^22^.

We have recently shown that the dose–response curves for EGFR Tyr phosphorylation and ubiquitination display different degrees of sigmoidicity, best approximated by Hill functions with Hill coefficients (*n*_H_) of 1 and 3, respectively (see Fig. 1a for a schematic of the interplay between phosphorylation and ubiquitination)^21^. In the case of ubiquitination, the dose–response curve has a threshold between 1 and 10 ng ml^−1^ of EGF in HeLa cells. This ubiquitination threshold is an important biological feature, as it controls the modality of EGFR internalization at the PM, and thereby enables cells to translate quantitative inputs (EGF concentrations) into qualitatively different internalization mechanisms^21^,^23^. At low EGF concentrations (≤1 ng ml^−1^), the EGFR is scarcely ubiquitinated and internalized primarily through clathrin-mediated endocytosis (CME). At high EGF concentrations (>10 ng ml^−1^), a significant fraction of the EGFR is endocytosed through a clathrin-independent pathway (non-clathrin endocytosis, NCE), as the receptor becomes ubiquitinated. CME and NCE are associated predominantly, although not exclusively with (i) EGFR recycling to the PM and maintenance of signalling, in the case of CME, and (ii) EGFR degradation and signal attenuation, in the case of NCE^21^. Thus, the ubiquitination threshold controls receptor fate and the balance between maintenance versus attenuation of EGFR signalling.

A clue towards understanding the ubiquitination threshold is the fact that phosphorylation and ubiquitination are causally related. To ubiquitinate the EGFR, Cbl needs to bind to the receptor. Binding occurs either directly, via a pY-binding module contained in Cbl that binds to pY1045 of the EGFR, or indirectly, through binding of a Grb2:Cbl complex to either pY1068 or pY1086, via the SH2 domain of Grb2 (refs 16, 24, 25, 26). Crucially, Y1045, Y1068 and Y1086 are not only necessary but also sufficient for EGFR ubiquitination, as an EGFR mutant that carries only these three Tyr residues (EGFR-3Y+), and none of the other phosphorylatable Tyr residues (see Fig. 1b for a schematic of the EGFR phosphorylation sites), shows a ubiquitination dose–response curve that is indistinguishable from EGFR wild-type (EGFR-WT)^21^. Conversely, ubiquitination of an EGFR mutant (EGFR-Y1045+) that contains only the phosphorylatable Tyr residue responsible for direct Cbl binding (Y1045) does not display a threshold and is significantly reduced compared with EGFR-WT (∼80% reduction)^21^.

Here we account for all these data providing a system-level description of the mechanisms that contribute to the creation of the threshold and that, consequently, determine the cellular response (maintenance versus attenuation of signalling) to varying EGF concentrations. We took advantage of the many existing models of EGFR activation and signalling^12^,^27^,^28^,^29^,^30^,^31^,^32^,^33^,^34^, while also taking into account the ubiquitination component of the system that has so far received scarce attention^35^.

## Results

### Multisite Phoshorylation Model (MPM)

We initially developed a simple model to account for EGFR phosphorylation at 2 min of EGF stimulation, when both phosphorylation and ubiquitination take place predominantly at the PM^21^.

**Figure 1:**
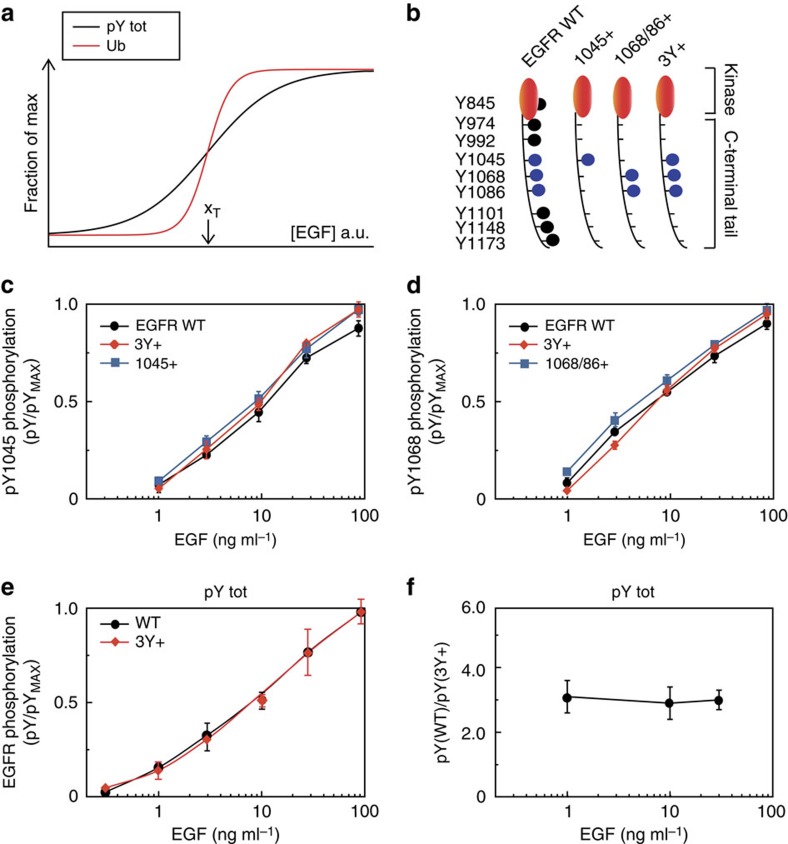
EGFR phosphorylation dose–response curves. (**a**) Schematic representation of EGFR ubiquitination (Ub) and total EGFR Tyr phosphorylation (pY tot). *x*_T_ represents the half-maximal EGF concentration for EGFR ubiquitination (that is, the ubiquitination threshold). EGF concentration is expressed in a.u. (**b**) Schematic representation of Tyr phosphorylation sites in the cytoplasmic tail of EGFR-WT (left) and of the add-back mutants (1045+, 1068/86+, 3Y+), some of which were used in **c**–**f**. The intracellular domain including the kinase domain (red) and C-terminal tail (black line) of the EGFR are shown. The position of the nine phosphorylatable Tyr residues is shown. Tyr residues involved in Cbl/Grb2 binding and subsequent EGFR ubiquitination are indicated in blue, while the other Tyr residues are depicted in black. Phosphorylation of Y1045 (**c**) or Y1068 (**d**) in EGFR-WT or the indicated add-back mutants. Experimental points are taken from ref. 21. Phosphorylation is plotted, for each condition, as normalized to the maximum pY value obtained in that condition (pY/pY_MAX_). (**e**) Experimentally determined dose–response phosphorylation curves for EGFR-WT and EGFR-3Y+. Experimental points are taken from ref. 21. EGFR phosphorylation is expressed, for each condition, as normalized to the maximum value obtained in that condition (pY/pY_MAX_). (**f**) Experimental ratio of total Tyr phosphorylation (pY tot) of EGFR-WT and EGFR-3Y+ after 2 min of stimulation with the indicated concentrations of EGF. Data are derived from densitometry analysis of IBs from three independent experiments±s.d. (see Supplementary Fig. 1 for representative IB). In (**c**–**f**) EGFR-WT and mutants were expressed in NR6 fibroblasts, which are devoid of endogenous EGFR^21^.

We have previously shown that the dose–response curves of phosphorylation of the individual Tyr residues^21^ follow the same hyperbolic behaviour as that of the global EGFR Tyr phosphorylation (*n*_H_≈1) (ref. 21). Moreover, at 2 min of EGF stimulation, the individual Tyr residues are phosphorylated independently of each other, since their phosphorylation dose–response curves do not change in the presence (EGFR-WT) or absence (Tyr mutants, Fig. 1b) of other phosphorylation sites (Fig. 1c,d, experimental points are taken from ref. 21). Consistently, we observed that the normalized dose–response curves of EGFR-WT and EGFR-3Y+ phosphorylation were indistinguishable, both in terms of half-maximal dose (pY_0.5_) and steepness *n*_H_ (Fig. 1e, experimental points are taken from ref. 21). Moreover, in absolute terms, Tyr phosphorylation of EGFR-WT (which carries nine phosphorylatable Tyr) was approximately threefold greater than that of EGFR-3Y+ (with three phosphorylatable Tyr; Fig. 1f and Supplementary Fig. 1). Phosphatases also contribute to the net EGFR phosphorylation, as they are known to be active already at the early stages of EGFR signalling^12^,^13^,^14^,^15^,^36^.

**Figure 2:**
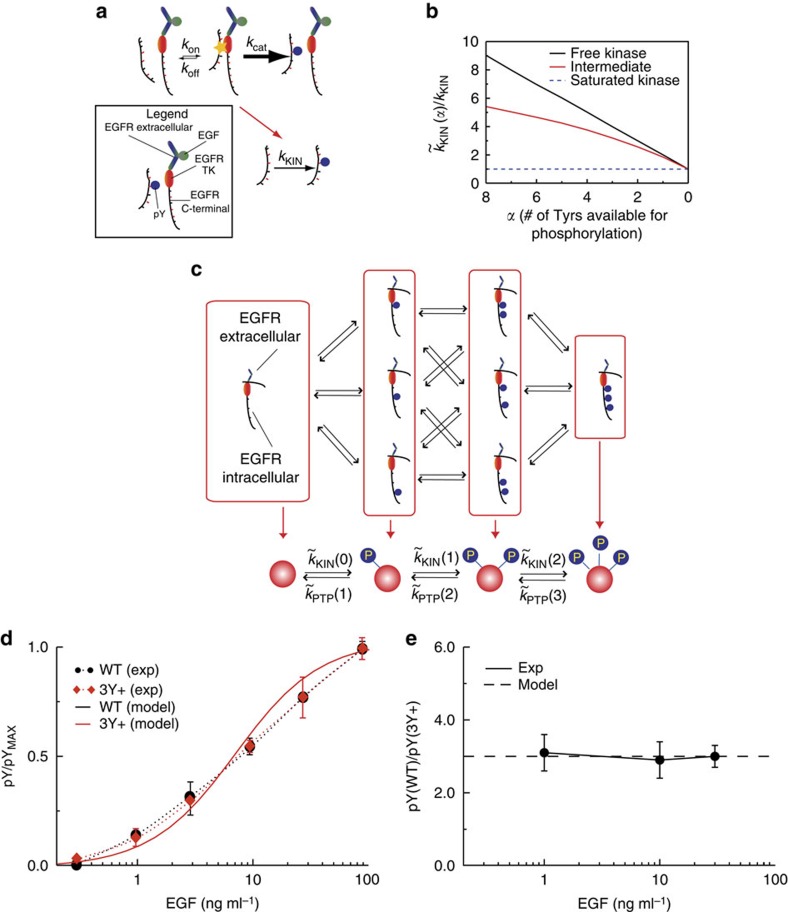
Modelling EGFR phosphorylation: the free-kinase regime accounts for experimental phosphorylation curves. (**a**) Schematic representation of the enzymatic reactions that lead to EGFR phosphorylation in an active dimer. Top, Michaelis–Menten reaction whereby the tyrosine kinase (TK) domain of an EGFR binds reversibly to the C-terminal tail of a partner EGFR with the binding/unbinding rate constants *k*_on_ and *k*_off_, respectively. After adding one phosphate group, the TK dissociates from the substrate with rate constant *k*_cat_. Bottom, the simplified reaction scheme for Tyr phosphorylation is characterized by *k*_KIN_. (**b**) Two limiting regimes (free and saturated) can be identified for the reaction catalysed by kinases, depending on the stability of the complex between the catalytic subunit of EGFR and its Tyr substrate. Curves represent the average of 10^5^ runs of the stochastic Gillespie algorithm^62^ applied to the standard Michaelis–Menten reaction scheme depicted in **a** (see Supplementary Note 1 for details). (**c**) Wiring diagram of the MPM. Top, phosphorylation of individual Tyr residues (blue circles) occurs independently of the phosphorylation of the other Tyr residues. This results in a branched wiring diagram, in which each phosphorylation event occurs with the same probability. Bottom, EGFR molecules (red circles) with the same total number of phosphoryl groups (blue circles) are grouped together to generate a linear chain of increasingly phosphorylated EGFRs. Only the three Tyr residues relevant for EGFR ubiquitination are shown (that is, Y1045, Y1068, Y1086). 
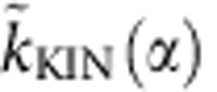
 and 
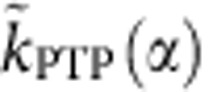
 are the rates of addition and subtraction of one phosphoryl group from an EGFR molecule that carries *α*-phosphorylated Tyr residues. (**d**) A comparison of EGFR-WT and -3Y+ phosphorylation computed by the MPM in the free-kinase/free-phosphatase regime as a function of EGF (model, solid lines) or determined experimentally (exp, dashed lines: data taken from Fig. 1e). EGFR phosphorylation was normalized to the maximum pY value (pY/pYmax). (**e**) A comparison of the ratio of total pY of EGFR-WT and EGFR-3Y+, as a function of EGF concentration (at 2 min), computed by the MPM (dashed line) or determined experimentally (solid line, data taken from Fig. 1f, see also Supplementary Fig. 1).

These data suggest a simple model for EGFR early phosphorylation (the Multisite Phosphorylation Model or MPM). In terms of elementary reactions, the individual Tyr residues are phosphorylated independently of each other when they do not compete for the kinase domain: this can occur if the kinase:Tyr complex is unstable (Fig. 2a). Under this scenario, the rate-limiting step is the kinase:Tyr binding and the kinase is expected to be mostly free (free-enzyme). The same applies for the phosphatases, whose activity at 2 min after EGF stimulation has been recently measured^12^ and implemented in our models.

Simulations of a receptor composed of nine identical Tyr residues confirm that if kinases (or phosphatases, replacing Tyr with phosphorylated Tyr) work under the free-enzyme regime, each Tyr contributes in the same way to the rate of EGFR phosphorylation. This is true regardless of the number of available sites. Accordingly, the rate of EGFR phosphorylation decreases linearly with the number of Tyr residues available for phosphorylation (Fig. 2b, black solid line). In an alternative regime, in which the kinase is mostly bound to the substrate (saturated regime), the phosphorylation rate of EGFR does not change with the number of non-phosphorylated Tyr residues (Fig. 2c, blue dashed line). In this case, nine phosphorylatable Tyr residues are phosphorylated at the same rate as only one, implying that the phosphorylation rate per Tyr residue decreases with the number of available sites. We thus chose for our model the free-enzyme regime for both kinases and phosphatases.

As a result, in the MPM model the rate constant of EGFR phosphorylation increases with the number of Tyr residues (Supplementary Note 1). In terms of enzyme kinetics, we can attribute only one phosphorylation rate constant to each Tyr residue independently of the phosphorylation state of the receptor (Fig. 2c, upper panel). Importantly, these results allow monitoring of the phosphorylation state of any individual Tyr residue while disregarding the others.

To further reduce the number of variables, we introduced a simplification in the MPM by using the same rate constants for every Tyr (*k*_KIN_ and *k*_PTP_ for the reactions catalysed by kinases and phosphatases, respectively). This allowed us to group together receptors with an equal number of pYs (that is, unphosphorylated EGFR (R_0_), 1pY-EGFR (R_1_), 2pY-EGFR (R_2_) and so on) regardless of the specific identity of the pY (Fig. 2c, lower panel). This simplification is justified by the observation that different pY sites have similar phosphorylation kinetics in the first 2 min of EGF simulation^37^. Moreover, we showed that the MPM behaviour does not differ qualitatively if we differentiate the phosphorylation rates for Y1068/86 and Y1045 (Supplementary Fig. 2). In this scheme, the phosphorylation state of EGFR is assigned on a probabilistic basis.

To compare the MPM with experimental data, we needed to define the relationship between EGFR phosphorylation and EGF concentration. To keep the model simple, we initially disregarded the numerous reactions that contribute to EGFR activation that will be included in a more complex model, the Early Activation Model (EAM; see below). Instead, we used a phenomenological law to link EGFR phosphorylation and EGF concentration. More precisely, we used a Hill function, whereby 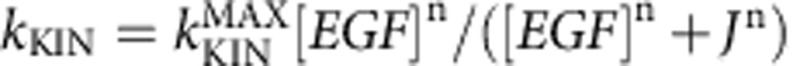, and fitted the experimental dose–response curves of EGFR-WT and the EGFR-3Y+ mutant to identify n and *J* (Supplementary Note 1).

After fitting, the MPM reproduced the dose–response curve of EGFR phosphorylation. Since we used the free regime for EGFR phosphorylation, the result is independent of the number of Tyr residues when the curves are normalized to their maximal value (Fig. 2d). Moreover, the model correctly attributed a threefold increase to the total Tyr phosphorylation of EGFR-WT compared to the total Tyr phosphorylation of the EGFR-3Y+ mutant, for every value of EGF (Fig. 2e and Supplementary Fig. 1).

In conclusion, we have developed a model, the MPM, which faithfully reproduces the distribution of EGFR-phosphorylated species, as a function of EGF concentration, at 2 min of stimulation.

### Modelling EGFR ubiquitination

We then added to the MPM the processes leading to EGFR ubiquitination. Structure–function studies with EGFR phosphomutants showed that Cbl binding is necessary and sufficient for EGFR ubiquitination and that the EGF dose-dependency of the Cbl:EGFR association *in vivo* displays a threshold-like profile, very similar to EGFR ubiquitination^21^. To describe the dynamics of EGFR ubiquitination, we therefore restricted our analysis to the dynamics of the Cbl:EGFR interaction and assumed that ubiquitination is simply proportional to the amount of Cbl-bound EGFR (Equation 15 in Supplementary Note 1).

The experimental analysis of Cbl binding to the EGFR suggested that the interaction between EGFR and Cbl is cooperative with the binding of Grb2 to the receptor^21^. Indeed, if the binding of Cbl (in complex with Grb2) to EGFR were to take place with the same affinity (*k*_on_/*k*_off_) regardless of the binding of Grb2, then EGFR-WT and EGFR-Y1045+ should bind a similar amount of Cbl. However, we have previously established that binding of Cbl to the EGFR, as well as EGFR ubiquitination, are strongly reduced in the EGFR-Y1045+ mutant, and absent or negligible in EGFR-Y1068/86+ mutant^21^. In contrast, Cbl binding and EGFR ubiquitination are indistinguishable in EGFR-WT and in the EGFR-3Y+ mutant^21^.

**Figure 3:**
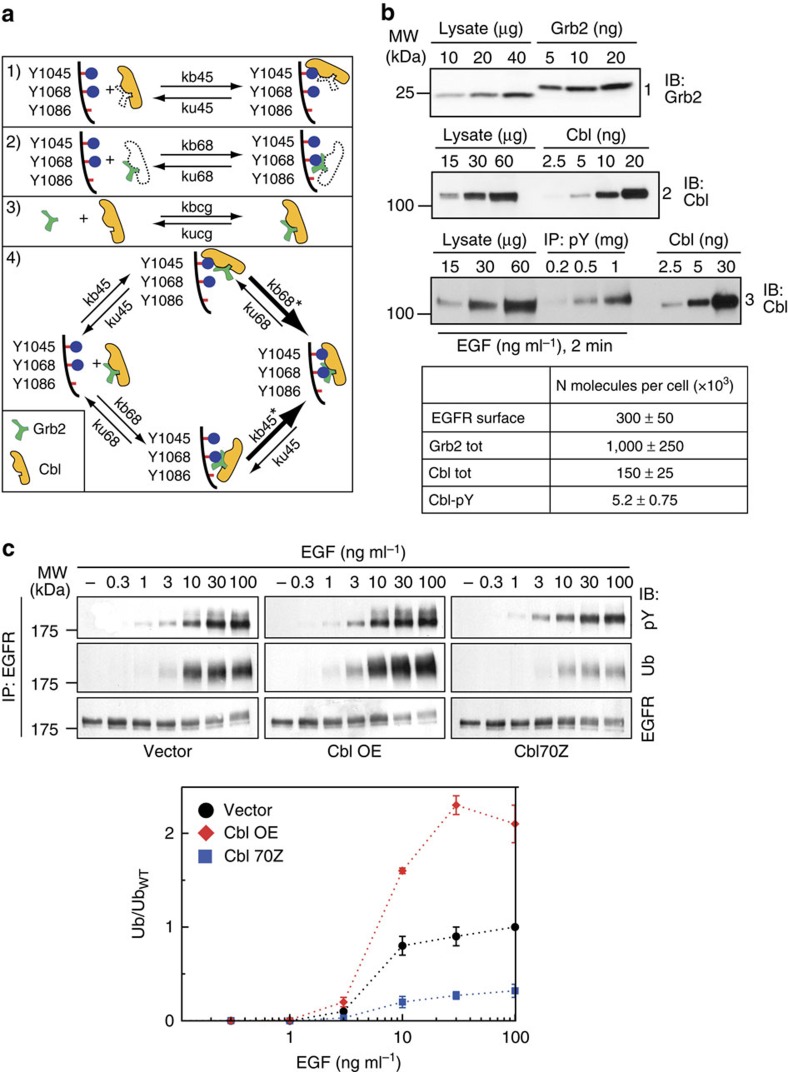
Cbl is limiting for EGFR ubiquitination. (**a**) Reactions involved in EGFR ubiquitination. (1) Cbl binds to pY1045 irrespective of whether it is free or in a complex with Grb2 (Grb2 is thus represented with a dotted line); (2) Grb2 binds to pY1068 (or pY1086, not represented in the scheme) irrespective of whether it is free or in a complex with Cbl (represented with a dotted line); (3) Cbl and Grb2 bind to each other; (4) if the Cbl/Grb2 complex binds to EGFR via reaction (1), the binding described by (2) occurs via a first-order reaction (Supplementary Note 1, Eq. (8)). Similarly, if the initial binding takes place via reaction (2), the ensuing reaction (1) will be first order. Reaction (3) can also become first order, if it is preceded by reaction (1) for Cbl only and by reaction (2) for Grb2 only (not shown). According to the cooperative hypothesis, first-order reactions are favoured (thick arrows). (**b**) Quantitation of Grb2 and Cbl molecules in HeLa cells. Panels 1, 2, 3: increasing amounts of HeLa cell lysate were subjected to IB and IP as indicated and compared with increasing amounts of *in vitro* purified Grb2 (1) or Cbl (2, 3) proteins, as described in Methods. Bottom: table indicating the amount of critical players involved in the EGFR ubiquitination reaction in HeLa cells. The number of surface EGFR molecules was measured by ^125^I-EGF saturation binding (see Methods). Data are expressed as number of surface EGFRs per cell. Average results, calculated from at least three independent experiments±s.e.m., are shown. (**c**) Top, HeLa cells, transfected with empty vector (Vector), WT Cbl (overexpression, OE) or Cbl70Z mutant, were treated with EGF at the indicated concentrations for 2 min. Lysates were IP and IB as shown (see also Supplementary Fig. 3a). Bottom, quantitation of the effect of Cbl OE or Cbl70Z expression on EGFR ubiquitination by densitometry analysis of IBs, as shown in the upper panel, from three independent experiments. EGFR ubiquitination is expressed, for each condition, as normalized to the maximum value obtained in the empty vector control (Ub/Ub_WT_).

A plausible explanation for cooperativity is that when Cbl and Grb2 are simultaneously bound to EGFR (via pY1045 and pY1068/pY1086, respectively), they are in a state of enforced proximity, which increases the likelihood of the three species (EGFR, Cbl and Grb2) of binding to each other^38^. In our model, we translated the presence of cooperativity as follows: Cbl and Grb2 can form a complex that binds to the corresponding pYs on EGFR; they can also bind EGFR individually. However, the Cbl and Grb2 binding rates (to each other and to EGFR) are increased in the multimeric complex (Fig. 3a). Assuming that enforced proximity drives cooperativity, we postulated that the binding affinity of EGFR and Cbl increases proportionally with the local increase in the concentrations of Grb2 and Cbl on the receptor (Supplementary Note 1).

We then determined experimentally the total amounts of Cbl, Grb2 and EGFR in HeLa cells. EGFR surface levels, measured by saturation binding, were calculated to be ∼300,000 molecules per cell (Fig. 3b). We also estimated that Grb2 is present at ∼1,000,000 molecules per cell (Fig. 3b). Importantly, we have previously shown that Grb2 fractions as a single monomeric species in sizing columns^21^, indicating that the majority of Grb2 molecules are either free or form very unstable complexes. Therefore, in our model, we assumed that all measured Grb2 is available for binding to EGFR and/or to Cbl.

As for Cbl, it is expressed in HeLa cells at ∼150,000 molecules per cell (Fig. 3b). However, we have previously shown, using size exclusion chromatography, that the majority of Cbl is engaged in stable complexes^21^, compatible with the notion that Cbl binds to >100 different proteins^39^. This result strongly suggests that only a minor fraction of Cbl (in the free form or engaged in Cbl:Grb2 complexes) is available for direct binding with EGFR. In the model, we used phosphorylated Cbl (Cbl-pY) as a proxy for the maximal amount of Cbl available for binding to the EGFR, because (i) Cbl is phosphorylated on binding to the EGFR and (ii) only phosphorylated Cbl is competent for EGFR ubiquitination^16^,^40^,^41^. The amount of Cbl-pY was experimentally determined as ∼5,000 molecules per cell, after stimulation with EGF (100 ng ml^−1^) for 2 min (Fig. 3b).

The above results indicate that Cbl, compared with EGFR and Grb2, is rate limiting in the EGFR ubiquitination reaction. Accordingly, its upward or downward modulation—overexpression and the expression of the dominant-negative Cbl70Z mutant, respectively—produces congruent changes in the levels of EGFR ubiquitination (Fig. 3c). We concluded that Cbl is rate-limiting in the EGFR ubiquitination process.

### MPM-B: a model of EGFR ubiquitination

Next, we used the MPM as an input for Cbl and Grb2 binding to obtain a model of EGFR ubiquitination, MPM-B (MPM plus Binding, see Supplementary Note 1). The concentrations of Cbl and Grb2 were set according to the measurements described above. The binding rates for Cbl and Grb2, for which we do not have experimental values, were identified by fitting: (i) the ubiquitination dose–response curves for the 3Y+ and 1045+ mutants and (ii) the experiments of Cbl modulation described in the previous section (Fig. 3c).

**Figure 4:**
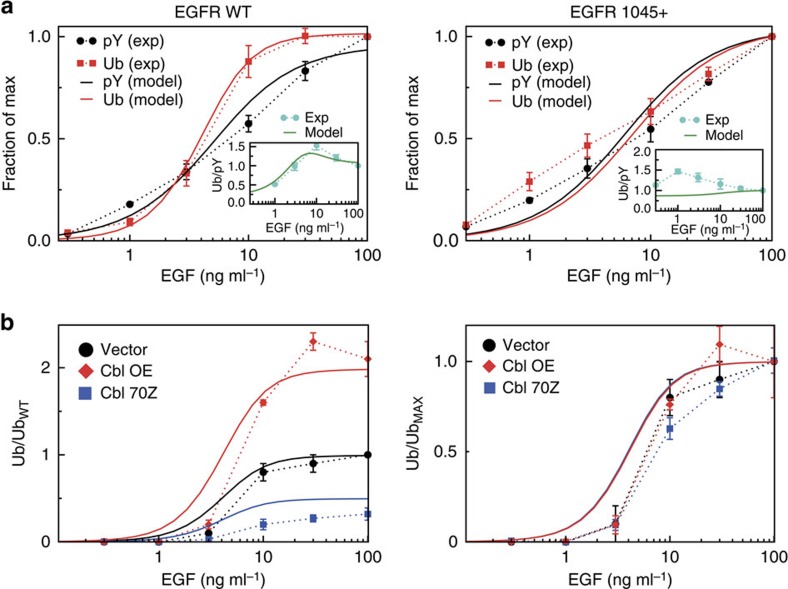
The MPM-B reproduces the EGFR phosphorylation and ubiquitination dose–response curves. (**a**) Comparison of experimental (dashed lines) and modelled (solid lines) phosphorylation (pY) and ubiquitination (Ub) dose–response curves for EGFR-WT (left) and EGFR-Y1045+ (right). Note that modelled curves are for EGFR-3Y+, since it behaves as EGFR-WT when normalized to the max (Fig. 1e). Experimental data are expressed for each condition as normalized to the maximum value for that condition; simulations are normalized to optimized maxima (see Supplementary Table 1). Inset shows the ratio of ubiquitination to phosphorylation as a function of EGF concentration for experimental and modelled data. Simulations were performed with MPM-B in the left panel, and a modified version of MPM-B where we set to zero all reactions leading to phosphorylation of site Y1068, in the right panel. In both panels, data were obtained from densitometry analysis of IBs shown in Supplementary Fig. 1 and ref. 21. Average results, calculated from at least three independent experiments, are shown. Error bars in the plots represent the s.e.m. (**b**) Comparison of experimental (dashed lines) and modelled (solid lines) EGFR ubiquitination dose–response curves under conditions of Cbl modulation. Experimental data were taken from Fig. 3c. Cbl OE and downregulation (Cbl 70Z) were modelled as a twofold increase and decrease in the MPM-B, respectively. Data are expressed, for each condition, as normalized to the maximum value in the empty vector control (Ub/Ub_WT_, left) or to their own maximum (Ub/Ub_MAX_, right). Error bars in the plots represent the s.e.m. calculated form at least three independent experiments.

We computed the model assuming that all species are at steady state in the absence of EGF; at time zero we introduced EGF to the system, and we computed the values of each species after 2 min. MPM-B reproduced, in quantitative detail, the increase in steepness of the ubiquitination curve versus the phosphorylation curve for EGFR-WT, as shown by the normalized curves, as well as by their ratio (Fig. 4a left and inset, respectively). The model also reproduced the decrease in steepness of the ubiquitination curve of EGFR-Y1045+ (Fig. 4a right and inset), albeit less precisely than the WT. Finally, MPM-B reproduced the dose–response curve for ubiquitination under all conditions of Cbl expression (Fig. 4b, left). In this case, the model suggested that the sevenfold increase in Cbl expression, obtained experimentally (Supplementary Fig. 3a), does not lead to a sevenfold increase in Cbl available for EGFR binding. Rather, the model suggests that approximately only 1/3 of the overexpressed molecules are capable of receptor binding.

Interestingly, the model also showed that modulation of Cbl levels (within a limit), despite affecting the amount of EGFR ubiquitination (Supplementary Fig. 3b,c), does not affect the ubiquitination threshold, as experimentally observed (Fig. 4b, right). This lack of effect on the threshold is not immediately obvious, given the sizable changes in total EGFR ubiquitination on Cbl modulation (Fig. 4b, left). Importantly, this condition is verified by the model only if the number of Cbl molecules available for EGFR binding does not exceed ≈5,000 Cbl molecules per cell in basal conditions (Supplementary Fig. 3b). Strikingly, this number is in agreement with the maximal number of active Cbl (Cbl-pY) molecules per cell that we estimated experimentally (Fig. 3b).

Finally, we performed a robustness analysis and observed that the model is robust to changes in parameter values with regards to both the position of the threshold and the shape of the ubiquitination and phosphorylation dose–response curves (Supplementary Fig. 4a,b). In conclusion, MPM-B robustly reproduces the behaviour of the ubiquitination threshold.

### Cooperativity helps establishing the ubiquitination threshold

**Figure 5:**
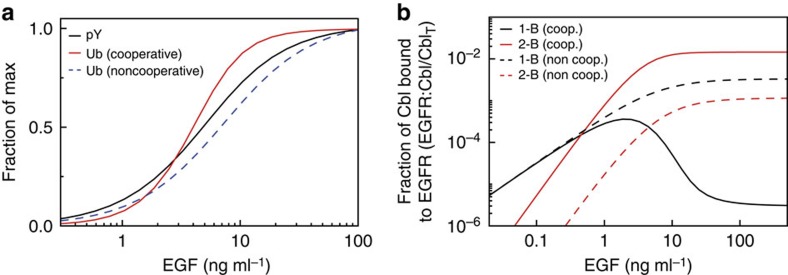
Cooperativity is required to generate the EGFR ubiquitination threshold. (**a**) Effect of simulations with a cooperative versus noncooperative MPM-B model on the EGFR-3Y+ ubiquitination threshold. In the cooperative model, the presence of enforced proximity of Cbl, Grb2 and EGFR (cooperative, red line) increases the steepness of ubiquitination as compared with phosphorylation (black solid line). In the noncooperative regime, phosphorylation and ubiquitination share the same steepness. Simulations of the noncooperative model (blue dashed line) were performed with MPM-B, except that the parameters kb45^*^, kb68^*^ and kbcg^*^ (see Fig. 3a for symbols' definitions) were reduced by setting fLOC=1, as explained in Supplementary Note 2, such that the conditions kb45*>>kb45 × [Cbl], kb68*>>kb68 × [Grb2] and kbcg*>>kbcg × [Grb2] are no longer verified. (**b**) The distribution of singly (1-B, black lines) and doubly (2-B, red lines) bound EGFR-Cbl complexes changes in the presence (cooperative, solid lines) or absence (noncooperative, dashed lines) of an enforced-proximity mechanism. Simulations were performed as in **a**.

We used the MPM-B to investigate *in silico* the potential role of cooperativity between Cbl, Grb2 and EGFR in the establishment of the EGFR ubiquitination threshold. Given that a probabilistic model of phosphorylation underlies the MPM and the MPM-B, it is not surprising that the presence of both Cbl-binding sites (pY1045 and pY1068/pY1086) is necessary for the threshold. Indeed, ubiquitination of EGFR-Y1045+, which requires only one pY to be ubiquitinated, does not display a threshold. Interestingly, if we simulate an alternative, noncooperative model (Eq. (9) in Supplementary Note 1), the threshold effect is lost also in EGFR-3Y+ (Fig. 5a). This analysis therefore suggests that the probabilistic hypothesis is necessary, but not sufficient to account for the threshold, which requires also the cooperativity mechanism depicted in Fig. 3a.

The relevance of cooperativity became evident when we compared simulations of cooperative and noncooperative models, in terms of how Cbl is bound to the EGFR: through pY1045 only (singly-bound) or through pY1045 and pY1068/pY1086 via Grb2 (doubly-bound). Only in the cooperative model, the singly bound species is almost completely converted into doubly bound Cbl when EGF concentration increases above the threshold (Fig. 5b). We concluded that the cooperative mechanism is required for the formation of the ubiquitination threshold.

### Early Activation Model (EAM)

We employed the MPM-B to understand how the EGFR ubiquitination threshold can be modulated. To identify the parameters that can alter the position of the threshold, we divided or multiplied by one order of magnitude all the parameters of the model, and derived, for the EGFR ubiquitination threshold *x*_T_, a normalized ‘sensitivity parameter’ for large perturbations of each parameter *k*_j_, 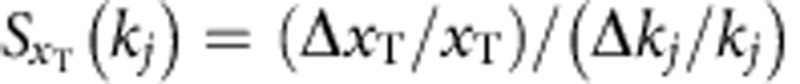. When 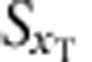 is greater than 1, the system responds with a change that is larger than the variation of the parameter. In such cases, the parameter is thus identified as a good candidate for experimental verification. The analysis identified only one parameter, the EGFR phosphorylation rate *k*_KIN_ (Supplementary Fig. 5), which can be hardly modulated experimentally.

**Figure 6:**
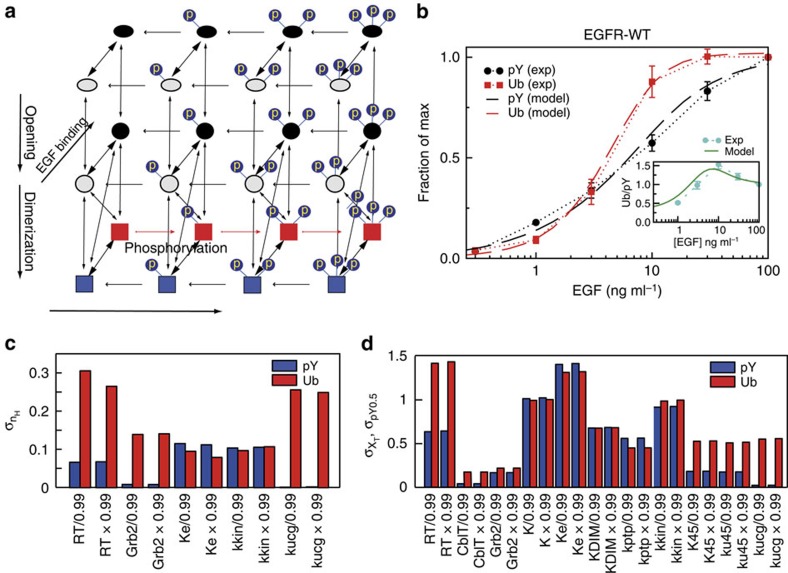
The EAM reproduces the EGFR phosphorylation and ubiquitination curves. (**a**) Wiring diagram of the EAM for an EGFR with three phosphorylatable Tyr. To reduce the complexity of the model, we did not introduce dimers explicitly; however, with a change of variables, we followed them as individual EGFR molecules that can either be monomeric or dimeric. Thus, EGFRs in a dimer can either be competent for phosphorylation and, therefore, activation or not, depending on whether the partner EGFR is bound to EGF. Each chemical species represents a single moiety of EGFR that can be either a monomer (circles and ovals) or one of the two members of a dimer (squares). EGFR moieties are characterized by different attributes: (i) presence (black) or absence (grey) of ligand; (ii) closed (ovals) or extended (circles) conformation; (iii) number of pYs (circled P). EGFR moieties in dimers may be inactive (can only be dephosphorylated, blue) or active (can be either phosphorylated or dephosphorylated, red). Opening/closing and phosphorylation/dephosphorylation follow first-order kinetics, while EGF binding (EGF is not explicitly shown) and the transition from monomer to dimer are second-order reactions. The kinetics of this latter reaction is proportional to the concentration of all species taking part in dimerization. (**b**) Comparison of experimental (dashed lines) and modelled (solid lines) phosphorylation and ubiquitination dose–response curves for EGFR-WT. Note that modelled curves are for EGFR-3Y+, since it behaves as EGFR-WT when normalized to the max (Fig. 1e). Inset shows the ratio of ubiquitination to phosphorylation as a function of EGF concentration for experimental and modelled data. Experimental data are the same as those shown in Fig. 4a left, and have been normalized for the maximum value for that condition. Simulations are normalized to optimized maxima (see Supplementary Table 1). (**c**,**d**) Sensitivity analysis. We varied each parameter by 1% and computed the sensitivity coefficient *σ* for the Hill coefficient *n*_H_ (**c**) and for both the phosphorylation half-maximum level pY_0.5_ and the ubiquitination threshold *x*_T_ (**d**), for EGFR phosphorylation (blue) and ubiquitination (red). See Supplementary Note 3.

To identify more possible targets in the model we replaced the Hill function used to couple EGF levels and EGFR activity with the molecular details of EGFR activation. The EAM, unlike MPM and MPM-B, also includes EGFR opening and closing, EGF binding to EGFR and receptor dimerization (Supplementary Fig. 6). To address these additional reactions, we started from models proposed previously^34^,^42^. As for the relationship between EGFR activation and phosphorylation, based on structural studies of the EGFR^5^,^6^,^7^,^9^,^10^,^11^, we assumed that one EGFR moiety can phosphorylate its partner only when it is in a dimer and bound to EGF. Obviously, EAM (Fig. 6a and Supplementary Note 1) carries more parameters than MPM-B; for many of these, we can define a reasonable range of values, owing to the vast amount of experimental and modelling data available in literature (Supplementary Table 1, Supplementary Table 2 and Supplementary Note 2).

When we fitted the dose–response curves for EGFR phosphorylation and ubiquitination with the EAM model, we obtained results very similar to those obtained with the MPM-B (Fig. 6b). Also in this case, the model is robust to changes in parameter values (Supplementary Note 3 and Fig. 6c,d).

### Shifting the EGFR ubiquitination threshold

**Figure 7:**
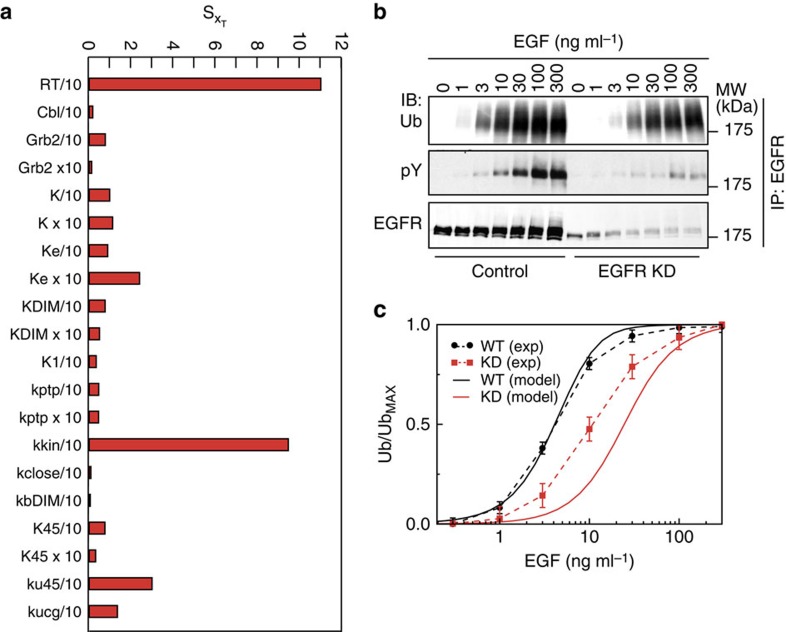
Downmodulation of EGFR levels shifts the ubiquitination dose–response curve. (**a**) We varied each parameter of the EAM 10-fold and computed the sensitivity coefficient S for the EGFR ubiquitination threshold position *x*_*T*_. Only parameters whose variation resulted in a sensitivity coefficient of at least 0.1 are reported. (**b**) EGFR knockdown (KD) in HeLa cells was achieved by transfection with an anti-EGFR siRNA oligo. Control cells were transfected with mismatched oligo. Cells were then treated for 2 min with EGF as indicated. IP and IB were as shown. Quantitation of EGFR ubiquitination is shown in **c**. (**c**) Comparison of model predictions and experimental assessment of the ubiquitination threshold in EGFR-KD (KD) and control (WT) HeLa cells. Simulations were performed with EAM (in the simulation of the EGFR-KD the decrease in EGFR levels was assumed to be 4.2-fold, as determined in ^125^I-EGF saturation binding assays). EGFR ubiquitination is expressed, for each condition, as normalized to the maximum value obtained in that condition (Ub/Ub_MAX_). Experimental data are reported as mean±s.d. from at least three independent experiments.

We next used the EAM to predict parameters whose consistent alteration (that is, 10-fold increase or decrease) would shift the ubiquitination threshold. The analysis identified EGFR kinase rate, *k*_*KIN*_, in agreement with MPM-B, and the total number of EGFRs, *R*_T_, which was not present in the MPM-B (Fig. 7a). Compared with *k*_KIN_, *R*_T_ can be easily manipulated, and thus we validated the prediction by attenuating the expression of EGFR in HeLa cells via incomplete knockdown (Fig. 7b). Under these conditions, an approximately fourfold decrease in EGFR levels (from ∼3 × 10^5^ to ∼7 × 10^4^ EGFRs per cell, measured by ^125^I-EGF saturation binding assay, see Methods) shifted to the right the ubiquitination curve, similarly to what obtained in simulations (Fig. 7c and Supplementary Fig. 7).

In conclusion, guided by the EAM, we identified the total number of surface EGFRs as a key parameter in the control of the position of the ubiquitination threshold and confirmed this prediction experimentally.

### Uncoupling/recoupling of EGFR phosphorylation and ubiquitination

**Figure 8:**
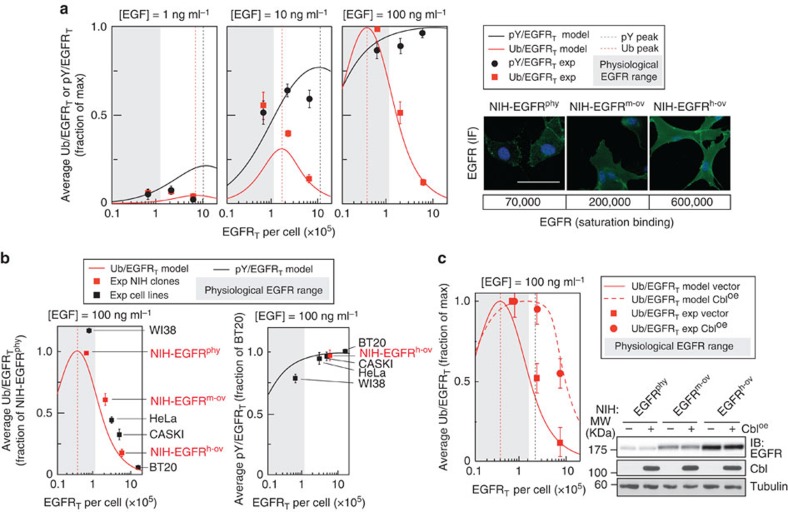
Advanced model for EGFR Ub and pY as a function of EGF concentration and EGFR number. (**a**) Left, relative EGFR phosphorylation (pY/EGFR_T_, black lines) and ubiquitination (Ub/EGFR_T_, red lines) levels, as given by the EAM, for the indicated EGF concentrations. The grey area represents the physiological range of EGFR levels. Dashed lines indicate the maximal phosphorylation and ubiquitination. Data are normalized to the maximum phosphorylation/ubiquitination at 100 ng ml^−1^ EGF. Red squares and black circles represent experimental measurements of EGFR Ub and pY, respectively, obtained by the ELISA-based assay in NIH-EGFR clones with increasing numbers of EGFRs (from lowest to highest: NIH-EGFR^phy^ , physiological EGFR; NIH-EGFR^m-ov^, medium overexpression; NIH-EGFR^h-ov^, high overexpression). Right, representative immunofluorescence images of EGFR surface levels in the indicated NIH-EGFR clones. Scale bar, 18 μm. Bottom, number of surface EGFRs per cell, as measured by ^125^I-EGF saturation binding. (**b**) Left, relative EGFR ubiquitination (left, Ub/EGFR_T_, red line) and phosphorylation (right, pY/EGFR_T_, black line), as given by the EAM, for the indicated EGF concentration, normalized to the maximum. Squares represent experimental measurements of EGFR ubiquitination (left) or phosphorylation (right), obtained by the ELISA-based assay in the indicated NIH-EGFR clones (red) or cell lines with increasing EGFR number (black, see also Supplementary Fig. 9). (**c**) Relative EGFR ubiquitination levels (Ub/EGFR_T_), as given by the EAM, at 100 ng ml^−1^ of EGF under control (red line) or Cbl overexpressing (red dashed line) conditions (modelled as a × 100 increase in line with data in Fig. 4b). Data are normalized to the maximum ubiquitination in each condition. Red squares and circles represent experimental measurements of EGFR ubiquitination, obtained by the ELISA-based assay, in the NIH-EGFR clones (as in **a**) infected with a lentiviral vector driving inducible Cbl overexpression (Cbl^oe^, red circles), or empty vector (Vector, red squares), treated with doxocycline. Right, verification of Cbl overexpression in the indicated NIH-EGFR clones infected with empty vector (−) or vector driving inducible Cbl overexpression (+); tubulin, loading control. Densitometry analysis revealed an ∼80–100-fold Cbl overexpression of Cbl. Experimental data in **a**–**c** are reported as mean±s.d. from at least three independent experiments.

We then interrogated the EAM as to the behaviour of EGFR ubiquitination and phosphorylation over a range of receptor levels spanning from physiological levels (<10^5^ EGFRs per cell) to the pathological levels detected in human tumours (>10^6^ EGFRs per cell), at different EGF concentrations (Fig. 8a). At all EGF concentrations, the average EGFR ubiquitination per receptor (normalized to the maximum ubiquitination obtained at 100 ng ml^−1^ EGF) displayed a bell-shaped curve. Interestingly, the peak is progressively shifted towards lower EGFR surface levels, as the EGF concentration increases (Fig. 8a). In contrast, the peak of phosphorylation per receptor (normalized to the maximum phosphorylation obtained at 100 ng ml^−1^ EGF) shifts towards higher EGFR surface levels (Fig. 8a).

We tested the predictions of the model by measuring the ubiquitination and phosphorylation dose–response curves in cell lines expressing increasing amounts of EGFR. To perform experiments in a homogeneous genetic background, we used NIH 3T3 fibroblasts, which display low endogenous levels of EGFR (∼10,000 EGFRs per cell), and transfected them with an expression vector encoding human EGFR-WT. We then selected three NIH-EGFR clones representative of: (i) the physiological condition (∼7 × 10^4^ EGFRs per cell, EGFR^phy^ cells); (ii) moderate overexpression (∼2 × 10^5^ EGFRs per cell, EGFR^m-ov^ cells); (iii) high overexpression (∼6 × 10^5^ EGFRs per cell, EGFR^h-ov^ cells). The three clones displayed homogenous expression of the receptor at the single cell level (Fig. 8a right), and they displayed a comparable number of Cbl/Grb2 molecules as HeLa cells (Supplementary Figs 8 and 9a). Analysis of the EGF dose response for average EGFR ubiquitination and phosphorylation per receptor in the clones confirmed the predicted uncoupling of the phosphorylation and ubiquitination dose–response curves, as a function of EGFR levels (Fig. 8a).

We extended our analysis to include a number of tumour cell lines displaying increasing amounts of EGFR (HeLa, CASKI, BT20), compared with a normal fibroblast cell line (WI38, Supplementary Fig. 9). Also in this case a dramatic reduction in EGFR ubiquitination was observed, at high EGF concentrations, as a function of EGFR levels (Fig. 8b).

Our model also captured the behaviour of an EGFR mutant (L834R) that occurs in lung cancer (reviewed in ref. 43) and displays increased phosphorylation together with decreased binding to Cbl and ubiquitination^11^,^44^,^45^,^46^,^47^. According to the model, in this setting, Ub and pY curves are uncoupled already at physiological levels of EGFR (Supplementary Fig. 11 and Supplementary Note 4), providing a possible explanation of why this mutant is tumorigenic in absence of receptor overexpression and/or ligand overproduction.

The presence of bell-shaped curves can be explained by the saturation of the reactions leading to both phosphorylation and ubiquitination when EGFRs increase and EGF concentration remains constant. When EGFRs increase above physiological levels, low doses of EGF are diluted among the receptors and the average phosphorylation per rececptor decreases. However, at high EGF concentrations, the ligand is no longer diluted, even at non-physiological levels of EGFR, and the average phosphorylation per receptor does not decrease with EGFR. Ubiquitination, however, decreases also in this case because it requires Cbl, which is limiting. Thus, despite the increase in ‘ubiquitinatable’ EGFRs, the fraction of ubiquitinated receptor decreases at high EGF under conditions of EGFR overexpression.

In support of this interpretation, we could re-establish ubiquitination under conditions of EGFR overexpression, both *in silico* and experimentally, by increasing the levels of Cbl (Fig. 8c and Supplementary Fig. 10).

## Discussion

The ubiquitination threshold is determined through a mechanism of ‘coincidence detection’ of two Tyr residues in the EGFR by a Grb2:Cbl complex. In this mechanism, two components are pivotal: (i) probability and (ii) cooperativity.

Experimental data demonstrated that the phosphorylation of Y1045 and either Y1068 or Y1086 is necessary and sufficient for full EGFR ubiquitination. The concomitant phosphorylation of two Tyr residues occurs via a purely probabilistic process since Tyr residues on EGFR are phosphorylated independently of each other. This implies that, while the probability of individual Tyr phosphorylations increases gradually with the concentration of ligand, the probability of having two, three or more phosphorylated Tyr residues does not. Indeed, multiple phosphorylations will be insignificant at low EGF concentrations and will increase abruptly after a critical value of EGF.

In addition, our modelling efforts demonstrated that probability alone is necessary, but not sufficient, to generate the threshold, with cooperativity also being required. Cooperativity is determined by the presence of Grb2. Although Grb2 does not contribute directly to EGFR ubiquitination, Cbl binding to EGFR and receptor ubiquitination decrease dramatically when Grb2 cannot bind the receptor. Indeed, one clear prediction of the model is that if ubiquitination were to require only one pY, it would not show a threshold behaviour. Such a situation was verified experimentally by the Y1045+ mutant. In this context, Cbl binds to EGFR without the contribution of Grb2. Although greatly diminished, we could detect ubiquitination in this mutant. However, we did not observe any threshold. This is also the expected behaviour of a hypothetical system in which Grb2 and Cbl do not bind cooperatively to EGFR. In this case, the threshold effects, both for Cbl binding and EGFR ubiquitination, are negated, as shown by our simulations.

Downstream of Cbl binding to EGFR, the model did not require additional specific constraints to fully account for the threshold effect. Indeed, it was not necessary to introduce additional layers of control of Cbl ligase activity into the model. This is because Cbl binding to the EGFR displays a threshold profile very similar to the EGFR ubiquitination curve. Thus, we propose that Cbl binding is the major factor determining the generation of the threshold.

In summary, cooperativity necessitates that a combination of multiple Tyr residues is required for ubiquitination, while the probabilistic mechanism guarantees that such a combination arises naturally only at high EGF concentrations.

The MPM-B model consists of two modules: phosphorylation (MPM) and Cbl and Grb2 binding (B). In both modules, we aimed to use the most standard mathematical representations of the chemical reactions: phosphorylation is described as a standard multisite chain of reactions, Grb2 and Cbl binding is simple mass action, and cooperativity is introduced in the canonical way. The key observations on which we built the MPM-B are directly embedded in the wiring of the network: (i) in the model, the dissociation constant of Cbl for EGFR decreases in the presence of Grb2, in agreement with the fact that Grb2 stabilizes the binding between EGFR and Cbl; (ii) in the free-free regime phosphorylation of Tyr residues is inherently independent of their number (which also allowed us to restrict the analysis to the three critical Tyr residues); (iii) the model traces back the ubiquitination threshold to Cbl binding to the EGFR. For the choice of parameters, we note that many of them were either well constrained or were experimentally determined. EGFR activation was modelled by introducing a phenomenological function, which spared us from introducing many parameters that describe the actual dynamics of EGFR activation. Phosphorylation was characterized by fitting the dose–response curves. The binding of Cbl and Grb2 to EGFR depended on protein concentrations and binding affinities: protein concentrations were carefully measured, while binding affinities were indirectly determined by fitting ubiquitination dose–response curves under various experimental conditions, within well-defined experimental constrains taken from published studies (Supplementary Table 1).

Not surprisingly, the relative simplicity of the model came with some cost. Despite the overall agreement, there are areas in which we noticed some divergences between modelling and experiments. First, the experimental curves of EGFR ubiquitination always showed a higher degree of sigmoidicity than the *in silico* simulations, suggesting that the analogical-to-digital conversion operating in real life is more efficient than in model predictions. Second, the overexpression of Cbl in HeLa cells does not reproduce model predictions in exact quantitative terms. In this case, model predictions and experiments can be made to agree quantitatively only if we assume that 1/3 of the overexpressed Cbl molecules are indeed capable of binding to the receptor. The possibility exists that in our model we lack some parameters of the Cbl regulatory network (for example, phosphorylation, homo- and heterodimerization, counteraction by deubiquitinases^40^,^48^,^49^,^50^) that, although not essential to generate the threshold, might help to refine the mathematical description of the system. Future versions of the model will deal with these discrepancies.

The threshold effect predicted by the MPM-B was robust to changes in parameter values, a property that was verified in real cells. In particular, deletion of Tyr residues (except for Y1068, Y1086 and Y1045), Cbl overexpression and the expression of a Cbl-dominant-negative mutant Cbl70Z (modelled as downregulation) did not displace the threshold, in the modelled and experimental settings. Moreover, although the biological system, and the model as well, are not at steady state, qualitatively the properties of the model (that is, the threshold, its dependency on EGFR level and the lack of effect of perturbations of Cbl on the threshold) do not strictly depend on time (not shown). Robustness is the result of different mechanisms operating in the different modules of the network: Tyr residues do not affect each other's phosphorylation and Cbl overexpression does not affect the threshold because Cbl is limiting in the system; thus, an increase in Cbl leads to the same proportional increase in EGFR ubiquitination for all EGF concentrations.

To identify perturbations that might alter the position of the threshold, we had to expand the MPM-B to create the EAM, which includes the molecular details of EGFR activation that, in the MPM-B, were hidden in the phenomenological relationship between EGF and EGFR activation. Interestingly, the EAM also displayed a remarkable robustness to parameter changes. Accordingly, most of the parameters of the model could not be obtained simply by fitting the experimental data (Supplementary Note 2), but they had to be constrained experimentally; for this reason we either measured them directly or obtained their values from the literature (see Supplementary Table 1). The increased complexity of the EAM led to the generation of a prediction, experimentally verified, that the position of the threshold can be displaced by reducing EGFR levels, since it reduces the capability of EGFRs to dimerize and to be activated. A key element is that the dimers are not stable, being continuously formed and disrupted, in agreement with recent data^51^.

An important validated prediction of the EAM concerns the response of EGFR ubiquitination to variations in EGFR levels. The system exhibits a complex behaviour that depends both on receptor levels and ligand concentration, and appears to have evolved in such a way that the maximal response is in the physiological range of EGFR levels. At these levels (indicated by a shaded area in Fig. 8a), the system responds to increasing EGF concentration with a congruent increase in the relative levels of EGFR ubiquitination. Since EGFR ubiquitination is coupled to the internalization of the receptor through the NCE pathway^21^,^23^, this means that over the rather wide physiological range of EGFR (spanning one order of magnitude), the cell is well equipped to respond to increasing ligand concentration with increased receptor degradation, to protect itself from overstimulation. The situation is rather different if one considers supraphysiological EGFR levels. Under these conditions, the model predicted, and experiments confirmed, that the relative ubiquitination of the receptor decreases much more abruptly than receptor phosphorylation (Fig. 8a), which would translate in a faster relative attenuation of NCE-mediated degradation with respect to signalling.

These findings are highly relevant to human cancers. In tumours, EGFR is frequently overexpressed. Our data show that, both in an isogenic model and in tumour cell lines, there is a progressive uncoupling of EGFR phosphorylation and ubiquitination at the supraphysiological EGFR level. The effect is evident for EGF concentrations above 1 ng ml^−1^: a condition frequent in human tumours, in which overexpression of EGFR is frequently accompanied with overproduction of its ligands^52^,^53^,^54^ (and references therein). In other terms, the optimization of the system for physiological EGFR levels also harbours an intrinsic weakness, which is exploited by cancer cells to obtain a proliferative advantage. In turn, this point of weakness, now identified, might constitute a suitable point of intervention for therapeutic purposes.

## Methods

### EGFR models

We simulate three different models, with increasing complexity by means of ordinary differential equations. MPM (Fig. 2d,e) keeps track of EGFR phosphorylation only. EGFR activation is described as a Hill function of [EGF]. MPM-B (Figs 4 and 5 and Supplementary Figs 2,3b,c,4 and 5) is MPM with the addition of Grb2 and Cbl binding, to EGFR and to each other. In EAM (Figs 6b–d,7a,c and 8a–c and Supplementary Fig. 11), the complete model, EGFR activation is described in molecular detail, while the binding reactions for Cbl and Grb2 are the same as in MPM-B. Further details for the models used in the study can be found in the Supplementary Note 1.

Simulations were carried out with XPP-AUT (http://www.math.pitt.edu/~bard/xpp/xpp.html). Initial conditions were chosen to have all species at the steady state, in the absence of EGF stimulus. At time zero, we stimulate the cells with EGF. Parameter estimation was performed using a global optimization method based on simulated annealing, followed by a local optimization based on the simplex method. SBML files are available at biomodels.org (EAM: MODEL1505190000; MPM-B: MODEL1505190001).

### Reagents and antibodies

EGF was from PeproTech; ^125^I-EGF from PerkinElmer. Antibodies were as follows: an in-house polyclonal anti-EGFR against aa 1172–1186 of human EGFR (working concentration in immunoblot, IB: 0.06 μg ml^−1^); a monoclonal anti-EGFR (m108 hybridoma, directed against the extracellular domain of human EGFR, American Type Culture Collection (ATCC)^55^, working concentration: in immunofluorescence, IF, 3 μg ml^−1^); anti-pY (clone 4G10, Millipore #05–231; IB working concentration: 1 μg ml^−1^; working concentration in immunoprecipitation, IP, 10 μg for 1 mg of lysate); anti-Ub (P4D1, Santa Cruz Biotechnology, #sc-8017, used in all anti-Ub IBs at a working concentration of 0.2 μg ml^−1^); anti-Ub FK2 (Enzo Life Science, #BML-PW8810, used in all ELISA assays, for working concentration see section below); anti-EGFR phospho-specific antibodies (Cell Signaling, pY1045 #2237, pY1068 #2234, IB working dilution: 1:1,000); in-house polyclonal anti-alpha-tubulin raised in rat (MAB1864, clone YL1/2, IB working concentration: 1.87 mg ml^−1^); anti-Grb2 (Clone 81, BD Biosciences, #610112, IB working concentration: 0.125 μg ml^−1^); anti-Cbl polyclonal (clone 15, Santa Cruz Biotechnology, #sc-170, IB working concentration: 0.4 μg ml^−1^) and monoclonal (BD Bioscience, #610442, IF working concentration: 0.5 μg ml^−1^) antibodies.

### Constructs and cell lines

HeLa, NIH 3T3, WI38, CASKI and BT20 cells were obtained from ATCC and cultured following the instructions of ATCC. All human cell lines were authenticated at each batch-freezing by STR profiling (StemElite ID System, Promega). All cell lines were tested for mycoplasma at each batch-freezing using PCR^56^ and biochemical assay (MycoAlert, Lonza). HeLa cells transfected with Cbl or Cbl70Z were previously described^57^. The EGFR mutants were stably expressed (see ref. 21) in NR6 cells, which are mouse fibroblasts devoid of endogenous EGFR^58^. EGFR surface expression in the transfectants was assessed by saturation binding with ^125^I-EGF (ref. 21).

Human EGFR WT was stably expressed in NIH 3T3 cells. Single clones were isolated and characterized for EGFR surface expression levels and homogeneity by immunofluorescence (IF; Fig. 8a, right panel). Briefly, cells were fixed in 4% paraformaldehyde and stained (before permeabilization) with the monoclonal anti-EGFR antibody that recognizes the extracellular domain (m108). Cells were then incubated with Alexa488-conjugated secondary antibody (Molecular Probes) and stained with 4,6-diamidino-2-phenylindole. Clones expressing different levels of surface EGFR, assessed by saturation binding with ^125^I-EGF, were selected for further analysis (Fig. 8a, right panel). These clones were subsequently infected with an inducible lentiviral construct carrying cDNA encoding human c-Cbl (pSLIK-neo vector^59^). This construct was engineered starting from a c-Cbl cDNA kindly provided by Y. Yarden (Weizmann Institute, Israel). All clones were sequence-verified; details are available on request.

Stable bulk populations were obtained after 10 days of neomycin treatment (400 μg ml^−1^). On doxycycline treatment (0.5 μg ml^−1^ for 16 h), Cbl was expressed at levels ∼80–100-fold greater than those of the endogenous protein (assessed by densitometry analysis of the IB at different exposures, Fig. 8c) and displayed a homogeneous expression level (assessed by IF, Supplementary Fig. 10). The same clones infected with the empty vector were treated with doxycycline in the same way and were used as control (Fig. 8c).

EGF treatment was for 2 min at 37 °C. Under these conditions, EGFR internalization is negligible and the observed phosphorylation and ubiquitination events occur primarily at the PM^21^.

### Saturation binding assay

Serum-starved cells were incubated on ice for 6 h in the presence of ^125^I-EGF (100 ng ml^−1^: 10 ng ml^−1^ of ^125^I-EGF plus 90 ng ml^−1^ of cold EGF) in serum-free medium supplemented with 0.5% BSA. Cells were then washed three times with ice-cold PBS and solubilized in 1 M NaOH. After correction for the hot/cold dilution, the number of receptors on the surface was deduced from the specific activity of the labelled ligand. Nonspecific binding was measured in the presence of a 300-fold excess of cold EGF, and was never >3–10% of the total counts.

### EGFR knockdown in HeLa cells

Silencing of EGFR in HeLa cells was achieved by double transient transfection, using OligofectAMINE (Invitrogen), of the following short interfering RNA (siRNA) oligo (Stealth siRNA from Invitrogen): 5′-CCGCAGCAUGUCAAGAUCACAGAUU-3′. The sequence of the mismatched control oligo was: 5′-CCGACGUGUAACUAGCACGACAAUU-3′. Cells were analysed 48 h after the second transfection.

### Biochemical assays

Cell lysis was performed in RIPA buffer (50 mM Tris-HCl, 150 mM NaCl, 1 mM EDTA, 1% Triton-X100, 1% sodium deoxycholate, 0.1% SDS), plus protease inhibitor cocktail (CALBIOCHEM) and phosphatase inhibitors (20 mM sodium pyrophosphate pH 7.5, 50 mM NaF, 2 mM PMSF, 10 mM Na_3_VO_4_ pH 7.5). To exclude the presence of co-immunoprecipitating proteins, for the ELISA assays, lysis was performed in RIPA buffer containing 1% SDS, followed by clarification for 1 h at 120,000 g and dilution to a final SDS concentration of 0.2%. Immunoprecipitations (IPs) were performed starting from 500 μg of lysates, with the appropriate antibodies. IPs were incubated for 2 h at 4 °C and then Protein G (Zymed) was added for 1 h. After extensive washing with RIPA buffer, IPs were eluted in Laemmli buffer. Immunoblotting (IB) was performed as described previously^57^,^60^. Quantification of blots was performed with Photoshop.

### Determination of the number of Cbl and Grb2 molecules

To calculate the number of Grb2 or Cbl molecules in HeLa (Fig. 3b), NIH-EGFR (Supplementary Fig. 8) or CASKI/BT20 (Supplementary Fig. 9d) cell lysates, we compared signal intensities in anti-Grb2 or anti-Cbl IB of increasing amounts of cellular lysate with known amounts of purified Grb2 or Cbl protein. From the signal comparison and taking into account Avogadro’s number, we calculated the number of Grb2 and Cbl molecules per μg of lysate. After an additional correction for the number of HeLa or NIH-EGFR cells corresponding to 1 μg of lysate (measured in the same experiment), we obtained the total number of Grb2 and Cbl molecules per cell. To calculate the number of active Cbl molecules (Cbl pY), we IP Tyr-phosphorylated Cbl, using the anti-pY antibody, from increasing amounts of lysate (prepared in RIPA buffer containing 1% SDS) from cells stimulated with EGF (100 ng ml^−1^) for 2 min. Immunoprecipitates were then IB with anti-Cbl, and the signal intensities of the anti-Cbl bands were compared with the signal intensities of anti-Cbl bands in IB cellular lysates and purified Cbl protein. We corrected the obtained values for the efficiency of IP (estimated >90%) and obtained the % of Cbl in the lysate that is phosphorylated (active) on stimulation with EGF (100 ng ml^−1^) for 2 min.

### ELISA assays for EGFR ubiquitination and phosphorylation

For the ELISA-based assays shown in Fig. 8 and Supplementary Fig. 7, we used the Dissociation Enhanced Lanthanide Fluoroimmunoassay technology from Perkin Elmer. This technology is based on sandwich recognition of a target protein by a capture antibody and a detection antibody. The capture antibody is immobilized on a solid surface (microwells) directly through non-covalent bonds. After the addition of the analyte (appropriate cellular lysate), the detection of signals relies on a lanthanide (Europium)-conjugated antibody that is able to produce a fluorescent signal on enhancement with acidic enhancement buffer. Lysates were prepared in RIPA/1% SDS buffer and diluted to 0.2% SDS before the incubation step. Plate preparation, analyte incubation and antibody detection were performed according to the manufacturer’s instructions (additional details are in ref. 21).

As capturing antibodies the following were used: monoclonal antibodies against Ub (FK2, 5 μg ml^−1^), pY (4G10, 5 μg ml^−1^), pY1068 (1 μg ml^−1^), pY1086 (1 μg ml^−1^) or EGFR (m108, 1 μg ml^−1^); as detecting antibody the following were used: in-house polyclonal anti-EGFR directed against aa 1172–1186 of human EGFR (1 μg ml^−1^).

### Densitometry and statistical analysis

Quantification of blots was performed with Photoshop. In all experiments, densitometry was performed on different exposures of the blots and results were obtained in the linear phase of the exposure. Average results, calculated from at least three independent experiments, are shown. Error bars in the plots represent the s.e.m.

## Additional information

**How to cite this article:** Capuani, F. *et al.* Quantitative analysis reveals how EGFR activation and downregulation are coupled in normal but not in cancer cells. *Nat. Commun.* 6:7999 doi: 10.1038/ncomms8999 (2015).

## Supplementary Material

Supplementary Information Supplementary Figures 1-11, Supplementary Tables 1-2, Supplementary Notes 1-4 and Supplementary References

Supplementary Dataset Multisite Phosphorylation Model
