# Supplementary material for: Quantitative analysis reveals how EGFR activation and downregulation are coupled in normal but not in cancer cells
Source: Nat Commun. 2015 Aug 12;6:7999. doi: 10.1038/ncomms8999 (PMC4538861; doi:10.1038/ncomms8999)
Supplement: Supplementary Information — Supplementary Figures 1-11, Supplementary Tables 1-2, Supplementary Notes 1-4 and Supplementary References [file ncomms8999-s1.pdf]

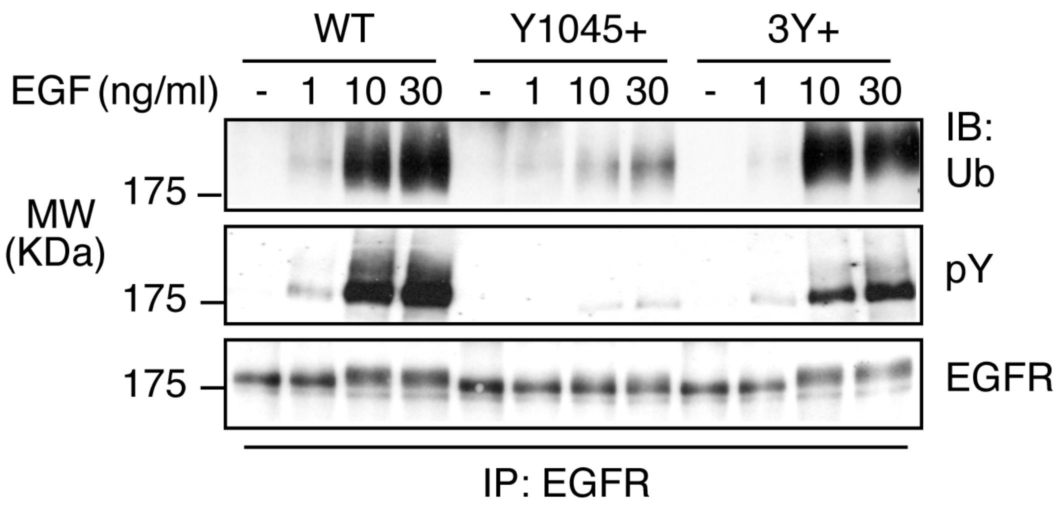

Supplementary Figure 1. Dose response analysis of Ub and pY of EGFR-WT and the Y1045+ and 3Y+ add-back mutants. NR6 cells stably expressing the indicated mutants were stimulated for 2 min with increasing concentrations of EGF, as indicated. Lysates were subjected to IP and IB as shown. The ratio of total pY of EGFR-WT *vs.* EGFR-3Y+ was determined by densitometry analysis and shown in Fig. 1f and Fig. 2e of the main text.

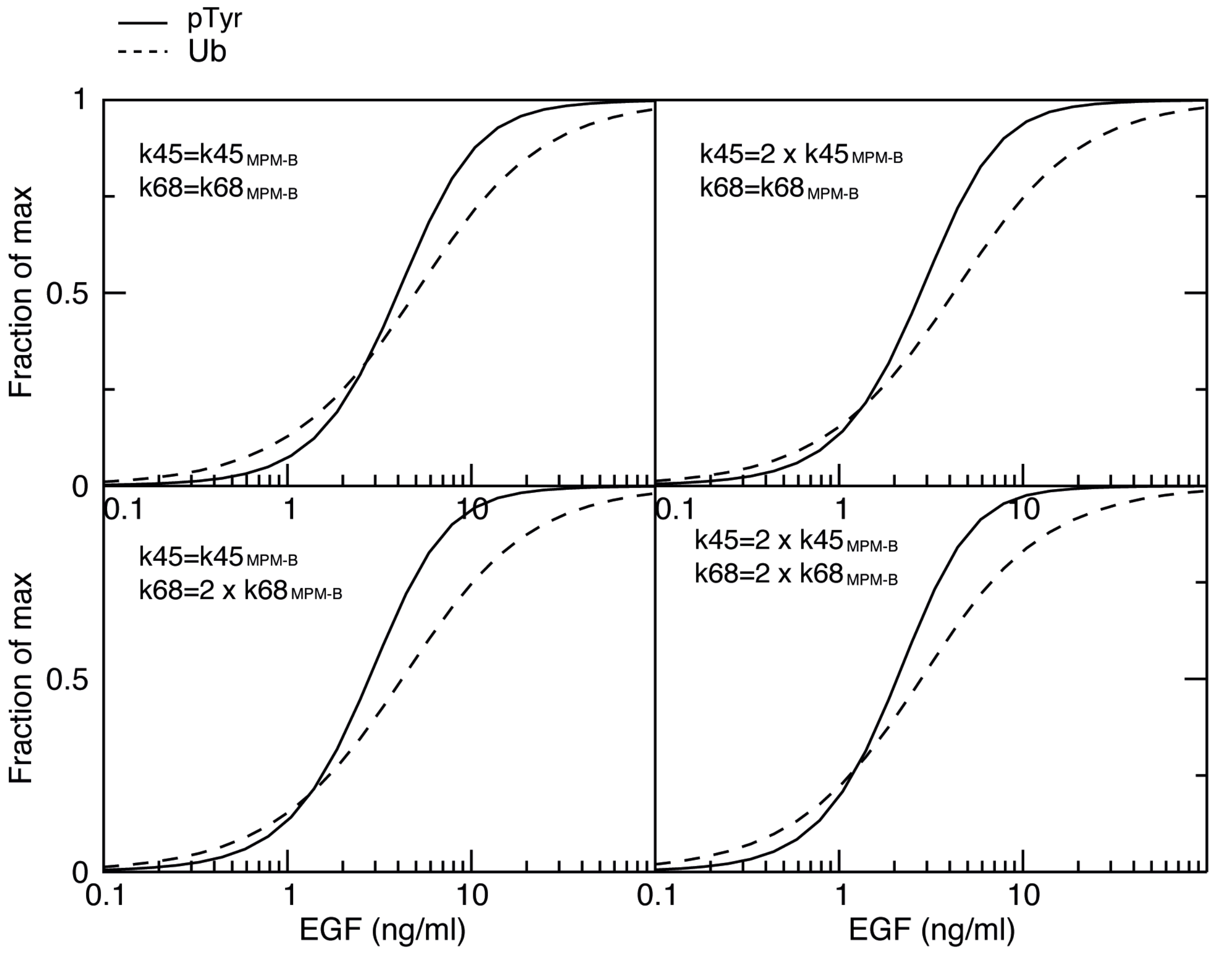

Supplementary Figure 2. Relaxing the assumption of equal phosphorylation rates in MPM-B. Dose-response curves for phosphorylation (solid line) and ubiquitination (dotted line) differentiating the phosphorylation of Y1045 and Y1068. In the simulation, the ratio of $k_{\mathrm{KIN}}^{\mathrm{MAX}}$ for Y1045 (i.e. $k45$) and for Y1068 (i.e., $k68$) can vary between 2 and 0.5. Simulations are performed with MPM-B. The reference value is $k_{\mathrm{KIN}}^{\mathrm{MAX}}$ (i.e., $k45_{MPM-B} = k_{\mathrm{KIN}}^{\mathrm{MAX}}$, $k68_{\mathrm{MPM-B}} = k_{\mathrm{KIN}}^{\mathrm{MAX}}$).

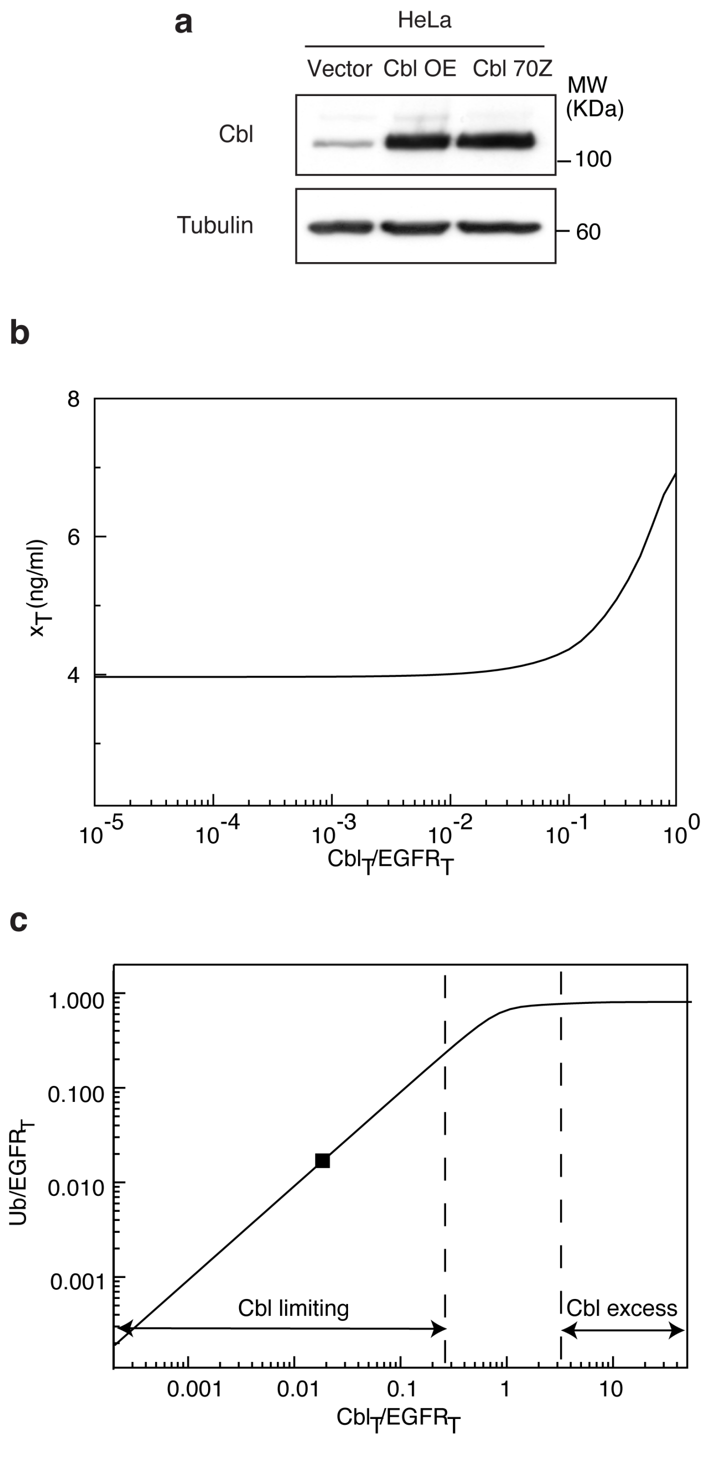

Supplementary Figure 3. Effect of Cbl over- or under-expression on EGFR ubiquitination. A. Lysates of HeLa cells stably transfected with Cbl-WT (Cbl overexpression, OE), Cbl70Z, or empty vector (Vector), were analyzed by IB as indicated. Densitometry analysis revealed a ~7-fold increase in Cbl expression levels in Cbl-OE and Cbl70Z transfectants compared with control cells. **B.** Dependence of the ubiquitination threshold-position x_T on the total Cbl concentration [expressed as normalized to the total EGFR concentration ($Cbl_T/EGFR_T$)] for an EGF concentration of 100

ng/mL. Calculations were performed using MPM-B and parameters given in Supplementary Table 1 (with the obvious exception of Cbl_T). **C.** Dependence of EGFR ubiquitination [expressed as normalized to the total EGFR concentration ($Ub/EGFR_T$)] on the total Cbl concentration [expressed as normalized to the total EGFR concentration ($Cbl_T/EGFR_T$)]. The black square indicates the amount of total Cbl in EGFR-3Y+. MPM-B model and parameters as in B.

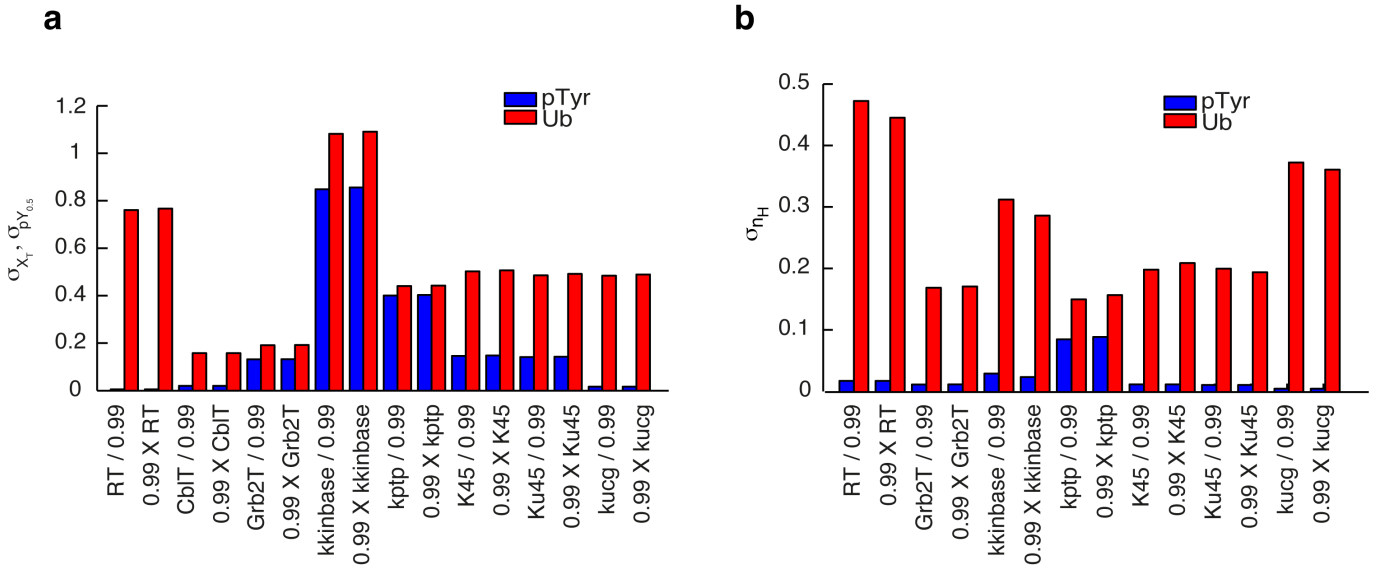

Supplementary Figure 4. Sensitivity analysis of the MPM-B model. A-B. Sensitivity analysis. We varied each parameter by 1% and computed the sensitivity coefficient σ for the Hill coefficient n_H (A) and for both the phosphorylation half-maximum level $pY_{0.5}$ and ubiquitination threshold x_T (B), for EGFR phosphorylation (blue) and ubiquitination (red).

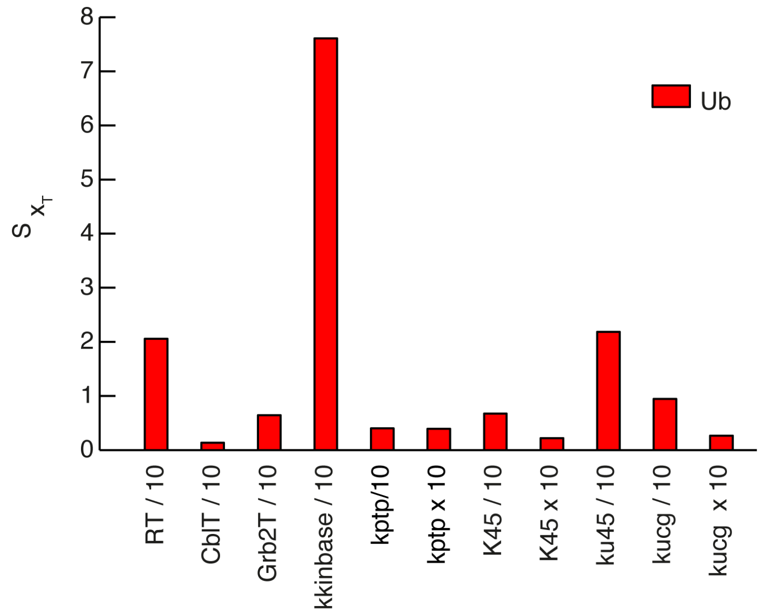

Supplementary Figure 5. Prediction of parameters whose modulation changes the position of the thresholod using the MPM-B. We varied each parameter of the MPM-B 10-fold and computed the sensitivity coefficient S for the EGFR ubiquitination threshold position x_T. Only parameters whose variation resulted in a sensitivity coefficient of at least 0.1 are reported.

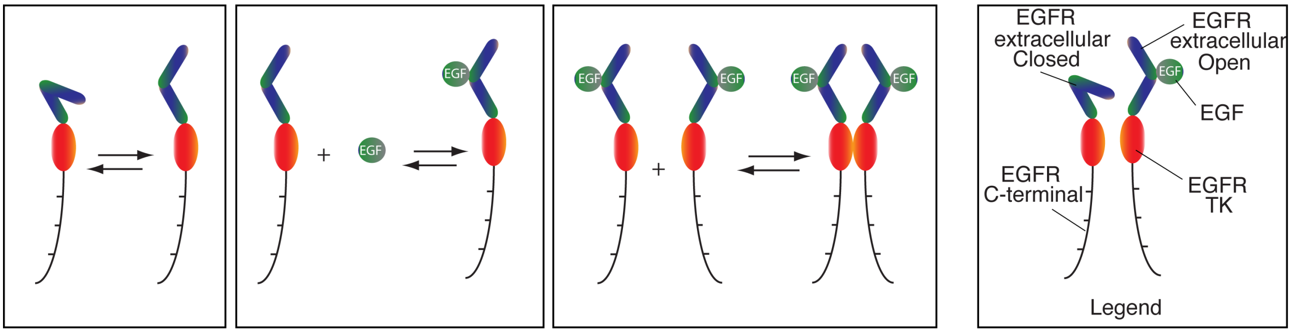

Supplementary Figure 6. Schematic representation of the EGFR and its activation dynamics. Left, opening of the extracellular domain. Middle, EGF binding to the open EGFR conformation (this reaction can also occur for the closed EGFR conformation). Right, EGFR activation requires dimerization with an EGFR bound to EGF, which can occur between any pair of receptors in the extended conformation. A legend is shown on the far right.

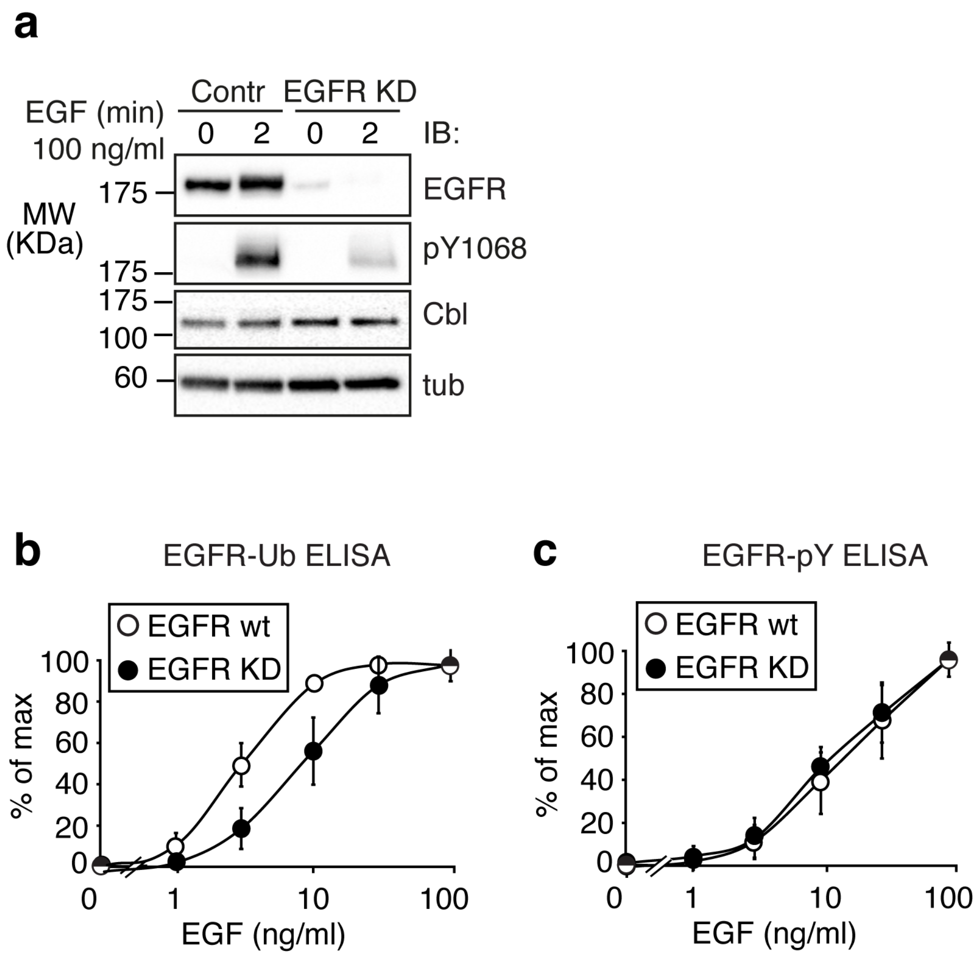

Supplementary Figure 7. EGFR ubiquitination and phosphorylation dose-response curves upon downmodulation of EGFR measured by ELISA. A. HeLa cells were subjected to EGFR-knockdown (KD) or transfected with control oligos. IB was as shown; tubulin (tub) was used as a loading control. Note that Cbl levels do not change upon EGFR KD. **B, C.** Lysates from control or EGFR-KD HeLa cells (as in A) were treated for 2 min with EGF as indicated. EGFR ubiquitination (B) and phosphorylation (C) were measured by ELISA assay, as described in the Methods.

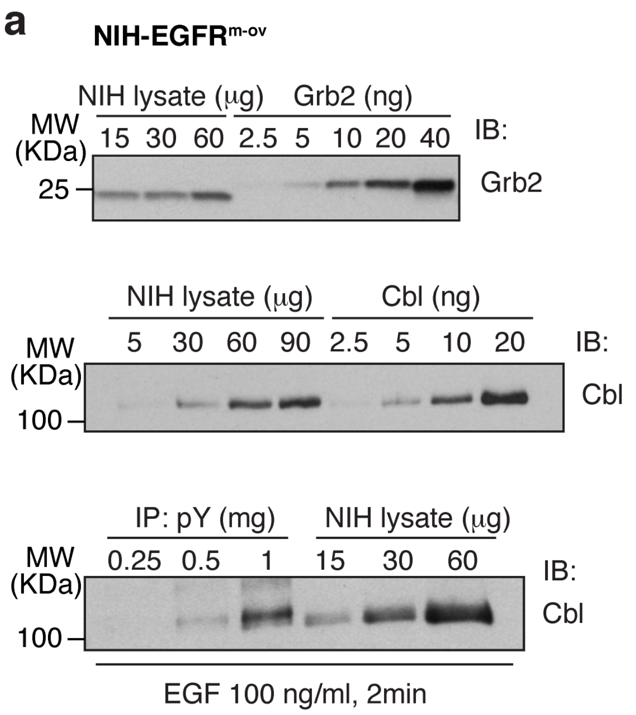

b

	N molecules/cell (x10^3)	
	**Hela wt**	**NIH-EGFR$^{m\text{-}ov}$**
EGFR	300 (-/+50)	200 (-/+50)
Grb 2	1000 (-/+260)	800 (-/+120)
Cbl tot	150 (-/+25)	130 (-/+8)
Cbl-pY	5.2 (-/+0.75)	6 (-/+1.5)

Supplementary Figure 8. Quantitation of the number of Grb2 and Cbl molecules in NIH3T3 cells overexpressing EGFR. A. Increasing amounts of NIH-EGFR$^{m\text{-}ov}$ cell lysate (NIH3T3 clone with medium EGFR overexpression, see Fig. 8a right) were subjected to IP/IB as indicated and compared with increasing amounts of *in vitro* purified Grb2 (top panel) or Cbl (middle panel) proteins. NIH-EGFR$^{m\text{-}ov}$ was chosen because it has similar EGFR level as HeLa cells and, thus, it can be directly compared to HeLa in terms of Cbl-pY amount. For a direct comparison of the three NIH clones see Supplementary Fig. 9a. **B.** Table indicating the amount of the critical components of the EGFR ubiquitination reaction in NIH-EGFR$^{m\text{-}ov}$ cells. The number of surface EGFR was measured by ^{125}I-EGF saturation binding (see Methods). Data are expressed as number of surface EGFRs/cell. The number of Grb2 and Cbl molecules, and the number of active Cbl molecules (Cbl pY) in NIH-EGFR$^{m\text{-}ov}$ cells was calculated as described in Methods. IBs are representative of at least three independent experiments.

a

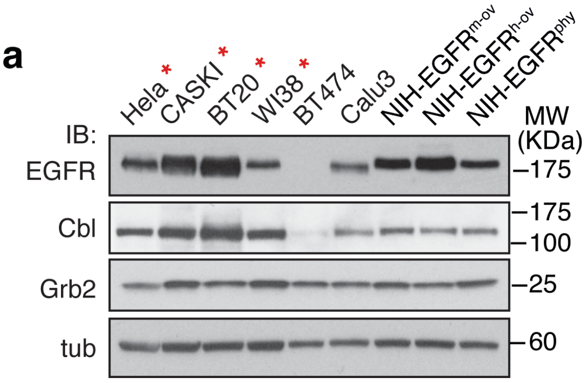

b

Cell line	Origin	EGFRs/cell (x10^3)
WI38	Normal lung fibroblasts	80
HeLa	Cervix adenocarcinoma	250
CASKI	Cervix epidermoid carcinoma	400
BT20	Breast carcinoma	1,200

c

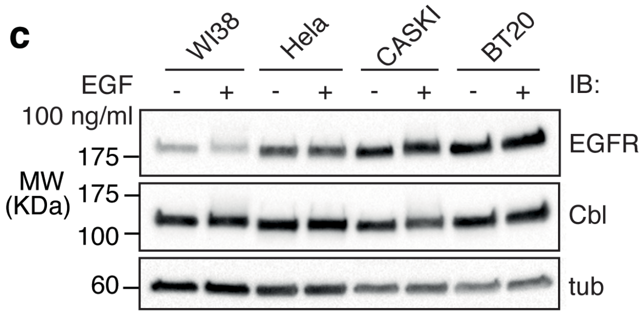

d

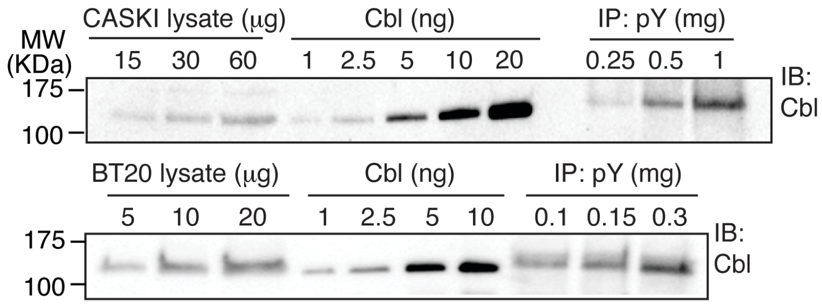

	N molecules/cell (x10^3)	
	**CASKI**	**BT20**
Cbl tot	190	210
Cbl-pY	2.7	15

Supplementary Figure 9. Characterization of the cell lines used in Figure 8 of the main text.

A. Lysates from the indicated cell lines were IB as shown. Asterisks indicate cell lines with levels of Cbl/Grb2 comparable to those in HeLa cells, but with different EGFR levels (see also panel B),

which were selected for the Ub/pY analysis performed in Fig. 8b. **B.** Table indicating the origin of the cell lines used in Fig. 8b, which have been characterized for the number of surface EGFR by saturation ^{125}I-EGF binding assay (see Methods). **C.** EGFR and Cbl levels were evaluated by IB in the indicated cell lines at steady state or upon stimulation with 100ng/ml EGF for 2 minutes. Tubulin was used as loading control. Cbl levels are comparable in the different samples. **D.** Increasing amounts of CASKI (top) or BT20 (middle) cell lysate were subjected to IP/IB as indicated, and compared with increasing amounts of *in vitro* purified Cbl protein. Bottom, table indicating the number of Cbl-pY molecules/cell in CASKI or BT20, calculated by comparing the signal intensities of the anti-Cbl bands in the IP anti-PY with the signal of the purified Cbl protein (see Section "Determination of the number of Cbl and Grb2 molecules" in Methods of the main text). Quantitation of IBs was performed with Photoshop. Note that, in our model, tailored for HeLa cells, the limit where Cbl becomes limiting is $Cbl_{\mathrm{T}}/EGFR_{\mathrm{T}} \lesssim 0.1$ (Supplementary Fig. 3c). This is the case in both CASKI (2.700 / 400.000) and BT20 (15.000 / 1.200.000).

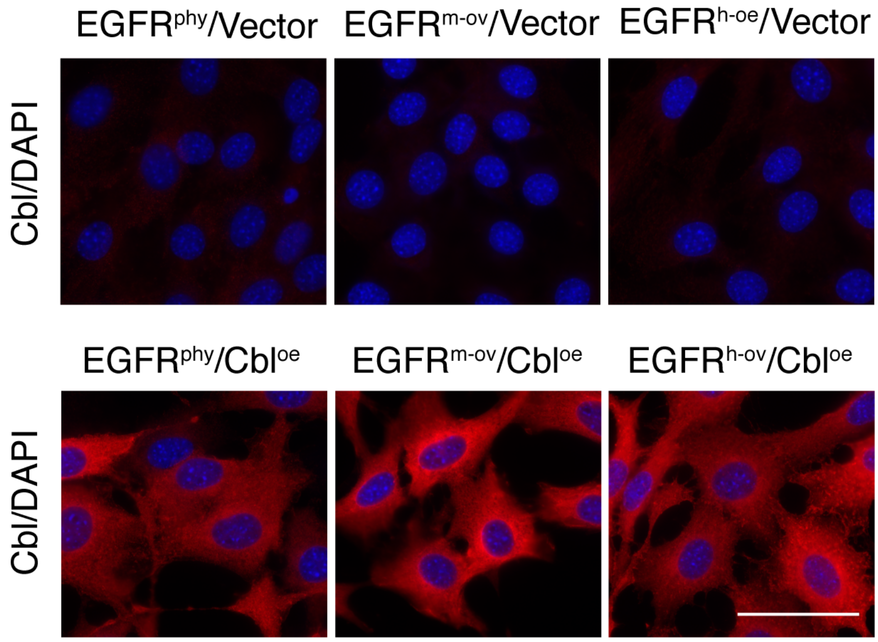

Supplementary Figure 10. Additional controls for Cbl overexpression in NIH-EGFR clones. Immunofluorescence staining of Cbl (red) in the indicated NIH-EGFR clones (used in Fig. 8 of the main text), infected with empty vector (Vector) or vector encoding Cbl (Cbl^{oe}, Cbl overexpression). Comparable exposures are shown. Note that endogenous Cbl is not detected at this exposure, while it gives a punctuate staining pattern at higher exposures (not shown). Clones with Cbl^{oe} displayed homogeneous Cbl expression in >95% of cells. Nuclei are stained with DAPI (blue). Bar 18 μm.

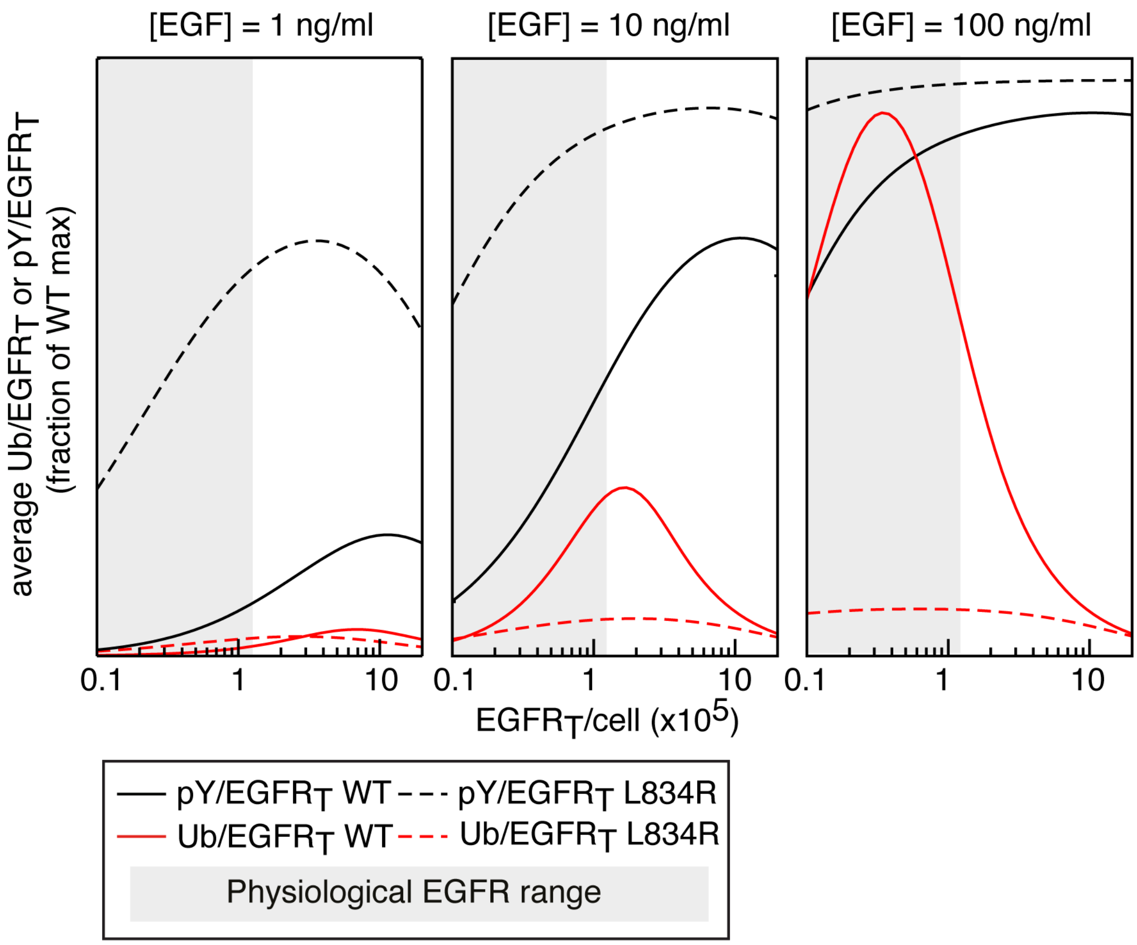

Supplementary Figure 11. Behavior of Ub and pY of L834R EGFR mutant as a function of EGF concentration and EGFR number.

Relative L834R EGFR phosphorylation ($pY/EGFR_{\mathrm{T}}$, black lines) and ubiquitination ($Ub/EGFR_{\mathrm{T}}$, red lines) levels, as given by the EAM, are shown for the indicated EGF concentrations. The gray area represents the physiological range of EGFR levels. Data are normalized to the maximum phosphorylation/ubiquitination of EGFR WT obtained at 100 ng/ml EGF. Dashed lines represent mutant curves; continuous lines represent WT curves (the same as in Fig. 8a).

Supplementary Table 1

Reaction type or species	Symbol	Optimization Range (Model)	Value (Model)	Optimization Range (Typical units)	Value (Typical units)	Reference
Total concentration of EGFR[a]	R_T	N.O.	0.83 nM	N.O.	250000 molecules	This manuscript
Concentration of Cbl available for EGFR interaction[b]	Cbl_T	N.O.	$1.5x10^{-2}$ nM	N.O.	5000 molecules	This manuscript
Total concentration of Grb2[c]	$Grb2_T$	N.O.	3.3 nM	N.O.	1000000 molecules	This manuscript
Hill coefficient for phopshorylation curve[d]	n_H	0.5-1.5	1.13	0.5-1.5	1.13	This manuscript
Half-maximum phosphorylation of EGFR[d]	H	0.1-10 nM	4.6 nM	0.58-58.8 ng/ml	27 ng/ml	This manuscript
Maximum phosphorylation rate constant[d]	k_{KIN}^{MAX}	0.005—10 s^{-1}	0.0759 s^{-1}	0.005—10 s^{-1}	0.0759 s^{-1}	> 0.005 s^{-1} [1]; 0.0733 s^{-1} [2]; > 0.075 s^{-1}/ 2 s^{-1} [depending on model in[3]]; 0.2 s^{-1} [4]; 1s^{-1} [5]
Dephosphorylation rate constant[e]	k_{PTP}	N.O.	0.016 s^{-1}	N.O.	0.016 s^{-1}	0.016 s^{-1} [3]; <0.1 s^{-1} [1]; 0.01 s^{-1} [6]**;** 0.13 s^{-1} [4]; 1s^{-1} [5]
Kd of of pY1068/pY1086 and Grb2 binding (and of pY1045 and Cbl)[f]	$K45$ ($K68$)	0.2—150 nM	0.2 nM	0.01—7 μM	0.01 μM	0.4 μM[7]; 0.7 μM[8]; 0.1 μM[9]; 0.35 μM[10];
Kd of Cbl and Grb2 binding[g]	Kcg	0.006—30 nM	0.006 nM	0.0003—1.5 μM	0.3 nM	1.48 nM[11]; 400 nM[8]; 1.8 μM[12]; 300–500 μM [13]; 4 μM and 120 μM [Nter-SH3 and CterSH3of Grb2[9]]
Rate constant of dissociation of pY1068/pY1086 and Grb2 pY1045 and Cbl (and of pY1045 and Cbl)[f]	$ku45$ ($ku68$)	0.001—40 s^{-1}	0.001 s^{-1}	0.001—40 s^{-1}	0.01 s^{-1}	0.2 s^{-1} [8]; 0.4-4 s^{-1} [9]
Rate constant of dissociation of Cbl and Grb2[g]	$kucg$	0.01—100 s^{-1}	0.3 s^{-1}	0.01—100 s^{-1}	0.3 s^{-1}	Not available
Localization factor [h]	f_{LOC}	10—10^5 nM	$2x10^4$ nM	0.5 μM —5 mM	1 mM	This manuscript
Maximum ubiquitination	Ub_{MAX}	0.0005—0.05 nM	0.0143 nM	0.003-0.3 ng/ml	0.083 ng/ml	This manuscript
Maximum phosphorylation (MPMB)	$pYmax$	0—2.5 nM	2.28 nM	0-15 ng/ml	13 ng/ml	This manuscript
**EAM parameters**						
Maximum phosphorylation (EAM)	$pYmax$	0—2.5 nM	2.35 nM	0-15 ng/ml	13.8 ng/ml	This manuscript
K_d of EGF-EGFR binding (high affinity)[i]	K	0.1—20 nM	3.4 nM	0.1—20 nM	3.4 nM	0.2 nM[14], 4.3 nM[15]; 0.67 nM[16]; 20 nM[6]; 20 nM[17]
EGF-EGFR association rate constant (to a preformed EGFR dimer)[i]	k_b	0.02—20 $nM^{-1}s^{-1}$	5 $nM^{-1}s^{-1}$	0.02—20 (5) $nM^{-1}s^{-1}$	5 $nM^{-1}s^{-1}$	0.2–0.4 $nM^{-1}s^{-1}$ (first EGF moiety), 2 $nM^{-1}s^{-1}$ (second EGF moiety)[18]
K_d of EGF-EGFR binding (low affinity)[i]	$K1$	N.O.	50 nM	N.O.	50 nM	2.9 nM[14]; 600 nM[17]
EGF-EGFR association rate constant (low affinity)[i]	$k1_b$	N.O.	$3x10^{-3}$ $nM^{-1}s^{-1}$	N.O.	0.003 $nM^{-1}s^{-1}$	0.001 $nM^{-1}s^{-1}$ [15]; 0.003 $nM^{-1}s^{-1}$ [16]; 0.004 $nM^{-1}s^{-1}$ [18]; 0.001 $nM^{-1}s^{-1}$ [4]; $3x10^{-3}$ $nM^{-1}s^{-1}$ [6]
Equilibrium ratio of EGFR closed/open[l]	K_e	N.O.	30	N.O.	30	20-30[17]
Rate of closing of extracellular EGFR domain[l]	k_c	N.O.	10 s^{-1}	N.O.	10 s^{-1}	Not available
As k_c when EGFR has EGF bound[l]	$k1_c$	N.O.	10 s^{-1}	N.O.	10 s^{-1}	Not available
Dimerization rate constant [m]	k_b^{DIM} (D)	1—200 $nM^{-1}s^{-1}$	200 $nM^{-1}s^{-1}$	0.01—0.2 $\mu m^2 s^{-1}$	0.200 $\mu m^2 s^{-1}$	0.01-0.02 $\mu m^2 s^{-1}$ [19]; 0.05 $\mu m^2 s^{-1}$ [20]; 0.2 $\mu m^2 s^{-1}$ [21]
Dimer dissociation rate constant[m]	k_u^{DIM}	0.07–20 s^{-1}	1.1s^{-1}	0.07–20 s^{-1}	1.1s^{-1}	2 s^{-1} [21]; 1.24s^{-1} (no EGF bound), 0.738s^{-1} (1 EGF bound), and 0.271s^{-1} (2 EGF bound) [20]
Phosphorylation rate constant[n]	k_{KIN}	0.005—10 s^{-1}	0.289 s^{-1}	0.005—10 s^{-1}	0.289 s^{-1}	> 0.005 s^{-1} [1]; 0.0733 s^{-1} [2]; 0.2 s^{-1} [4]; > 0.075 s^{-1}/ 2 s^{-1} [depending on model in [3]]; 1s^{-1} [5]

Supplementary Table 1. Model Parameters. Parameters or molecular species were either measured ("this manuscript") or taken from the literature. We distinguish between experimental measurements (references in black) and estimates produced by previous modeling studies (references in red). Parameters were either fitted in a given interval or not optimized (N.O.). In Supplementary note 2 we provide a thorough explanation for the choice of each parameter.

Note that, in the model, reactions take place in three different compartments: EGFR binding to EGF occurs in the volume occupied by the medium (V_{MEDIUM}), EGFR dimerization occurs on the cell membrane (V_{MEMBRANE}), and EGFR binding to Cbl and Grb2 occurs in the cytoplasm (V_{cyt}). To avoid using different concentrations for the same species, we computed concentrations using the volume of the medium occupied by each cell V_{MEDIUM}, as shown in[6]. Notice that for this reason, the concentrations are not directly comparable to the experimental one: for the conversion, it will be enough to multiply for the proper volume. For example, the concentration of EGFR in the medium was computed from the concentration in the membrane as $[EGFR]_{\mathrm{MEDIUM}} = [EGFR]_{\mathrm{MEMBRANE}} \times V_{\mathrm{MEMBRANE}}/V_{\mathrm{MEDIUM}}$. In a typical experiment, we have in the order of 3-5x10^{6} cells growing in 3-5 ml of medium, which gives the volume per cell $V_{\mathrm{MEDIUM}} \approx 0.5\times10^{-6}$ ml. To estimate V_{cyt}, we approximated HeLa cells as spheres having a radius of 15 μm. We thus obtained $V_{\mathrm{cyt}} \approx 10^{-8}$ml. V_{MEMBRANE} was estimated as the surface of the cell x the height of EGFR, which is of the order of 10 nm, resulting in $V_{\mathrm{MEMBRANE}} \approx 10^{-11}$ ml. The rescaling affected also the constants with an explicit dependence on concentration. Also in this case, as shown above, rescaling took into account the ratio of the volumes. Thus, for all the parameters we provided values in typical units (to be directly compared to the one described in the literature) and the corresponding values scaled to the volume of the medium (used to build the model).

Relations linking equilibrium constants to the corresponding forward and backward rate constants are: $K = k_{\mathrm{u}}/k_{\mathrm{b}}$, $K1 = k1_{\mathrm{u}}/k1_{\mathrm{b}}$, $K_{\mathrm{e}} = k_{\mathrm{c}}/k_{\mathrm{o}}$, $K1_{\mathrm{e}} = k1_{\mathrm{c}}/k1_{\mathrm{o}}$, $K_{\mathrm{DIM}} = k_{\mathrm{u}}^{\mathrm{DIM}}/k_{\mathrm{b}}^{\mathrm{DIM}}$,

$K_{\mathrm{ph}} = k_{\mathrm{PTP}}/k_{\mathrm{KIN}}$, $K45 = ku45/kb45$, $K68 = ku68/kb68$, $Kcg = kucg/kbcg$. The parameters not listed can be computed using the definitions given above, e.g. $k_{\mathrm{u}} = k_{\mathrm{b}}\times K$.

a. Assuming that HeLa cells contain an average of $2.5\text{-}3\times10^{5}$ EGFRs (measured by saturation binding assay, see Methods) and that each cell is surrounded by 0.5×10^{-6} ml of medium.
b. We estimated the amount of Cbl available for EGF interaction by measuring the levels of Cbl phosphorylated by the EGFR at the maximal EGF concentration (see main text). This represents the maximal "active Cbl" fraction, i.e. able to recruit E2 ubiquitin-conjugating enzymes and to ubiquitinate the EGFR. Thus, we take this as a proxy for the maximal amount of Cbl actually available for binding to the EGFR (measured in Fig. 3b).
c. Measured in Fig. 3b.
d. In the MPM, we set the active EGFR fraction as an Hill function of EGF and optimized the parameters via a best-fit procedure; see Supplementary note 2 and Eq. (3).
e. Phosphatase rate constant was taken from the experimental measurements done at 2 min of EGF stimulation in[3] (Supplementary Table Pag. 8 herein); see also Supplementary note 2.
f. Previous measurements of Kd of Grb2 binding to phosphopeptide pY1068[7, 8, 9], or of Cbl TKB domain to phosphopeptide pY1045[10] are indicated. Dissociation rate constants for Grb2-pY1068 were also previously measured[8, 9] see also Supplementary note 2.
g. Measurements of Kd of binding between Cbl and Grb2 were not reported.
h. The localization factor f_{LOC} represents the concentration of a single molecule in the volume occupied by the dimer EGFR/Cbl (or by EGFR/Grb2 or by Cbl/Grb2); f_{LOC} is denoted as $[1]_{V_{\mathrm{R-Cbl}}}$ in Supplementary note 1, paragraph ``EGFR binding to Cbl and Grb2''.
i. See Supplementary note 2.
l. See Supplementary note 2. We assumed that the opening and closing of the extracellular domain of EGFR is at steady-state with respect to all other kinetics, both for free and liganded receptors. We thus fixed k_{c} and $k1_{\mathrm{c}}$ at a value greater than all the other parameters.
m. See Supplementary note 2. Experiments measure EGFR diffusion constant (D).
n. See Supplementary note 2.

Supplementary Table 2

Reaction type or species	Symbol	Optimization Range ($V=5x10^{-7}$ ml)	Value (from EAM)	Value (MPM-B)
Total concentration of EGFR	R_T	N.O.	0.83 nM	0.83 nM
Concentration of Cbl available for EGFR interaction	Cbl_T	N.O.	$1.5x10^{-2}$ nM	$1.5x10^{-2}$ nM
Total concentration of Grb2	$Grb2_T$	N.O.	3.3 nM	3.3 nM
Phosphatases rate constant	k_{PTP}	N.O.	0.016 s^{-1}	0.016 s^{-1}
Maximum phosphorylation rate constant	k_{KIN}^{MAX}	0.005—10 s^{-1}	0.0759 s^{-1}	0.0753 s^{-1}
Hill coefficient for phopshorylation curve	n_H	0.5-1.5 ng/ml	1.13	1.13
Half-maximum phosphorylation of EGFR	H	0.1-10 nM	4.6 nM	4.6 nM
Kd of of pY1068/pY1086 and Grb2 binding (and of pY1045 and Cbl)	$K45$ ($K68$)	0.2—150 nM	**0.2 nM**	0.19 nM
Kd of Cbl and Grb2 binding	Kcg	0.006—30 nM	**0.006 nM**	0.006 nM
Rate constant of dissociation of pY1068/pY1086 and Grb2 pY1045 and Cbl (and of pY1045 and Cbl)	$ku45$ ($ku68$)	0.001—40 s^{-1}	**0.001 s^{-1}**	0.0017 s^{-1}
Rate constant of dissociation of Cbl and Grb2	$kucg$	10^{-2}—10^{2} s^{-1}	**0.3 s^{-1}**	0.3 s^{-1}
Localization factor	f_{LOC}	10—10^{5} nM	**$2x10^{4}$ nM**	$2x10^{4}$ nM
Maximum ubiquitination	Ub_{MAX}	0.0005—0.05 nM	**0.0143 nM**	0.0143 nM
Maximum phosphorylation	$pYmax$	0—2.5 nM	2.22 nM	2.22 nM

Supplementary Table 2. **Parameters specific to the MPM-B model.** In the first column, the reaction/species used in the MPM-B model; in the second column, the symbols of the relative parameter. The third column reports the optimization range. In the fourth column, we report the parameters used for the MPM-B model in Fig. 4, Fig. 5, Supplementary Fig. 2, Supplementary Fig. 3b–c, Supplementary Fig. 4, and Supplementary Fig. 5. Notice that, with the exception of the parameters determining EGFR phosphorylation in the MPM-B (i.e, k_{KIN}^{MAX}, n_H, H, and pY_{max}), the values of the parameters are fixed by optimizing the EAM model (bold in the table). Finally, in the fifth column we report the parameters optimized on experimental data using the MPM-B model. Notice how the parameters fitted with the two models are very similar. For an explanation of each parameter, we refer to the legend of Supplementary Table 1.

Supplementary note 1 - EGFR models

In this section, we provide synopses of the EGFR.

The Multi-site Phosphorylation Model (MPM) describes EGFR phosphorylation and dephosphorylation using the ordinary differential equations (ODE) provided in Eq. (2). The mapping between the amount of EGF and the extent of kinase activity of EGFR is modeled with a Hill function, see Eq. (3). Phosphorylation was measured with Eq. (4). The ODE file (XPPAUT readable) that we used to produce Fig. 2d–e of the main text is available as Supplementary File 1.

The Multi-site Phosphorylation Model plus ubiquitination (MPM-B) is composed of all the reactions of the MPM supplemented with ubiquitination reactions. Cbl, Grb2 and EGFR binding are described by reactions given by Eqs. (5)–(8). In the MPM-B, the phosphorylation/dephosphorylation reactions are described by Eqs. (10), and phosphorylation was measured with Eq. (4). Ubiquitination was computed solving Eq. (15). MPM-B was used to produce Fig. 4, Fig. 5, Supplementary Fig. 2, Supplementary Fig. 3b–c, Supplementary Fig. 4, and Supplementary Fig. 5. Parameters are listed in Supplementary Table 2. The SBML file with MPM-B model reactions is available at biomodels.org (MPM-B: MODEL1505190001).

The early EGFR Activation Model (EAM) contains an explicit model of EGFR activation, phosphorylation and uniquitination: EGF binding to EGFR monomers, Eq. (19); opening and closing of the extracellular domain of EGFR, Eq. (22); EGF binding to dimeric forms of EGFR, Eq. (29); EGFR dimerization, Eqs. (24) and (30); EGFR phosphorylation/dephosphorylation, Eqs. (10). Phosphorylation was measured using Eq. (37). Finally, ubiquitination is dealt with similarly to the MPM-B, and thus measured with Eq. (39). EAM was used to produce Fig. 6b–d, Fig. 7a, Fig. 7c, Fig. 8a–c and Supplementary Fig. 11. The EAM file is available at biomodels.org (MODEL1505190000).

For all models, we used the conservation laws for the total number of EGFR as on the timescale of our system (2 minutes) protein synthesis and degradation do not play a relevant role. For EAM

also EGF is conserved, while for MPM and MPM-B, EGF is an independent variable. In Supplementary Table 1, we provide the values of the parameters present in the MPM-B and the EAM. We set the initial condition of each simulation by running the corresponding model up to the steady-state, in the absence of EGF stimulation. For simplicity, the initial condition of the simulations run to compute the sensitivity analysis and the curves with varying EGFR levels are prepared such that all species are unbound.

MPM-B and EAM models were written using the standard systems biology markup language [SBML[22]; www.sbml.org]. We used the MATLAB toolboxes SBtoolbox2[23] (www.sbtoolbox2.org) to translate the SBML version of the model into the ordinary differential equation (ODE) format, readable by XPPAUT (http://www.math.pitt.edu/~bard/xpp/xpp.html).

Minimal model for EGFR phosphorylation (MPM)

EGFR phosphorylation as a multisite chain of events. Here we describe the model for EGFR phosphorylation and dephosphorylation. To build an EGFR model that accounted for all the potential combinations of phosphotyrosines (pYs), in principle we should follow the phosphorylation state of each Tyr, and thus for N Tyr we would have 2^N possible EGFR states (e.g., for EGFR-WT we have 2^9=512). To simplify matters, we decided to group together species sharing the same number of pYs, based on the assumption (supported by experimental data in Fig. 1c–d) that the Tyrosines are phosphorylated independently from each other. Unphosphorylated receptors were then denoted as R_0, the sum of all EGFR species with 1 pY as R_1, the sum of EGFR species with 2 pYs as R_2, and so on, irrespective of the identity of the pYs. After this simplification, an EGFR with N phosphorylatable Tyr only has N+1 phosphorylation states, e.g., 10 in the case of EGFR-WT. A limitation of this approach is that the model will only give the probability that a specific Tyr is phosphorylated. The phosphorylation/dephosphorylation kinetics determines the transitions among these R_α states. In terms of the new variables, we can model phosphorylation

(dephosphorylation) as the transition to a state in which the number of phosphate groups is increased (decreased) by one:

$$R_{\alpha} \underset{\tilde{k}_{\mathrm{PTP}}(\alpha)}{\overset{\tilde{k}_{\mathrm{KIN}}(\alpha)}{\rightleftarrows}} R_{\alpha+1}, \qquad \alpha=0, ..., \mathrm{N}, \tag{1}$$

where R_{α} denotes an EGFR with α phosphate groups, while $\tilde{k}_{\mathrm{KIN}}(\alpha)$ and $\tilde{k}_{\mathrm{PTP}}(\alpha)$ denote the rate constants for phosphorylation and dephosphorylation of R_{α}, respectively. The chemical reactions of Eq. (1) translate into the following ODEs

$$\begin{aligned} &\mathrm{dR}_0/\mathrm{dt} = -\tilde{k}_{\mathrm{KIN}}(0)\mathrm{R}_0 + \tilde{k}_{\mathrm{PTP}}(1)R_1 \\ &\mathrm{dR}_{\alpha}/\mathrm{dt} = \tilde{k}_{\mathrm{KIN}}(\alpha-1)R_{\alpha-1} - \tilde{k}_{\mathrm{KIN}}(\alpha)\mathrm{R}_{\alpha} + \tilde{k}_{\mathrm{PTP}}(\alpha+1)R_{\alpha+1} - \tilde{k}_{\mathrm{PTP}}(\alpha)\mathrm{R}_{\alpha}, \qquad \alpha=1, ..., \mathrm{N}-1 \\ &\mathrm{dR}_N/\mathrm{dt} = \tilde{k}_{\mathrm{KIN}}(N-1)R_{N-1} - \tilde{k}_{\mathrm{PTP}}(N)\mathrm{R}_N, \end{aligned} \tag{2}$$

which correspond to multi-site phosphorylation [24, 25, 26, 27, 28].

Free-free and saturated-saturated regimes studied with the Gillespie method. In Fig. 2b, we show that two limiting regimes (free and saturated) can be identified for the reaction catalyzed by kinases, depending on the stability of the complex between the catalytic subunit of EGFR and its target Tyr. To simulate the reactions defined by Eq. (2), since we are dealing with a small number of molecules—one enzyme and a maximum of 9 substrates (Tyr) for EGFR-WT—we have to resort to stochastic simulations. We fixed $k_{\mathrm{cat}} = 10$ and $k_{\mathrm{off}} = 1$ and varied k_{on}. Fig. 2b shows the dependence of the macroscopic rate constant $\tilde{k}(\alpha)$ on the number of pYs ($\alpha = 0,...,8$ in the figure) in units of k_{KIN} (the basic rate constant that governs the addition of a phosphoryl group in the absence of competing Tyr). For $(k_{\mathrm{on}} + k_{\mathrm{off}})/k_{\mathrm{cat}} = 10^{-2}$ (blue dashed line), k_{cat} controls the rate-limiting step, a condition that we call the saturated-enzyme regime. For $(k_{\mathrm{on}} + k_{\mathrm{off}})/k_{\mathrm{cat}} = 10^4$ (black line), k_{on} controls the rate limiting step, a condition that we dubbed the free-enzyme regime. For intermediate values $(k_{\mathrm{on}} + k_{\mathrm{off}})/k_{\mathrm{cat}} = 10$ (red line), there is no single limiting step. Curves represent the average of 10^5 runs of the stochastic Gillespie algorithm [29] applied to the standard Michaelis-Menten reaction scheme depicted in Fig. 2a and mathematically described by Eq. (2).

In summary, the figure show that the free-enzyme regime corresponds to $\tilde{k}_{\mathrm{KIN}}(\alpha) = (N - \alpha)k_{\mathrm{KIN}}$ for kinases and $\tilde{k}_{\mathrm{PTP}}(\alpha) = \alpha k_{\mathrm{PTP}}$ for phosphatases, while the saturated-enzyme regime corresponds to $\tilde{k}_{\mathrm{KIN}}(\alpha) = k_{\mathrm{KIN}}$ for kinases and $\tilde{k}_{\mathrm{PTP}}(\alpha) = k_{\mathrm{PTP}}$ for phosphatases, where k_{KIN} and k_{PTP} denote the phosphorylation and dephosphorylation rate constants for a single Tyr. Since each regime may be applied independently to kinases and phosphatases, in principle we have four models of phosphorylation: the saturated-saturated, saturated-free, free-saturated and free-free where the first term always refers to kinases and the second to phosphatases. Only when the free-free regime is adopted (not shown), does the model reproduce faithfully the shape of the dose-response curves, as well as the ratio of EGFR-WT and EGFR-3Y+ phosphorylation (Fig. 2d–e). Thus, in our model $\tilde{k}_{\mathrm{KIN}}(\alpha) = (N - \alpha)k_{\mathrm{KIN}}$ and $\tilde{k}_{\mathrm{PTP}}(\alpha) = \alpha k_{\mathrm{PTP}}$.

Since EGFR follows the free-enzyme regime for both phosphorylation and dephosphorylation, the phosphorylation of each Tyr is independent of that of the other Tyr. As a consequence, modeling EGFR with $N = 9$ or $N = 3$ does not make any difference, because only Y1045, Y1068 and Y1086 are relevant for EGFR ubiquitination.

Mapping how EGFR activity depends on EGF concentration. We then needed to define how EGF modifies the extent of EGFR activity, i.e. to define k_{KIN} as a function of EGF. We disregarded altogether the mechanisms of EGFR activation and followed an empirical approach: we assumed that k_{KIN} is a function of EGF as described by a Hill function:

$$k_{\mathrm{KIN}}(L) = k_{\mathrm{KIN}}^{\mathrm{MAX}} \frac{L^{n_{\mathrm{H}}}}{H^{n_{\mathrm{H}}} + L^{n_{\mathrm{H}}}}. \qquad (3)$$

In Eq. (3), L stands for the EGF concentration, the Hill coefficient nH represents how steeply k_{KIN} increases with EGF, while H is the EGF concentration where k_{KIN} is half-maximal. Eq. (3) describes a sigmoid varying between 0 and $k_{\mathrm{KIN}}^{\mathrm{MAX}}$, which is the maximal kinetic activity of EGFRs. Conversely, we assume that k_{PTP} stays constant for EGFR in the plasma membrane in the first two minutes after EGF stimulus. With this mapping, the equations describing the transitions among the

phosphorylation states of EGFR are those described by Eq. (1) and (2), provided that k_{KIN} is substituted by $k_{\mathrm{KIN}}(L)$. In summary, the occupancy of each of the R_α, which denotes an EGFR with α phosphate groups, depends on the concentration of EGF and on the parameters n_{H}, H, $k_{\mathrm{KIN}}^{\mathrm{MAX}}$, and k_{PTP}, collectively represented by a vector **k**. For a given EGF concentration L, $R_\alpha(L, \mathbf{k}, t)$ represents the fraction of EGFR with α phosphate groups at time t.

Measuring phosphorylation in MPM. Since the C-terminal tail of EGFR, where the relevant Tyr are located, is predicted to be unstructured and very flexible, we assumed that the pan-pY antibody (4G10, Millipore) recognizes phosphorylated Tyr independently of EGFR conformation. - $R_\alpha(L, \mathbf{k}, t)$–which depends on time, EGF concentration L, and on the parameters n_{H}, H, $k_{\mathrm{KIN}}^{\mathrm{MAX}}$, and k_{PTP}–has a number of pYs equal to α, with $\alpha = 0, \dots, N$. The expression for the total level of EGFR phosphorylation that can be directly compared to the experimental data is:

$$pY(L,\mathbf{k},t) = \sum_{\alpha=1}^{N}\left[\alpha R_\alpha(L,\mathbf{k},t)\right] \tag{4}$$

EGFR binding to Cbl and Grb2. In this section, we discuss the mathematical formalization of EGFR ubiquitination, which depends on Cbl and Grb2 binding to the receptor. As summarized in Fig. 3a of the main text, Cbl binds EGFR at pY1045 and Grb2 binds at pY1068 or pY1086. Cbl and Grb2, bound or not bound to the EGFR, can also bind to each other. Thus, two kinds of trimers exist: one characterized by three bindings (a pY1045-bound Cbl binds a Grb2 molecule, which is bound to either pY1068 or pY1086), the other by two bindings (Cbl binds both EGFR and Grb2, which however does not bind directly to EGFR; or Grb2 binds both EGFR and Cbl, which does not bind EGFR; or EGFR binds both Cbl and Grb2, which do not bind each other). We consider also tetramers with one EGFR, one Cbl and two molecules of Grb2, of which one is directly bound to EGFR and the second is indirectly bound through the EGFR-bound Cbl. Since Cbl overexpression experiments suggested that Cbl concentration is negligible with respect to EGFR concentration (see

main text and Fig. 3b–c), we disregarded all complexes that are unlikely to occur: those with more than one Cbl moiety, i.e., tetramers with two Cbl molecules and pentamers.

Hereafter, we introduce the dynamics of Cbl and Grb2 binding for EGFR-3Y+. For brevity, we only list complexes formed by Cbl and Grb2 with a prototype EGFR that we identify with R. Later on, we introduce an explicit model of EGFR activation, EAM, with more EGFR species. Even for that model, the equations presented in the following are valid taking in mind that the corresponding complexes for the other EGFR conformations may be readily obtained by substituting R with any of the other 7 conformations introduced for the explicit EGFR activation model. The new variables are denoted using the following rules: superscripts identify Y1045, while subscripts identify the pair Y1068/Y1086; unphosphorylated Tyr lacks a "P" at the corresponding position; two proteins forming a complex are represented by their names separated by a colon, e.g., Cbl:Grb2. Given these rules, R^{P} represents a receptor where the only phosphorylated species is Y1045 (pY1045); R_{P} a receptor with either pY1068 or pY1086; $R_{2\mathrm{P}}$ a receptor with both pY1068 and pY1086; $R_{2\mathrm{P}}^{\mathrm{P}}$ has all three Tyr phosphorylated. R^{P} can bind Cbl ($R^{\mathrm{P:Cbl}}$), which can in turn bind Grb2 ($R^{\mathrm{P:Cbl:Grb2}}$). Likewise, R_{P} (and equivalently $R_{2\mathrm{P}}$) can form the complexes $R_{\mathrm{P:Grb2}}$ and $R_{\mathrm{P:Grb2:Cbl}}$. Finally, $R_{\mathrm{P}}^{\mathrm{P}}$ (and $R_{2\mathrm{P}}^{\mathrm{P}}$), besides all complexes already listed, can form the complexes $R_{\mathrm{P:Grb2}}^{\mathrm{P:Cbl}}$ (where Cbl and Grb2 are not directly bound), $R_{\mathrm{P:Grb2}}^{\mathrm{P:Cbl:Grb2}}$, and the fully bound trimer where Cbl and Grb2 are bound both to the receptor and to each other. We indicate this state as $R_{\mathrm{P}}^{\mathrm{P}}{:}\,Cbl{:}\,Grb2$, to distinguish it from $R_{\mathrm{P:Grb2}}^{\mathrm{P:Cbl}}$ that has only two bindings involved. As stated above, we disregarded the four configurations $R_{\mathrm{P:Grb2:Cbl}}^{\mathrm{P:Cbl}}$, $R_{2\mathrm{P:Grb2:Cbl}}^{\mathrm{P:Cbl}}$, $R_{\mathrm{P:Grb2:Cbl}}^{\mathrm{P:Cbl:Grb2}}$ and $R_{2\mathrm{P:Grb2:Cbl}}^{\mathrm{P:Cbl:Grb2}}$, because the chance of forming a complex with two Cbl molecules is negligible.

Having defined the new variables, we will now list all possible reactions including Cbl and Grb2. Note that despite the large number of reactions involved, they are all straightforward binding reactions. It is worth stressing however three peculiarities. First, if EGFR has both pY1068 and pY1086, the Grb2-binding rate doubles with respect to a form of EGFR having only pY1068 or

pY1086 (doubling the binding sites doubles the chance to bind). Second, binding reactions are independent of one another. Third, we introduced a cooperative mechanism as explained in the main text, Fig. 3a.

The list of reactions begins with Cbl and Grb2 binding to free receptors and to each other. To shorten the list, we used an additional notation, without changing the number of species. To identify the Y1045 site, we use the symbol Z that can take two values: 'empty' and 'P'. Where 'empty' stands for Y1045 and 'P' for pY1045. For the Y1068/Y1086 site, X can take the values 'empty', 'P' and '2P' (the latter two collectively represented as 'αP', with $\alpha = 1, 2$). The association and dissociation reactions are

$$\begin{aligned}
&R_X^{\mathrm{P}} + Cbl \underset{ku45}{\overset{kb45}{\rightleftarrows}} R_X^{\mathrm{P:Cbl}} \\
&R_{\alpha\mathrm{P}}^{Z} + Grb2 \underset{kb68}{\overset{\alpha kb68}{\rightleftarrows}} R_{\alpha\mathrm{P:Grb2}}^{Z} \\
&Cbl + Grb2 \underset{kbcg}{\overset{kbcg}{\rightleftarrows}} Cbl:Grb2 \\
&R_X^{\mathrm{P}} + Cbl:Grb2 \underset{ku45}{\overset{kb45}{\rightleftarrows}} R_X^{\mathrm{P:Cbl:Grb2}} \\
&R_{\alpha\mathrm{P}}^{Z} + Cbl:Grb2 \underset{kb68}{\overset{\alpha kb68}{\rightleftarrows}} R_{\alpha\mathrm{P:Grb2:Cbl}}^{Z}
\end{aligned} \tag{5}$$

where: $kb45$ ($ku45$) is the association (dissociation) rate constant for pY1045 and Cbl; $kb68$ ($ku68$) is the association (dissociation) rate constant for either pY1068 or pY1086; $kbcg$ ($kucg$) is the association (dissociation) rate constant for Cbl-Grb2 binding. The ODEs corresponding to these chemical reactions can easily be obtained using mass action. For examples, refer to chemical reactions and ODEs given by Eq. (1) and (2), respectively.

The reactions regarding the binding of a second Cbl or Grb2 moiety to receptors are

$$\begin{aligned}
&R_X^{\mathrm{P:Cbl}} + Grb2 \underset{kucg}{\overset{kbcg}{\rightleftarrows}} R_X^{\mathrm{P:Cbl:Grb2}} \\
&R_{\alpha\mathrm{P:Grb2}}^{Z} + Cbl \underset{kbcg}{\overset{kbcg}{\rightleftarrows}} R_{\alpha\mathrm{P:Grb2:Cbl}}^{Z} \\
&R_{\alpha\mathrm{P}}^{\mathrm{P:Cbl}} + Grb2 \underset{ku68}{\overset{\alpha kb68}{\rightleftarrows}} R_{\alpha\mathrm{P:Grb2}}^{\mathrm{P:Cbl}} \\
&R_{\alpha\mathrm{P:Grb2}}^{P} + Cbl \underset{ku45}{\overset{kb45}{\rightleftarrows}} R_{\alpha\mathrm{P:Grb2}}^{\mathrm{P:Cbl}},
\end{aligned} \tag{6}$$

and those for a third moiety

$$R^{\mathrm{P:Cbl:Grb2}}_{\alpha\mathrm{P}} + Grb2 \underset{ku68}{\overset{\alpha kb68}{\rightleftarrows}} R^{\mathrm{P:Cbl:Grb2}}_{\alpha\mathrm{P:Grb2}}$$
$$R^{\mathrm{P:Cbl}}_{\alpha\mathrm{P:Grb2}} + Grb2 \underset{kucg}{\overset{kbcg}{\rightleftarrows}} R^{\mathrm{P:Cbl:Grb2}}_{\alpha\mathrm{P:Grb2}} \tag{7}$$

Finally, the three reactions that characterize the cooperative hypothesis for ubiquitination are

$$R^{\mathrm{P}}_{\alpha\mathrm{P:Grb2:Cbl}} \underset{ku45}{\overset{kb45^*}{\rightleftarrows}} R^{\mathrm{P}}_{\alpha\mathrm{P}}:Cbl:Grb2$$
$$R^{\mathrm{P:Cbl:Grb2}}_{\alpha\mathrm{P}} \underset{kb68}{\overset{\alpha kb68^*}{\rightleftarrows}} R^{\mathrm{P}}_{\alpha\mathrm{P}}:Cbl:Grb2 \tag{8}$$
$$R^{\mathrm{P:Cbl}}_{\alpha\mathrm{P:Grb2}} \underset{kbcg}{\overset{kbcg^*}{\rightleftarrows}} R^{\mathrm{P}}_{\alpha\mathrm{P}}:Cbl:Grb2$$

which lead to the formation of only fully ubiquitinated EGFR complexes.

The binding reactions of EGFR, Cbl, and Grb2 can take place in different ways, for example the binding of Cbl to pY1045 of EGFR can follow a first-order kinetics as described in reactions (8), with rate kb45*, but also a second-order kinetics, as in (5) and (6), with rate kb45. A similar observation can be made for the other reactions described in (8). The binding is favored by a cooperative mechanism when the first-order reactions are favored compared to the second-order reactions, i.e. when the following condition is satisfied:

$$kb45^* \gg kb45 \times Cbl$$
$$kb68^* \gg kb68 \times Grb2 \tag{9}$$
$$kbcg^* \gg kbcg \times Grb2$$

(the latter also implies $kbcg^* \gg kbcg \times Cbl$). Equations (9) are easily guaranteed by the enforced-proximity induced by the first binding. To show it, we can think of the first-order reaction $R^{\mathrm{P}}_{\alpha\mathrm{P:Grb2:Cbl}} \xrightarrow{kb45^*} R^{\mathrm{P}}_{\alpha\mathrm{P}}:Cbl:Grb2$ as a second-order reaction, where pY1045 binds the only Cbl molecule bound to pY1068 (or pY1086) on the same EGFR. The concentration of this single Cbl molecule is the concentration of 1 molecule in the small volume occupied by EGFR and Cbl, which we denote as $[1]_{V_{\mathrm{R\text{-}Cbl}}}$. In such a scenario, we can assume that $kb45^* = kb45 \times [1]_{V_{\mathrm{R\text{-}Cbl}}}$. Estimating $V_{\mathrm{R\text{-}Cbl}}$ as a cube with side equal to the largest dimension of Cbl and EGFR, i.e. the height of EGFR,

we obtained $V_{\text{R-Cbl}} \approx (10nm)^3 = 10^{-21} l$ and a concentration of $[1]_{V_{\text{R-Cbl}}} = 1/(N_{\text{AV}} \times V_{\text{R-Cbl}}) \approx 10^6 n\text{M}$, which is several orders of magnitude higher than the typical concentrations of Cbl that we simulate (≈10^{-3} nM). The simulations shown in Fig. 4, Fig. 5, Fig. 6b, Fig. 7c, Fig. 8a–c of the main text satisfy Eqs. (9), a condition that we define as cooperativity. If we disregard the spatial localization, i.e., we consider the single molecule as distributed in the cytosol with volume $V_{\text{cyt}} \approx 10^{-8} ml$ and substitute $[1]_{V_{\text{R-Cbl}}}$ with $[1]_{V_{\text{cyt}}} \approx 10^{-4} n\text{M}$ (as in Fig. 5a–b of the main text), we have a non-cooperative mechanism.

Phosphorylation/dephosphorylation in the presence of Cbl/Grb2 binding. It is likely that the phosphate groups involved in Grb2 or Cbl binding to the EGFR cannot be dephosphorylated when occupied. Thus, the dephosphorylation kinetics of the EGFR needs to be modified slightly to take into account Cbl and Grb2 binding. For example, when Cbl is bound to pY1045, it seems natural to assume that pY1045 is inaccessible to phosphatases and cannot be dephosphorylated. Unfortunately, this constraint renders the phosphorylation kinetics dependent on the EGFR configuration and we cannot fully adopt the phosphorylation model where we do not distinguish between different Tyr. Otherwise, using the same example as before, we would have the spurious effect that a pY1045-bound Cbl protects also pY1068 from dephosphorylation. To avoid this inconsistency, we single out Y1045, while we still consider collectively Y1068 and Y1086. Rather then using variables R_0, R_1, R_2 and R_3, we thus use the already introduced variables R, R^{P}, R_{P}, $R_{2\text{P}}$, R_{P}^{P} and $R_{2\text{P}}^{\text{P}}$. The increase of variables is modest when considering all possible Cbl and Grb2 bound configurations, since we move from 22 to 26 configurations. Again, for brevity, we introduce the notations: X=empty, P, 2P, P:Grb2, P:Grb2:Cbl, 2P:Grb2, 2P:Grb2:Cbl; Z=empty, P, P:Cbl, P:Cbl:Grb2; W=Grb2, Grb2:Cbl. Finally, the reactions for phosphorylation and dephosphorylation are

$$
\begin{aligned}
&R_X \underset{k_{\mathrm{PTP}}}{\overset{k_{\mathrm{KIN}}}{\rightleftarrows}} R_X^{\mathrm{P}} \\
&R^Z \underset{k_{\mathrm{PTP}}}{\overset{2k_{\mathrm{KIN}}}{\rightleftarrows}} R_{\mathrm{P}}^Z \\
&R_{\mathrm{P}}^Z \underset{2k_{\mathrm{PTP}}}{\overset{k_{\mathrm{KIN}}}{\rightleftarrows}} R_{2\mathrm{P}}^Z \\
&R_{\mathrm{P}:W}^Z \underset{k_{\mathrm{PTP}}}{\overset{k_{\mathrm{KIN}}}{\rightleftarrows}} R_{2\mathrm{P}:W}^Z
\end{aligned}
\tag{10}
$$

Measuring phosphorylation and ubiquitination in the presence of Cbl and Grb2. Total phosphorylation and ubiquitination are obtained by summing up the relevant species. For brevity, we defined 1PTyr, 2PTyr and 3PTyr as the sum of all EGFR species having 1, 2 and 3 phosphate groups, respectively:

$$
\begin{aligned}
1\mathrm{PTyr} &= R^{\mathrm{P}} + R^{\mathrm{P:Cbl}} + R^{\mathrm{P:Cbl:Grb2}} + R_{\mathrm{P}} + R_{\mathrm{P:Grb2}} + R_{\mathrm{P:Grb2:Cbl}} \\
2\mathrm{PTyr} &= \left(R_{2\mathrm{P}} + R_{2\mathrm{P:Grb2}} + R_{2\mathrm{P:Grb2:Cbl}} + R_{\mathrm{P}}^{\mathrm{P}} + R_{\mathrm{P}}^{\mathrm{P:Cbl}} + R_{\mathrm{P}}^{\mathrm{P:Cbl:Grb2}} + \right. \\
&\qquad \left. R_{\mathrm{P:Grb2}}^{\mathrm{P}} + R_{\mathrm{P:Grb2:Cbl}}^{\mathrm{P}} + R_{\mathrm{P:Grb2}}^{\mathrm{P:Cbl}} + R_{\mathrm{P:Grb2}}^{\mathrm{P:Cbl:Grb2}} + R_{\mathrm{P}}^{\mathrm{P}} : Cbl : Grb2\right) \\
3\mathrm{PTyr} &= \left(R_{2\mathrm{P}}^{\mathrm{P}} + R_{2\mathrm{P}}^{\mathrm{P:Cbl}} + R_{2\mathrm{P}}^{\mathrm{P:Cbl:Grb2}} + R_{2\mathrm{P:Grb2}}^{\mathrm{P}} + R_{2\mathrm{P:Grb2:Cbl}}^{\mathrm{P}} \right. \\
&\qquad \left. + R_{2\mathrm{P:Grb2}}^{\mathrm{P:Cbl}} + R_{2\mathrm{P:Grb2}}^{\mathrm{P:Cbl:Grb2}} + R_{2\mathrm{P}}^{\mathrm{P}} : Cbl : Grb2\right)
\end{aligned}
\tag{11}
$$

The total EGFR Tyr phosphorylation is expressed as

$$
\mathrm{PTyr} = 1\mathrm{PTyr} + 2 \times 2\mathrm{PTyr} + 3 \times 3\mathrm{PTyr} \tag{12}
$$

To compute total ubiquitination, the relevant species are the fully bound trimer $R_P^P: Cbl: Grb2$ and the complexes $R_{\mathrm{X}}^{\mathrm{P:Cbl}}$, $R_{\mathrm{X}}^{\mathrm{P:Cbl:Grb2}}$, $R_{\alpha\mathrm{P:Grb2}}^{\mathrm{P:Cbl}}$ and $R_{\alpha\mathrm{P:Grb2}}^{\mathrm{P:Cbl:Grb2}}$ where Cbl is only singly bound to the receptor. For brevity, again, we introduce two intermediate species:

$$
\begin{aligned}
Ub_{\mathrm{FULL}} &= \sum_{\alpha=1,2} \left(R_{\alpha\mathrm{P}}^{\mathrm{P}} : Cbl : Grb2\right) \\
Ub_{45} &= \sum_{\alpha=1,2} \left(R_{\alpha\mathrm{P}}^{\mathrm{P:Cbl}} + R_{\alpha\mathrm{P:Grb2}}^{\mathrm{P:Cbl}} + R_{\alpha\mathrm{P}}^{\mathrm{P:Cbl:Grb2}} + R_{\alpha\mathrm{P:Grb2}}^{\mathrm{P:Cbl:Grb2}}\right),
\end{aligned}
\tag{13}
$$

The ubiquitination then equals

$$
Ub = Ub_{\mathrm{FULL}} + Ub_{45} \tag{14}
$$

Eqs (14) show, in fact, that Ub_{FULL} and Ub_{45} are Cbl-bound EGFR species rather than true ubiquitinated EGFRs. To be precise, they should be regarded as ubiquitination *probabilities*. To

avoid introducing *ad-hoc* sources of sigmoidicity, we modeled actual ubiquitination as a completely first-order process, $dR_{\mathrm{Ub}}/dt = k_{\mathrm{ub}}Ub - k_{\mathrm{dub}}R_{\mathrm{Ub}}$, which at steady state becomes $R_{\mathrm{Ub}} = k_{\mathrm{ub}}Ub/k_{\mathrm{dub}}$ so that, eventually, the normalized Ub is computed as

$$\frac{R_{\mathrm{Ub}}}{R_{\mathrm{Ub}}^{\mathrm{MAX}}} = \frac{Ub}{Ub_{\mathrm{MAX}}}, \qquad (15)$$

where Ub_{MAX} is the maximum Ub value in the simulations. Note that we did not allow for saturation of receptor ubiquitination, because we assumed that EGFR can be ubiquitinated at multiple sites.

We used Eqs. (12) and (14) [or (15) at steady state that, for normalized ubiquitination, is equivalent to (14)] to compute the curves shown in Fig. 4, Fig. 5, Supplementary Fig. 3, Supplementary Fig. 4b–c, Supplementary Fig. 5 and Supplementary Fig. 6.

Explicit EGFR activation models (EAM)

Definition of EGFR model species. As mentioned above, EGFR has a total of 2^N possible phosphorylation states, where N=number of phosphorylatable Tyr. Since the receptor can be either closed or open, liganded or unliganded, each EGFR monomer can have 2^Nx2x2 states, i.e., 2,048 for EGFR-WT. Considering that extended (open) EGFRs can form dimers, we end up with a total of $(2\text{x}2^N)^2$ possible dimer states, i.e., 1,048,576 for EGFR-WT. Since cells contain in the order of 10^4 – 10^5 (10^5 in HeLa cells used in this study) EGFR molecules, most states will be empty, making it unnecessary to follow each of the possible receptor configurations individually. Using the simplification explained in ``EGFR phosphorylation as a multisite chain of events'', we can reduce the number of monomer states to N+1 (10 for N=9, as in EGFR-WT, and 4 for N=3, as in EGFR-3Y+), which become (N+1)x2x2 when we include EGF binding and conformational changes (open/close) of the EGFR extracellular domain (40 and 16 for EGFR-WT and EGFR-3Y+, respectively). For dimers, we reduced the number of states to $(2\text{x}(N+1))^2$ states (400 for EGFR-WT

instead of 1,048,576; 64 for EGFR-3Y+ instead of 256). To further reduce the number of receptor states to consider, in ``Definition of new dimeric species'' we propose an additional simplification for dimer species, which reduces their number from $(2\mathrm{x}(\mathrm{N}+1))^2$ to 4x(N+1). In summary, we only need to consider 40 monomeric and 40 dimeric states for EGFR-WT, and 16 monomeric and 16 dimeric states for EGFR-3Y+. Again, the model will only give the probability that an EGFR is phosphorylated. Taking into consideration these changes, we have detailed below the variables used for the EAM, which is schematically represented in Fig. 6a of the main text.

To account for all EGFR states, we denoted the open form of EGFR as R_α and its closed form as Rc_α, where $\alpha = 0, \dots, N$ (N is the total number of tyrosines and α that of pYs). Upon binding to EGF (henceforth denoted as L, for ligand), R_α and Rc_α become RL_α and RcL_α, respectively. Possible dimer forms are $R_\alpha : R_\beta$, $RL_\alpha : RL_\beta$, and $R_\alpha : RL_\beta$, where $\alpha, \beta = 0, \dots, N$. Here, and in the following model, we do not distinguish between the position of monomers in the dimer, i.e., there is no 'left' or 'right' distinction. Thus, $R_\alpha : R_\beta$ is equivalent to $R_\beta : R_\alpha$, $RL_\alpha : RL_\beta$ is equivalent to $RL_\beta : RL_\alpha$, and $R_\alpha : RL_\beta$ is equivalent to $RL_\beta : R_\alpha$. While in general $R_\alpha : RL_\beta$ and $R_\beta : RL_\alpha$ are two distinct species because the EGF-bound receptor is distinguishable by the number of phosphate groups attached, when $\alpha = \beta$ they represent the same species. To avoid duplication, we use the convention that the left index is always smaller than or equal to the right index, and we put the ligand on the right. Thus, $R_\alpha : R_\beta$ only exists when $\alpha < \beta$, while the species $RL_\alpha : R_\beta$ does not exist. The reader should remember these conventions when reading the ODEs that refer to dimers.

We consider that the total number of both EGFR and EGF molecules are conserved, because we are studying early events of EGFR activation, 2 min after EGF addition, when protein degradation and synthesis are insignificant. Conservation laws are:

$$R_\mathrm{T} = \sum_\alpha \left(R_\alpha + RL_\alpha + Rc_\alpha + RcL_\alpha \right) + 2\sum_{\alpha \le \beta} \left(R_\alpha : R_\beta + RL_\alpha : RL_\beta \right) + 2\sum_{\alpha,\beta} R_\alpha : RL_\beta \tag{16}$$

$$L_\mathrm{T} = L + \sum_\alpha \left(RL_\alpha + RcL_\alpha \right) + 2\sum_{\alpha \le \beta} \left(RL_\alpha : RL_\beta \right) + \sum_{\alpha,\beta} \left(R_\alpha : RL_\beta \right) \tag{17}$$

Given these conservation relations, we will now introduce the ODEs of the model. So far, we enumerated 4(N+1) monomer species, $2^2(N+1)^2$ dimer species and two conservation relations. The rate of change of the species between these states is determined by the kinetics of ligand binding, receptor dimerization and phosphorylation. Since these dynamics do not affect each other (e.g., phosphorylation state of EGFR does not affect its ability to bind EGF), we wrote independent ODEs for each of them. The final ODEs, for each variable used in the simulations, take into account all the dynamics simultaneously.

Ligand binding to EGFR. Classically, EGF binding to EGFR is represented by the chemical reaction $EGFR + EGF \underset{k_u}{\overset{k_b}{\rightleftarrows}} EGFR:EGF$, which for the open EGFR species reads as

$$R_\alpha + L \underset{k_u}{\overset{k_b}{\rightleftarrows}} RL_\alpha, \qquad (18)$$

where k_b and k_u are the binding and unbinding rate constants of EGF-EGFR binding, respectively. Since we always use mass-action kinetics, the ODEs corresponding to reactions (18) are:

$$\begin{aligned} \frac{dRL_\alpha}{dt} &= k_b R_\alpha \cdot L - k_u RL_\alpha \\ \frac{dR_\alpha}{dt} &= -k_b R_\alpha \cdot L + k_u RL_\alpha \\ \frac{dL}{dt} &= -k_b R_\alpha \cdot L + k_u RL_\alpha . \end{aligned} \qquad (19)$$

At steady-state, the ratio of bound to unbound EGFR is determined by the dissociation constant $K \equiv k_u / k_b = [EGFR][EGF]/[EGFR:EGF]$. Data obtained from the EGFR structure [30] show that the EGF-EGFR binding surface differs for the open and closed conformations. We thus defined two sets of EGF binding rate constants: the already introduced k_b and k_u ($K = k_u/k_b$) for open receptors, and $k1_b$ and $k1_u$ ($K1 = k1_u/k1_b$) for closed receptors. The chemical reactions and the ODEs for the closed form of EGFR can be readily obtained by substituting in Eqs. (18) and (19) Rc_α, RcL_α, $k1_b$ and $k1_u$ for R_α, RL_α, k_b and k_u, respectively.

In EGFR dimers, we assumed that each monomer binds to EGF independently. Thus, the EGF binding rate constant to $R_\alpha : R_\beta$ is twice that of the monomers. The same is true for the unbinding rate constant of $RL_\alpha : RL_\beta$. Using, Eq. (19) and the independence assumption, we obtained:

$$\begin{aligned}
\frac{dR_\alpha : R_\beta}{dt} &= -2k_\mathrm{b} R_\alpha : R_\beta \cdot L + k_\mathrm{u} R_\alpha : RL_\beta + k_\mathrm{u}\left(1-\delta_{\alpha\beta}\right) R_\beta : RL_\alpha, \quad \alpha \le \beta \\
\frac{dRL_\alpha : RL_\beta}{dt} &= -2k_\mathrm{u} RL_\alpha : RL_\beta + k_\mathrm{b} R_\alpha : RL_\beta : \times L + k_\mathrm{b}\left(1-\delta_{\alpha\beta}\right) R_\beta : RL_\alpha \times L, \quad \alpha \le \beta \qquad (20) \\
\frac{dR_\alpha : RL_\beta}{dt} &= -k_\mathrm{b} R_\alpha : RL_\beta \times L - k_\mathrm{u} R_\alpha : RL_\beta + k_\mathrm{b}\left(1+\delta_{\alpha\beta}\right) R_\alpha : R_\beta \times L + k_\mathrm{u}\left(1+\delta_{\alpha\beta}\right) RL_\alpha : RL_\beta
\end{aligned}$$

In Eqs. (20), the symbol $\delta_{\alpha\beta}$ is Kronecker's delta, which takes a value of 1 when $\alpha = \beta$, and 0 otherwise. Rather than explicitly solving an ODE for L, we computed the free ligand from the conservation relation Eq. (17). The same was done for species Rc_0, for which we took advantage of the conservation relation for R_T. We verified that Eqs. (19) and (20) satisfy the conservation relation Eq. (16).

Conformational changes of the extracellular domain of EGFR. We modeled the transition from the closed to open conformation of EGFR (and *vice versa*) as first-order transitions occurring at rates k_c and k_o for (c)losing and (o)pening, respectively:

$$Rc_\alpha \underset{k_\mathrm{c}}{\overset{k_\mathrm{o}}{\rightleftarrows}} R_\alpha \qquad (21)$$

the ratio of the two rates $K_\mathrm{e} = k_\mathrm{c}/k_\mathrm{o}$ determines, at steady-state, the ratio of closed to open EGFRs. In terms of ODEs, we obtained:

$$\begin{aligned}
\frac{dRc_\alpha}{dt} &= -k_\mathrm{o} Rc_\alpha + k_\mathrm{c} R_\alpha \\
\frac{dR_\alpha}{dt} &= k_\mathrm{o} Rc_\alpha - k_\mathrm{c} R_\alpha
\end{aligned} \qquad (22)$$

We assumed that the same transitions happen between RcL_α and RL_α, with rates $k1_\mathrm{c}$, $k1_\mathrm{o}$ and $K1_\mathrm{e} = k1_\mathrm{c}/k1_\mathrm{o}$, respectively. Microscopic reversibility imposes $K1_\mathrm{e} = (K \times K_\mathrm{e})/K1$.

EGFR homo-dimerization. Receptors in the open conformation (R_α and RL_α) can undergo dimerization. We let $k_\mathrm{b}^\mathrm{DIM}$ and $k_\mathrm{u}^\mathrm{DIM}$ be the binding and unbinding rate constants for dimerization, respectively, and let $K_\mathrm{DIM} = k_\mathrm{u}^\mathrm{DIM}/k_\mathrm{b}^\mathrm{DIM}$, where K_DIM is the dissociation constant for dimerization. The ODEs for dimer formation are:

$$\begin{aligned}
\frac{dR_\alpha : R_\beta}{dt} &= k_\mathrm{b}^\mathrm{DIM} R_\alpha \cdot R_\beta - k_\mathrm{u}^\mathrm{DIM} R_\alpha : R_\beta, \quad \alpha \le \beta \\
\frac{dRL_\alpha : RL_\beta}{dt} &= k_\mathrm{b}^\mathrm{DIM} RL_\alpha \cdot RL_\beta - k_\mathrm{u}^\mathrm{DIM} RL_\alpha : RL_\beta, \quad \alpha \le \beta \\
\frac{dR_\alpha : RL_\beta}{dt} &= k_\mathrm{b}^\mathrm{DIM} R_\alpha \cdot RL_\beta - k_\mathrm{u}^\mathrm{DIM} R_\alpha : RL_\beta
\end{aligned} \tag{23}$$

The corresponding ODEs for monomers:

$$\begin{aligned}
\frac{dR_\alpha}{dt} &= -k_\mathrm{b}^\mathrm{DIM} R_\alpha \sum_\beta \left[\left(1+\delta_{\alpha\beta}\right) R_\beta + RL_\beta \right] + k_\mathrm{u}^\mathrm{DIM} \sum_\beta \left[\left(1+\delta_{\alpha\beta}\right) R_\alpha : R_\beta + R_\alpha : RL_\beta \right] \\
\frac{dRL_\alpha}{dt} &= -k_\mathrm{b}^\mathrm{DIM} RL_\alpha \sum_\beta \left[R_\beta + \left(1+\delta_{\alpha\beta}\right) RL_\beta \right] + k_\mathrm{u}^\mathrm{DIM} \sum_\beta \left[\left(1+\delta_{\alpha\beta}\right) RL_\alpha : RL_\beta + RL_\alpha : R_\beta \right]
\end{aligned} \tag{24}$$

For functions satisfying $f(x_\alpha, x_\beta) = f(x_\beta, x_\alpha)$, the relation

$$2 \sum_{\alpha \le \beta} f(x_\alpha, x_\beta) = \sum_{\alpha,\beta} \left(1+\delta_{\alpha\beta}\right) f(x_\alpha, x_\beta) \tag{25}$$

holds. Using Eq. (25), we verified that dimerization reactions conserve total receptor mass Eq. (16), by summing Eqs. (23) and (24).

Definition of new dimeric species. Despite the change of variables described so far, for N=9 we still have 400 dimer species, whose equations can be written simply by adding Eqs. (20) and (23). A second critical simplification comes by focusing on single receptors only, regardless of whether

they are in a dimeric or monomeric configuration. Rather than following explicit dimers, we considered single molecules that are bound to a cognate EGFR. This class is denoted by a ‘*D*’, followed by an ‘*a*’ or an ‘*i*’ depending on whether the partner EGFR has an EGF bound or not, respectively (‘*a*’ stands for 'active', because phosphorylatable, ‘*i*’ for inactive because unphosphorylatable). If the receptor has an EGF bound, we also added a letter ‘L’, for ligand. With this simplification, for EGFR with N=9 Tyr, instead of following 400 dimeric species, we only needed to consider 40 EGFR dimer states, namely Di_α, Da_α, DiL_α, and DaL_α, where $\alpha = 0,...,9$. We have a factor of 2 increase from the 20 monomeric species because we distinguish whether their EGFR partner has an EGF moiety or not.

The remapping of the species follows the rules:

$$\begin{aligned}
Da_\alpha &= \sum_\beta R_\alpha : RL_\beta \\
Di_\alpha &= \sum_{\beta>\alpha} R_\alpha : R_\beta + \sum_{\beta<\alpha} R_\beta : R_\alpha + 2R_\alpha : R_\alpha \\
DaL_\alpha &= \sum_{\beta>\alpha} RL_\alpha : RL_\beta + \sum_{\beta<\alpha} RL_\beta : RL_\alpha + 2RL_\alpha : RL_\alpha \\
DiL_\alpha &= \sum_\beta R_\beta : RL_\alpha
\end{aligned} \tag{26}$$

Da_α represents EGFRs without EGF, dimerized with a receptor having an EGF bound; Di_α represents one of the moieties of a dimer where both receptors have no EGF bound; DaL_α represents one of the moieties of a dimer where both receptors have EGF bound; DiL_α represents EGFRs with EGF, dimerized with a receptor not having an EGF bound

Using Eqs (26), the conservation law for receptors, Eq. (16), now reads as

$$R_T = \sum_{\alpha=1}^{N} \left(R_\alpha + RL_\alpha + Rc_\alpha + RcL_\alpha + Da_\alpha + Di_\alpha + DaL_\alpha + DiL_\alpha \right) \tag{27}$$

In the following sections, we rewrite the rates for ligand binding and dimerization using these new variables.

EGF binding to collective species. To obtain the ODEs of variables defined by Eqs. (26), we differentiated Eqs. (26) and substituted Eqs. (20). For example, for EGF binding to species Di_α, we have

$$\begin{aligned}
\frac{dDi_\alpha}{dt} &= \sum_{\beta>\alpha} \frac{dR_\alpha : R_\beta}{dt} + \sum_{\beta<\alpha} \frac{dR_\beta : R_\alpha}{dt} + 2\frac{dR_\alpha : R_\alpha}{dt} = \\
&\sum_{\beta>\alpha} \left(-2k_\mathrm{b} R_\alpha : R_\beta \cdot L + k_\mathrm{u} R_\alpha : RL_\beta + k_\mathrm{u} R_\beta : RL_\alpha\right) \\
&+\sum_{\beta<\alpha} \left(-2k_\mathrm{b} R_\beta : R_\alpha \cdot L + k_\mathrm{u} R_\beta : RL_\alpha + k_\mathrm{u} R_\alpha : RL_\beta\right) \\
&+2\left(-2k_\mathrm{b} R_\alpha : R_\alpha \cdot L + k_\mathrm{u} R_\alpha : RL_\alpha\right) \\
&= -2k_\mathrm{b} \left(\sum_{\beta>\alpha} R_\alpha : R_\beta + \sum_{\beta<\alpha} R_\beta : R_\alpha + 2R_\alpha : R_\alpha \right) \cdot L + \\
&+k_\mathrm{u} \left(\sum_{\beta>\alpha} R_\alpha : RL_\beta + \sum_{\beta<\alpha} R_\beta : RL_\alpha + \sum_{\beta>\alpha} R_\beta : RL_\alpha + \sum_{\beta<\alpha} R_\alpha : RL_\beta + 2R_\alpha : RL_\alpha \right) \\
&= -2k_\mathrm{b} Di_\alpha \cdot L + k_\mathrm{u} \left(\sum_{\beta} R_\alpha : RL_\beta + \sum_{\beta} R_\beta : RL_\alpha \right) \\
&= -2k_\mathrm{b} Di_\alpha + k_\mathrm{u} \left(Da_\alpha + DiL_\alpha \right).
\end{aligned} \tag{28}$$

The ODEs for the three remaining D species were derived using the same procedure as outlined for Di_α, giving the system of ODEs:

$$\begin{aligned}
\frac{dDa_\alpha}{dt} &= -k_\mathrm{b}\left(Da_\alpha - Di_\alpha\right) \times L + k_\mathrm{u}\left(DaL_\alpha - Da_\alpha\right) \\
\frac{dDi_\alpha}{dt} &= -2k_\mathrm{b} Di_\alpha \times L + k_\mathrm{u}\left(DiL_\alpha + Da_\alpha\right) \\
\frac{dDaL_\alpha}{dt} &= k_\mathrm{b}\left(Da_\alpha + DiL_\alpha\right) \times L - 2k_\mathrm{u} DaL_\alpha \\
\frac{dDiL_\alpha}{dt} &= -k_\mathrm{b}\left(DiL_\alpha - Di_\alpha\right) \times L + k_\mathrm{u}\left(DaL_\alpha - DiL_\alpha\right).
\end{aligned} \tag{29}$$

Note that Eqs. (29) show no remaining dependence on the original dimeric species.

Dimerization for collective species. Differentiating Eqs. (26) and using Eqs. (23) we can write:

$$\begin{aligned}
\frac{dDa_\alpha}{dt} &= k_{\mathrm{b}}^{\mathrm{DIM}} R_\alpha \sum_\beta RL_\beta - k_{\mathrm{u}}^{\mathrm{DIM}} Da_\alpha \\
\frac{dDi_\alpha}{dt} &= k_{\mathrm{b}}^{\mathrm{DIM}} R_\alpha \sum_\beta \left(1+\delta_{\alpha\beta}\right) R_\beta - k_{\mathrm{u}}^{\mathrm{DIM}} Di_\alpha \\
\frac{dDaL_\alpha}{dt} &= k_{\mathrm{b}}^{\mathrm{DIM}} RL_\alpha \sum_\beta \left(1+\delta_{\alpha\beta}\right) RL_\beta - k_{\mathrm{u}}^{\mathrm{DIM}} DaL_\alpha \\
\frac{dDiL_\alpha}{dt} &= k_{\mathrm{b}}^{\mathrm{DIM}} RL_\alpha \sum_\beta R_\beta - k_{\mathrm{u}}^{\mathrm{DIM}} DiL_\alpha .
\end{aligned} \tag{30}$$

In contrast to Eqs. (29), which only contain dimeric species, Eqs. (30) include also monomeric species. For example, the first equation tells us that R_α becomes Da_α by binding to any monomer that has an EGF bound. Note that, each dimerization reaction appears twice on the right hand side of Eqs. (30), because it changes the concentration of two EGFRs in conformation '*D*'. Nicely, these double contributions are automatically accounted for by the summations on the right hand side of the ODEs, as these ODEs satisfy the conservation law [Eq. (27)].

EGFR Phosphorylation for EAM species. In ``EGFR phosphorylation as a multisite chain of events'', we explained how we reduced the kinase and phosphatase activity to a linear chain of increasingly phosphorylated EGFR species, i.e., to a multi-site phosphorylation model. In ``Free-free and saturated-saturated regimes studied with the Gillespie method'', we showed that since EGFR follows the free-enzyme regime for both phosphorylation and dephosphorylation, the phosphorylation of each Tyr is independent of that of the other Tyr, which allows us to model a 9Y EGFR with only N=3 tyrosines. To obtain the phosphorylation/dephosphorylation ODEs for species *Da* with N phosphorylatable Tyr, we employ the MPM in the free-free regime, which here translates into the following ODEs:

$$\begin{gathered}
\frac{dDa_0}{dt} = -Nk_{\mathrm{KIN}} Da_0 + k_{\mathrm{PTP}} Da_1 \\
\frac{dDa_\alpha}{dt} = -\left(\alpha k_{\mathrm{PTP}} + \left(N-\alpha\right) k_{\mathrm{KIN}}\right) Da_\alpha + \left(\alpha+1\right) k_{\mathrm{PTP}} Da_{\alpha+1} + \left(N-\alpha+1\right) k_{\mathrm{KIN}} Da_{\alpha-1}, \quad \alpha = 1,\ldots,N \\
\frac{dDa_N}{dt} = k_{\mathrm{KIN}} Da_{N-1} - Nk_{\mathrm{PTP}} Da_N
\end{gathered} \tag{31}$$

To keep track of the three key Tyr, we solved numerically a model with N=3, which can be written explicitly as:

$$\begin{aligned}
\frac{dDa_0}{dt} &= -3k_{\mathrm{KIN}}Da_0 + k_{\mathrm{PTP}}Da_1 \\
\frac{dDa_1}{dt} &= -\left(k_{\mathrm{PTP}} + 2k_{\mathrm{KIN}}\right)Da_\alpha + 2k_{\mathrm{PTP}}Da_2 + 3k_{\mathrm{KIN}}Da_0 \\
\frac{dDa_2}{dt} &= -\left(2k_{\mathrm{PTP}} + k_{\mathrm{KIN}}\right)Da_2 + 3k_{\mathrm{PTP}}Da_3 + 2k_{\mathrm{KIN}}Da_1 \\
\frac{dDa_3}{dt} &= k_{\mathrm{KIN}}Da_2 - 3k_{\mathrm{PTP}}Da_3
\end{aligned} \tag{32}$$

DaL, the other 'active' species, follows the same equations and can be obtained simply by replacing Da with DaL. The four monomeric species R, RL, Rc, and RcL, and the dimeric species Di and DiL are inactive and thus have a negligible k_{KIN}, such that the equations reduce to dephosphorylation only:

$$\begin{aligned}
\frac{dR_0}{dt} &= k_{\mathrm{PTP}}R_1 \\
\frac{dR_1}{dt} &= -k_{\mathrm{PTP}}R_1 + 2k_{\mathrm{PTP}}R_2 \\
\frac{dR_2}{dt} &= -2k_{\mathrm{PTP}}R_2 + 3k_{\mathrm{PTP}}R_3 \\
\frac{dR_3}{dt} &= -3k_{\mathrm{PTP}}R_3
\end{aligned} \tag{33}$$

We obtained the ODEs for the dephosphorylation kinetics of RL, Rc, RcL, Di , and DiL by replacing R with these species in Eqs. (33).

EAM phosphorylation in the absence of Cbl and Grb2. This section extends the corresponding definitions given for the MPM-B model to the new model with explicit EGFR activation that contains a large number of species. We assumed that the anti-pY antibody recognizes pYs independently of EGFR conformation; thus, R_α will have α pYs as well as Rc_α, RL_α, etc. Note that each of these terms depends upon EGF concentration, time, and all the parameters of the model (which we collectively represent as $\mathbf{k}$). It is convenient to define

$$EGFR_{\alpha}(L,\mathbf{k},t) = \left(R_{\alpha} + RL_{\alpha} + Rc_{\alpha} + RcL_{\alpha} + Di_{\alpha} + Da_{\alpha} + DiL_{\alpha} + DaL_{\alpha}\right) \quad (34)$$

where we explicitly kept the ligand, time, and parameter dependence only on the left hand side of the equation. The overall phosphorylation level of the EGFR is finally given by

$$pY(L,\mathbf{k},t) = \sum_{\alpha=1}^{N}\left[\alpha \times EGFR_{\alpha}(L,\mathbf{k},t)\right], \quad (35)$$

where summation over α ranges over all phosphorylation levels. Given the EGF concentration (L), to compute the total phosphorylation at any time t, we ran a simulation of EAM until time t and then used Eq. (35) to compute the total phosphorylation of the EGFR.

EAM phosphorylation and ubiquitination in the presence of Cbl and Grb2. The ubiquitination in the EAM model is analogous to the ubiquitination of the single EGFR conformation of MPM-B model. The complexes for the EGFR conformations of EAM may be readily obtained by substituting R with any of the other 8 conformations introduced above:
$R, Rc, RL, RcL, Di, DiL, Da, DaL$.

To compute the phosphorylation of each of the species we consider that Cbl and Grb2 protect tyrosines from. The equations for the phosphorylations are analogous to Eq. (10), with the only exception that only the active species can be phosphorylated. To obtain the equations for the active species, we need to substitute Da and DaL for R in Eq. (10), while for the inactive forms, i.e., for R , Rc , RL , RcL , Di and DiL , we also have to set $k_{\mathrm{KIN}} = 0$ or, equivalently, remove the phosphorylation reactions altogether.

To compute the phosphorylation, for brevity of notation, we define a collective variable P_{T} as the set of all possible EGFR species: $P_{\mathrm{T}} = \{R, Rc, R, RL, Di, DiL, Da, DaL\}$. In other words, P_{T} is the set of all 8 EGFR species and $P \in P_{\mathrm{T}}$ means that P is any of the EGFR species. With this notation we

have, for example, $\sum_{P \in P_T} P = R + Rc + R + RL + Di + DiL + Da + DaL$. We also defined 1PTyr, 2PTyr and 3PTyr as the sum of all EGFR species having 1, 2 and 3 phosphate groups, respectively:

$$\begin{aligned}
1\mathrm{PTyr} &= \sum_{\mathrm{P}\in\mathrm{P_T}} \left(\mathrm{P^P} + \mathrm{P^{P:Cbl}} + \mathrm{P^{P:Cbl:Grb2}} + \mathrm{P_P} + \mathrm{P_{P:Grb2}} + \mathrm{P_{P:Grb2:Cbl}}\right) \\
2\mathrm{PTyr} &= \sum_{\mathrm{P}\in\mathrm{P_T}} \left(\mathrm{P_{2P}} + \mathrm{P_{2P:Grb2}} + \mathrm{P_{2P:Grb2:Cbl}} + \mathrm{P_P^P} + \mathrm{P_P^{P:Cbl}} + \mathrm{P_P^{P:Cbl:Grb2}} + \right. \\
&\qquad \left. \mathrm{P^P_{P:Grb2}} + \mathrm{P^P_{P:Grb2:Cbl}} + \mathrm{P^{P:Cbl}_{P:Grb2}} + \mathrm{P^{P:Cbl:Grb2}_{P:Grb2}} + \mathrm{P^P_P} : Cbl : Grb2\right) \\
3\mathrm{PTyr} &= \sum_{\mathrm{P}\in\mathrm{P_T}} \left(\mathrm{P^P_{2P}} + \mathrm{P^{P:Cbl}_{2P}} + \mathrm{P^{P:Cbl:Grb2}_{2P}} + \mathrm{P^P_{2P:Grb2}} + \mathrm{P^P_{2P:Grb2:Cbl}} \right. \\
&\qquad \left. + \mathrm{P^{P:Cbl}_{2P:Grb2}} + \mathrm{P^{P:Cbl:Grb2}_{2P:Grb2}} + \mathrm{P^P_{2P}} : Cbl : Grb2\right)
\end{aligned} \tag{36}$$

Finally, we defined total EGFR Tyr phosphorylation as

$$\mathrm{PTyr} = 1\mathrm{PTyr} + 2 \times 2\mathrm{PTyr} + 3 \times 3\mathrm{PTyr} \tag{37}$$

Letting again $\mathrm{P_T} = \{R, Rc, R, RL, Di, DiL, Da, DaL\}$, we define two species Ub_{FULL} and Ub_{45}, which represent the fraction of EGFR with a moiety of Cbl either doubly or singly bound to pTyr1045, respectively. The two species are:

$$\begin{aligned}
Ub_{\mathrm{Full}} &= \sum_{\mathrm{P}\in\mathrm{P_T}} \sum_{\alpha=1,2} \left(\mathrm{P^P_{\alpha P}} : Cbl : Grb2\right) \\
Ub_{45} &= \sum_{\mathrm{P}\in\mathrm{P_T}} \sum_{\alpha=1,2} \left(\mathrm{P^{P:Cbl}_{\alpha P}} + \mathrm{P^{P:Cbl}_{\alpha P:Grb2}} + \mathrm{P^{P:Cbl:Grb2}_{\alpha P}} + \mathrm{P^{P:Cbl:Grb2}_{\alpha P:Grb2}}\right),
\end{aligned} \tag{38}$$

where with $P \in P_T$ P can take any value of the set P_T in the summation. The ubiquitination then equals, again, $Ub = Ub_{\mathrm{FULL}} + Ub_{45}$ and the normalized ubiquitination is computed as

$$\frac{R_{\mathrm{Ub}}}{R_{\mathrm{Ub}}^{\mathrm{MAX}}} = \frac{Ub}{Ub_{MAX}}. \tag{39}$$

The same considerations made after Eq. (15) on ubiquination probabilities apply here.

We used Eqs. (37) and (39) to compute the curves shown in Fig. 6b–d, Fig. 7a, Fig. 7c, Fig. 8a–c and Supplementary Fig. 11.

Supplementary note 2 – Definition of the models' parameters

Identifiability analysis. Before fitting the model to the experimental data, we investigated the identifiability of EAM parameters according to the Profile Likelihood Estimation (PLE) technique[31, 32], as implemented in the modeling framework PottersWheel[33]. The PLE algorithm detects identifiable and non-identifiable parameters; the latters are further distinguished between 'structural' and 'practical' non-identifiable parameters. Structural identifiable models do not contain intrinsic dependency among parameters so that multiple parametrizations give rise to different observables. These models are not properly defined. Practical non-identifiability instead arises when the amount of experimental data actually available does not ensure the exact estimation of parameters values; in other words, there exists different parameters estimates that are equally probable. Practical non-identifiability is common in computational biology [see, for example[3]].

In the case of EAM, the PLE identifiability analysis classified all the EAM parameters as structurally identifiable. However, the amount of data available for the fit has been not enough to ensure also practical identifiability: all model parameters, with the exception of k_{b}, $k_{\mathrm{b}}^{\mathrm{DIM}}$ and Ub_{MAX}, have been classified as practically non-identifiable.

Parameter optimization tools. Parameters were optimized for the EAM model (Supplementary Table 1) and for the MPM-B model (Supplementary Table 2). To obtain the best fit, we manually sampled the parameter space in search for regions compatible with the experimental data. Eventually, we used the MATLAB toolboxes SBtoolbox2 in combination with SBPD[23] (www.sbtoolbox2.org), using a global optimization method based on simulated annealing followed by the local optimization method "SBsimplex". The parameters and the optimization constraints are listed in Supplementary Table 1.

Selection of the optimization range for the model parameters. Hereafter, we describe, reaction by reaction, our choice of parameter values (see Supplementary Table 1). The choice deserves some explanation because, on the one hand, there is a large consensus that EGFR activation requires EGF binding, rearrangement of the extracellular domain and dimerization. On the other hand, there is still intense research on the nature of high and low affinity receptors, on the dependence of EGF affinity on the EGFR dimeric state, on the existence of intermediate active EGFR steps before EGF binding, and so on [see, among the others[14, 17, 18, 20, 34, 35, 36, 37, 38, 39, 40, 41], for a recent review see[42]]. Thus, here we want clarify explicitly our choice among the available literature.

Mapping of EGF to EGFR kinase activity. For MPM and MPM-B, we disregarded all interactions leading to EGFR activation by describing the active fraction of EGFR with a Hill function (Eq. (3)), for any given EGF concentration. The best fit of the MPM-B model can be obtained by fitting all parameters of the model directly to the experimental data, for both phosphorylation and ubiquitination. We show the corresponding optimized values in Supplementary Table 2. The values are very similar to those obtained by the EAM for the same reactions. To simplify the presentation and for a closer comparison of the EAM and the MPM-B models, we have decided to use in the MPM-B the values optimized for the EAM and shown in Supplementary Table 1. We thus determined the best fit of MPM-B by only optimizing the parameters relevant for EGFR phosphorylation: n_H, H, and $k_{\mathrm{KIN}}^{\mathrm{MAX}}$ (given in Supplementary Table 1), which are absent in EAM, and the phosphorylation normalization constant pYmax. We fixed the dephosphorylation parameter k_{PTP} to $k_{\mathrm{PTP}} = 0.016\, s^{-1}$, in agreement with the value reported in[3] (Supplementary Table 1). We also imposed a 5X weight to the point at 1 ng/ml for the phosphorylation curve, to better capture the difference between the phosphorylation and ubiquitination curves. For the MPM parameters, we used the corresponding values obtained for the MPM-B model.

Rate constants for phosphorylation and dephosphorylation in the EAM model. We modeled phosphorylation/dephosphorylation as first-order reactions, characterized by the rate of addition (k_{KIN}) and subtraction (k_{PTP}) of one phosphate group by kinases and phosphatases, respectively. Our observation that EGFR kinases work in the free-enzyme regime, suggests that the Michaelis-Menten relevant parameter is the ratio $k_{\mathrm{cat}}/K_{\mathrm{M}}$.

In vitro measurements of the activity of soluble forms of the EGFR kinase are available; $k_{\mathrm{cat}}/K_{\mathrm{M}}$ has been estimated as $6\times10^{-4}\mathrm{s}^{-1}\mu\mathrm{M}^{-1}$ [43] and $2.7\times10^{-5}\mathrm{s}^{-1}\mu\mathrm{M}^{-1}$ [44]. It is, however, doubtful whether these estimates are relevant for the EGFR kinase activity *in vivo*. In [45], for example, the authors report a 15X variation of the EGFR kinase activity depending on whether EGFR sits on vesicles or in solution, with $k_{\mathrm{cat}}/K_{\mathrm{M}}$ equals to $1.6\times10^{-3}\mathrm{s}^{-1}\mu\mathrm{M}^{-1}$ and $1.1\times 10^{-4}\mathrm{s}^{-1}\mu\mathrm{M}^{-1}$, respectively. Since the enzymatic parameters measured in these settings are unlikely to reproduce the kinetics *in vivo* of the full EGFR embedded on the plasma membrane, we decided to leave ample margin to the optimization algorithm.

Mass spectrometry measurements of single EGFR tyrosines *in vivo*[1] show that tyrosine phosphorylation is maximal at around 2 minutes after EGF stimulus. Biochemical assays following kinetics of total EGFR phosphorylation or single tyrosine sites show maximal phosphorylation between 2 to 10 minutes depending on cell types[3]. Inhibition of the EGFR kinase at 2 or 10 minutes after EGFR stimulation, allowed for the estimation of the EGFR phosphorylation decay, which leads to the dephosphorylation rate constant (k_{PTP})[3]. These experimental evidences suggest a phosphorylation time scale (the inverse of k_{KIN}) of the order of tens of seconds. We use these qualitative results to set a (wide) range of optimization for the phosphorylation rate constant. We also fixed the dephosphorylation rate constant to the experimentally measured value for k_{PTP} at two min after EGF addiction reported in Kleiman *et al.* $k_{\mathrm{PTP}} = 0.016\ s^{-1}$ [3]. Finally, mass spectrometry data [46] suggest that the simplifying assumption of using a single parameter for the phosphorylation kinetics of all EGFR tyrosines is acceptable in first approximation.

Dissociation and rate constants for Cbl and Grb2 binding. *Cbl and Grb2 binding to EGFR.* Cbl and Grb2 bind to EGFR at phosphorylated tyrosine 1045 (pY1045) and 1068/1086 (pY1068/pY1086), via their TKB (tyrosin-kinase binding domain) and SH2 domains, respectively. There are reports of the dissociation constants for the binding of full length Grb2 to phosphopeptide pY1068 SH2 in solution[7, 8, 9], and of Cbl TKB to phosphopeptide pY1045[10]. These measurements vary in the range 0.1–0.7 μM. Measurements of the dissociation rate of Grb2 and pY-1068 were also reported[8, 9]. The dissociation rate between Cbl TKB and pY-1045 instead is missing. To reduce the number of model parameters, we used a single rate constant for both Cbl–pY1045 binding and for Grb2-pY1068/pY1086 binding. This choice and the obvious observation that the actual constants for Cbl and Grb2 binding to EGFRs *in vivo* might differ from the ones measured, suggested us to allow for a wider optimization range for both the dissociation constants $K45$ and $K68$ (0.01-7 μM) and the unbinding rates $ku45$ and $ku68$ (0.001-40 s^{-1}).

Cbl and Grb2 interaction. Grb2 SH3 domains bind the Cbl proline-rich region. Grb2 has two SH3 domains, one located in the N-ter and one in the C-ter of the protein. Cbl has three proline-rich motifs located in the middle of the protein after the RING domain [47]. There are no studies that report *in vitro* measurements of Kd between purified Grb2 and Cbl. Instead, many studies focused on the binding between Grb2 and Sos-1 (which also contains proline-rich domains). Measurements in solution of SH3 domains of Grb2 with proline-rich region of Sos-1 vary a lot, in the range 1 nM–120 μM. This variability may depend, at least in part, on whether full-length Grb2[7, 8, 11] or isolated SH3 domains[9, 13] were used. In any case, all these studies suggested that binding of both N-ter and C-ter SH3 domains of Grb2 to multiple proline-rich sequences should decrease the Kd to the nM range [see also[48, 49]]. To be noted, Cbl has three proline-rich motifs that may contribute to the interaction with Grb2. In addition, early *in vivo* studies[50, 51], found that Cbl binds constitutively to Grb2 suggesting that Cbl–Grb2 binding may be stable. We thus set a wider optimization range for the dissociation constant Kcg, which represents the affinity of Cbl for Grb2.

We found no results for the dissociation rate of Cbl and Grb2, and therefore we leave an ample range of variation to the optimization algorithm for the corresponding parameter.

Rate constants for EGF binding and of opening-closing of the extracellular domain of the EAM model. The EGFR Activation Model (EAM), includes explicitly the known activation steps upon EGF stimulation. In particular EGF binding, rearrangement of the extracellular domain and dimerization are explicitly considered.

There is mounting evidence that the EGF affinity for EGFR depends both on the dimeric state of the receptors and on whether a dimer already has an EGF moiety bound[14, 20, 37]. Experiments that consider a simple binding between EGF and EGFR report a forward binding rate of in the range of $1{\times}10^{-3}\mathrm{s}^{-1}\mathrm{nM}^{-1}$ [15, 16]. Conversely, experiments that distinguish the binding to monomers and to preformed dimers detect slow-rate reaction for EGF binding in the range of $10^{-3}\mathrm{s}^{-1}\mathrm{nM}^{-1}$, and two types of fast-binding for EGF-molecules with rates $0.2\text{–}\ 0.4\ \mathrm{nM}^{-1}\mathrm{s}^{-1}$ for binding to empty dimers, and 2 $\mathrm{nM}^{-1}\mathrm{s}^{-1}$ for binding pre-bound dimers[18].

Reports that use equilibrium binding equations report two EGFRs subpopulations, distinguished by low and a high affinity for EGF. Without entering into the nature of these two populations (which is not the aim of the present study), we derived the corresponding dissociation constants that lie in the range of 0.2 nM–20 nM[6, 14, 15, 16, 17] and 2.9 nM–600 nM[14, 17], respectively. Since the main focus of this manuscript is not on EGFR activation *per se*, rather on how phosphorylated EGFRs become ubiquitinated, we took the simplified view that it is the extended (open) EGFR in dimers to have a high affinity for EGF, and set the dissociation constant of this reaction *K* as varying in the range 0.1–20 nM. The corresponding forward rate constant, which we denote k_b, is taken from the literature [18] as varying in the range $0.02\text{–}20\ \mathrm{nM}^{-1}\mathrm{s}^{-1}$, while the relation $k_\mathrm{u} = k_\mathrm{b}{\times}K$ assigns a value to the backward rate constant. To avoid that every EGFR conformation has a different affinity for EGF, we assumed that also monomeric extended receptors bind EGF with high affinity. Finally, we assume that the low affinity EGFR corresponds to the closed monomeric conformations, and

that they are characterized by a dissociation constant $K1 > 15nM$ (essentially we only require that the low affinity Kd is higher than the high affinity Kd and we set it at 50 nM), by a lower forward rate constant $k1_{\mathrm{b}} < k_{\mathrm{b}}$ and a higher backward rate constant $k1_{\mathrm{u}} > k_{\mathrm{u}}$.

We could not find measurements of the rate constants for the opening (k_{o}) and closing (k_{c}) of the extracellular domain of EGFR. In the model we set these parameters in such a way they do not directly affect the affinity of EGF for the receptors, which happens if the kinetics of opening and closing is orders of magnitude faster than EGF binding and EGFR dimerization. In this scenario, any value larger than the other rate constants will have the same effect on the activation of EGFR. Their ratio (K_e) instead determines the fraction of EGFR available for dimerization and could be estimated (together with the dimerization constant) by the fraction of EGFR in dimers, as we discuss in the next section.

Rate constants for EGFR dimerization. Direct measurements of EGFR diffusion and of EGF and EGFR dissociations showed a complex scenario. Diffusivity of EGFR dimers is lower compared to the diffusivity of monomers, probably because active dimers build signaling complexes that interact with other intracellular elements [19]. The stability of EGFR dimers also depends on the interaction of the dimer with intracellular molecules and of the binding of EGF. The diffusion coefficients reported for the rapidly diffusing species vary within an order of magnitude: $0.01\text{–}0.02\ \mu\mathrm{m}^2\ \mathrm{s}^{-1}$ [19]; $0.05\ \mu\mathrm{m}^2\ \mathrm{s}^{-1}$ [20]; $0.2\ \mu\mathrm{m}^2\ \mathrm{s}^{-1}$ [21]. We can translate the diffusion constant to the rate constant $k_{\mathrm{b}}^{\mathrm{DIM}}$ by assuming that dimerization is diffusion limited and, consequently, using the theoretical expression $k_{\mathrm{b}}^{\mathrm{DIM}} = 2\pi D/\ln{(b/r)}$ [52, 53]. We obtained $k_{\mathrm{b}}^{\mathrm{DIM}} \cong 2.7\times 10^{-2} \mu\mathrm{m}^2\mathrm{s}^{-1}$ setting the diffusion coefficient D for EGFR particles to $D \approx 10^{-10}\ \mathrm{cm}^2\ \mathrm{s}^{-1}$ [19], their radius r to $r \approx 5\text{–}7$ nm (measured using the pdb ID 3I2T of the extracellular domain), and computing b, which is half the mean distance between receptors on a membrane, using the surface area A (we estimate a HeLa radius in the order of $15\mu m$ and assume cells to be spherical) and the number of surface EGFR Γ_T

(of the order of 3–5×10^5) with the formula $b = \sqrt{A/(\pi\Gamma_T)}$. It is useful to transform the two dimensional rate constant k_b^{DIM} into a three dimensional one, by assuming that the third dimension is defined by the height of the EGFR, i.e. 10 nm. In the volume defined by the medium, the association rate constant for dimerization corresponding to the diffusion coefficient reported by Kusumi et al. is $k_b^{DIM} \cong 10nM^{-1}s^{-1}$. Using all the estimates, we obtain the following range of optimization $k_b^{DIM} \cong$ 1-200$nM^{-1}s^{-1}$ for the model parameter that corresponds to a range for the diffusion coefficient D, which is the measured observable, of 0.01-0.2 $\mu m^2 s^{-1}$

There are various measurements of the backward dimerization rate constant (k_u^{DIM}), which lies in the range 0.27– 2 s^{-1} depending on the number of EGF bound and on the type of assay used[20, 21].

The observation that no more than 5% of EGFR is dimerized in the absence of EGF stimulus[12] imposes $K_{DIM} \times Ke^2 \approx 40 R_T 17$, where: the dimensionless equilibrium constant K_e determines the ratio of open/closed conformation EGFRs; K_{DIM} is the dissociation constant for the binding of two EGFR molecules; i.e., dimerization; R_T is the total concentration of EGFR moieties. Given $R_T \approx 1nM$ (see Table 1) and considering $K_{DIM} \approx 0.001 - 1nM$ (coming from estimates of k_b^{DIM} and k_u^{DIM}), we obtain $Ke \approx 20 - 30$.

Supplementary note 3 - Sensitivity analysis

Although many parameters used were either available or determined *ad hoc* for this study, at least in terms of order of their magnitude, their precise values were fixed by means of a best-fit procedure described in Supplementary note 2. We thus decided to assess the robustness of the results to parameter variation. To this end, we performed a robustness analysis of the model, imposing a 1% increase and decrease for each parameter, to detect how the changes affect the output of the model in terms of steepness and half-maximal [EGF] of the dose-response curve for both phosphorylation and ubiquitination.

For each experiment analyzed in the main text [wild type (WT, Fig. 4a and Fig. 6b), Cbl overexpression (CblOE) and down-regulation (Cbl70Z) (Fig. 4b)], we measured the sensitivity coefficients $\sigma_{y_i}(k_j) = \left|\partial \ln y_i / \ln k_j\right| = \left|\partial y_i / y_i / \partial k_j / k_j\right|$, where: 'i' identifies the observable y_i (either steepness n_H or half-maximal value for total phosphorylation $pY_{0.5}$ or total ubiquitination x_T) and 'j' the parameter k_j. In practice, we compute the sensitivity coefficient using the formula

$$\sigma_{y_i}^{X}(k_j) = \frac{\dfrac{\left|y_i(\mathbf{k}^*) - y_i(\mathbf{k})\right|}{y_i(\mathbf{k})}}{\dfrac{k_j^* - k_j}{k_j}}, \tag{40}$$

where $\mathbf{k}$ stands for a vector containing all the parameters of the model, and $\mathbf{k}^*$ stands for the same vector with k_j substituted by k_j^*. X (referring to either WT, CblOE or Cbl70Z), determines the experimental conditions corresponding to the computed sensitivity coefficient. Since we wanted to assess the sensitivity of both the WT and mutated cellular conditions, we measured

$$\sigma_{y_i}(k_j) = \sum_X \sigma_{y_i}^{X}(k_j) \tag{41}$$

In Fig. 6c-d, we show the largest sensitivity coefficients computed using Eq. (41) for parameters variation of 1%.

The steepness of both EGFR phosphorylation and ubiquitination were remarkably robust: all sensitivity coefficients were below 0.5, which implies that this property of the curve is mainly encoded in the network wiring and not in the specific values of the parameters, at least around the best fit parameters. Even for the position of the half-maximal value, all sensitivity coefficients were below 1, with the most sensitive parameter being the first-order rate constant of phosphoryl group addition by kinases k_{KIN} (σ=0.9). Finally, it should be noted that all the Cbl- and Grb2-binding parameters showed a σ below 0.3, which ensures that primarily the phosphorylation kinetics determines the position of the ubiquitination threshold.

Supplementary note 4 – Description of L834R EGFR mutant

EGFR mutations in lung cancer, including L834R (also referred as L858R depending on whether the signal peptide is included in the aa numbering), occur in the tyrosine kinase domain of EGFR and predict significant clinical responses to EGFR kinase inhibitors[54, 55, 56]. L834R was shown to increase EGFR phosphorylation and activation[54, 57], as a result of different factors, i.e. decreased K_{d} for EGF-EGFR binding ($K57$), decreased K_{d} of dimer formation (K_{DIM}[58, 59]) and increased *in vitro* kinase activity (k_{KIN}[44, 45]). In particular, despite its increased phosphorylation (also at Tyr 1045), L834R shows decreased binding to Cbl and reduced ubiquitination[57]. Although the underlying mechanisms remain to be elucidated and possibly involve an increased propensity of the mutant to heterodimerize with HER2 (thereby evading Cbl recruitment[57]), there are enough measured parameters that can be set to challenge the model and test whether it is able to predict mutant's behavior. In the simulations, we used the following parameters for the mutant (compared to WT): K_{L834R} = 1.7 nM (K_{WT} = 3.4 nM); $k_{\mathrm{u-L834R}}^{\mathrm{DIM}}$=0.02 s^{-1} ($k_{\mathrm{u-WT}}^{\mathrm{DIM}}$=1.1 s^{-1}); $k_{\mathrm{KIN-L834R}}$ = 1.16 s^{-1} ($k_{\mathrm{KIN-WT}}$= 0.289 s^{-1}); $K45_{\mathrm{L834R}}$ = 10 nM ($K45_{\mathrm{WT}}$= 0.2 nM); in this way Cbl binding to the EGFR corresponds to ~20% at 20 ng/ml EGF, as experimentally shown[57].

REFERENCES TO SUPPLEMENTARY INFORMATION

1. Olsen JV*, et al.* Global, in vivo, and site-specific phosphorylation dynamics in signaling networks. *Cell* 127, 635-648 (2006).

2. Pryor MM, Low-Nam ST, Halasz AM, Lidke DS, Wilson BS, Edwards JS. Dynamic transition states of ErbB1 phosphorylation predicted by spatial stochastic modeling. *Biophysical journal* 105, 1533-1543 (2013).

3. Kleiman LB, Maiwald T, Conzelmann H, Lauffenburger DA, Sorger PK. Rapid phospho-turnover by receptor tyrosine kinases impacts downstream signaling and drug binding. *Mol Cell* 43, 723-737 (2011).

4. Monast CS, Furcht CM, Lazzara MJ. Computational analysis of the regulation of EGFR by protein tyrosine phosphatases. *Biophysical journal* 102, 2012-2021 (2012).

5. Kozer N*, et al.* Exploring higher-order EGFR oligomerisation and phosphorylation--a combined experimental and theoretical approach. *Mol Biosyst* 9, 1849-1863 (2013).

6. Kholodenko BN, Demin OV, Moehren G, Hoek JB. Quantification of short term signaling by the epidermal growth factor receptor. *J Biol Chem* 274, 30169-30181 (1999).

7. Lemmon MA, Ladbury JE, Mandiyan V, Zhou M, Schlessinger J. Independent binding of peptide ligands to the SH2 and SH3 domains of Grb2. *J Biol Chem* 269, 31653-31658 (1994).

8. Chook YM, Gish GD, Kay CM, Pai EF, Pawson T. The Grb2-mSos1 complex binds phosphopeptides with higher affinity than Grb2. *J Biol Chem* 271, 30472-30478 (1996).

9. Cussac D, Frech M, Chardin P. Binding of the Grb2 SH2 domain to phosphotyrosine motifs does not change the affinity of its SH3 domains for Sos proline-rich motifs. *EMBO J* 13, 4011-4021 (1994).

10. Hu J, Hubbard SR. Structural characterization of a novel Cbl phosphotyrosine recognition motif in the APS family of adapter proteins. *J Biol Chem* 280, 18943-18949 (2005).

11. Sastry L, Lin W, Wong WT, Di Fiore PP, Scoppa CA, King CR. Quantitative analysis of Grb2-Sos1 interaction: the N-terminal SH3 domain of Grb2 mediates affinity. *Oncogene* 11, 1107-1112 (1995).

12. Lemmon MA*, et al.* Two EGF molecules contribute additively to stabilization of the EGFR dimer. *EMBO J* 16, 281-294 (1997).

13. Houtman JC*, et al.* Oligomerization of signaling complexes by the multipoint binding of GRB2 to both LAT and SOS1. *Nat Struct Mol Biol* 13, 798-805 (2006).

14. Macdonald JL, Pike LJ. Heterogeneity in EGF-binding affinities arises from negative cooperativity in an aggregating system. *Proc Natl Acad Sci U S A* 105, 112-117 (2008).

15. French AR, Tadaki DK, Niyogi SK, Lauffenburger DA. Intracellular trafficking of epidermal growth factor family ligands is directly influenced by the pH sensitivity of the receptor/ligand interaction. *J Biol Chem* 270, 4334-4340 (1995).

16. Waters CM, Oberg KC, Carpenter G, Overholser KA. Rate constants for binding, dissociation, and internalization of EGF: effect of receptor occupancy and ligand concentration. *Biochemistry* 29, 3563-3569 (1990).

17. Klein P, Mattoon D, Lemmon MA, Schlessinger J. A structure-based model for ligand binding and dimerization of EGF receptors. *Proc Natl Acad Sci U S A* 101, 929-934 (2004).

18. Teramura Y, Ichinose J, Takagi H, Nishida K, Yanagida T, Sako Y. Single-molecule analysis of epidermal growth factor binding on the surface of living cells. *EMBO J* 25, 4215-4222 (2006).

19. Kusumi A, Sako Y, Yamamoto M. Confined lateral diffusion of membrane receptors as studied by single particle tracking (nanovid microscopy). Effects of calcium-induced differentiation in cultured epithelial cells. *Biophys J* 65, 2021-2040 (1993).

20. Low-Nam ST*, et al.* ErbB1 dimerization is promoted by domain co-confinement and stabilized by ligand binding. *Nat Struct Mol Biol* 18, 1244-1249 (2011).

21. Chung I, Akita R, Vandlen R, Toomre D, Schlessinger J, Mellman I. Spatial control of EGF receptor activation by reversible dimerization on living cells. *Nature* 464, 783-787 (2010).

22. Hucka M*, et al.* The systems biology markup language (SBML): a medium for representation and exchange of biochemical network models. *Bioinformatics* 19, 524-531 (2003).

23. Schmidt H, Jirstrand M. Systems Biology Toolbox for MATLAB: a computational platform for research in systems biology. *Bioinformatics* 22, 514-515 (2006).

24. Gunawardena J. Multisite protein phosphorylation makes a good threshold but can be a poor switch. *Proc Natl Acad Sci U S A* 102, 14617-14622 (2005).

25. Kinzer-Ursem TL, Linderman JJ. Both ligand- and cell-specific parameters control ligand agonism in a kinetic model of g protein-coupled receptor signaling. *PLoS Comput Biol* 3, e6 (2007).

26. Lu T, Shen T, Zong C, Hasty J, Wolynes PG. Statistics of cellular signal transduction as a race to the nucleus by multiple random walkers in compartment/phosphorylation space. *Proc Natl Acad Sci U S A* 103, 16752-16757 (2006).

27. Monod J, Wyman J, Changeux JP. On the Nature of Allosteric Transitions: A Plausible Model. *J Mol Biol* 12, 88-118 (1965).

28. Salazar C, Hofer T. Multisite protein phosphorylation--from molecular mechanisms to kinetic models. *FEBS J* 276, 3177-3198 (2009).

29. Gillespie DT. Exact stochastic simulation of coupled chemical reactions. *J Phys Chem* 81, 2340-2361 (1977).

30. Ferguson KM, Berger MB, Mendrola JM, Cho HS, Leahy DJ, Lemmon MA. EGF activates its receptor by removing interactions that autoinhibit ectodomain dimerization. *Mol Cell* 11, 507-517 (2003).

31. Raue A*, et al.* Structural and practical identifiability analysis of partially observed dynamical models by exploiting the profile likelihood. *Bioinformatics* 25, 1923-1929 (2009).

32. Venzon DJaM, S. H. A Method for Computing Profile-Likelihood-Based Confidence Intervals. *Journal of the Royal Statistical Society, Series C (Applied Statistics)* 37, 87-94 (1988).

33. Maiwald T, Timmer J. Dynamical modeling and multi-experiment fitting with PottersWheel. *Bioinformatics* 24, 2037-2043 (2008).

34. Alvarado D, Klein DE, Lemmon MA. Structural basis for negative cooperativity in growth factor binding to an EGF receptor. *Cell* 142, 568-579 (2010).

35. Arkhipov A*, et al.* Architecture and membrane interactions of the EGF receptor. *Cell* 152, 557-569 (2013).

36. Endres NF*, et al.* Conformational coupling across the plasma membrane in activation of the EGF receptor. *Cell* 152, 543-556 (2013).

37. Liu P, Cleveland TEt, Bouyain S, Byrne PO, Longo PA, Leahy DJ. A single ligand is sufficient to activate EGFR dimers. *Proc Natl Acad Sci U S A* 109, 10861-10866 (2012).

38. Dawson JP, Bu Z, Lemmon MA. Ligand-induced structural transitions in ErbB receptor extracellular domains. *Structure* 15, 942-954 (2007).

39. Ozcan F, Klein P, Lemmon MA, Lax I, Schlessinger J. On the nature of low- and high-affinity EGF receptors on living cells. *Proc Natl Acad Sci U S A* 103, 5735-5740 (2006).

40. Wofsy C, Goldstein B, Lund K, Wiley HS. Implications of epidermal growth factor (EGF) induced egf receptor aggregation. *Biophys J* 63, 98-110 (1992).

41. Macdonald-Obermann JL, Pike LJ. The intracellular juxtamembrane domain of the epidermal growth factor (EGF) receptor is responsible for the allosteric regulation of EGF binding. *J Biol Chem* 284, 13570-13576 (2009).

42. Lemmon MA, Schlessinger J, Ferguson KM. The EGFR family: not so prototypical receptor tyrosine kinases. *Cold Spring Harb Perspect Biol* 6, a020768 (2014).

43. Fan YX, Wong L, Deb TB, Johnson GR. Ligand regulates epidermal growth factor receptor kinase specificity: activation increases preference for GAB1 and SHC versus autophosphorylation sites. *J Biol Chem* 279, 38143-38150 (2004).

44. Yun CH*, et al.* Structures of lung cancer-derived EGFR mutants and inhibitor complexes: mechanism of activation and insights into differential inhibitor sensitivity. *Cancer Cell* 11, 217-227 (2007).

45. Zhang X, Gureasko J, Shen K, Cole PA, Kuriyan J. An allosteric mechanism for activation of the kinase domain of epidermal growth factor receptor. *Cell* 125, 1137-1149 (2006).

46. Guo L, Kozlosky CJ, Ericsson LH, Daniel TO, Cerretti DP, Johnson RS. Studies of ligand-induced site-specific phosphorylation of epidermal growth factor receptor. *J Am Soc Mass Spectrom* 14, 1022-1031 (2003).

47. Schmidt MH, Dikic I. The Cbl interactome and its functions. *Nat Rev Mol Cell Biol* 6, 907-918 (2005).

48. Vidal M, Liu WQ, Lenoir C, Salzmann J, Gresh N, Garbay C. Design of peptoid analogue dimers and measure of their affinity for Grb2 SH3 domains. *Biochemistry* 43, 7336-7344 (2004).

49. Yuzawa S*, et al.* Solution structure of Grb2 reveals extensive flexibility necessary for target recognition. *J Mol Biol* 306, 527-537 (2001).

50. Fukazawa T, Miyake S, Band V, Band H. Tyrosine phosphorylation of Cbl upon epidermal growth factor (EGF) stimulation and its association with EGF receptor and downstream signaling proteins. *J Biol Chem* 271, 14554-14559 (1996).

51. Meisner H, Czech MP. Coupling of the proto-oncogene product c-Cbl to the epidermal growth factor receptor. *J Biol Chem* 270, 25332-25335 (1995).

52. Lauffenburger DA, Linderman JJ. *Receptors. Models for binding, trafficking, and signaling*. Oxford University Press (1993).

53. Shoup D, Szabo A. Role of diffusion in ligand binding to macromolecules and cell-bound receptors. *Biophys J* 40, 33-39 (1982).

54. Lynch TJ*, et al.* Activating mutations in the epidermal growth factor receptor underlying responsiveness of non-small-cell lung cancer to gefitinib. *N Engl J Med* 350, 2129-2139 (2004).

55. Paez JG*, et al.* EGFR mutations in lung cancer: correlation with clinical response to gefitinib therapy. *Science* 304, 1497-1500 (2004).

56. Pao W*, et al.* EGF receptor gene mutations are common in lung cancers from "never smokers" and are associated with sensitivity of tumors to gefitinib and erlotinib. *Proc Natl Acad Sci U S A* 101, 13306-13311 (2004).

57. Shtiegman K*, et al.* Defective ubiquitinylation of EGFR mutants of lung cancer confers prolonged signaling. *Oncogene* 26, 6968-6978 (2007).

58. Red Brewer M, Yun CH, Lai D, Lemmon MA, Eck MJ, Pao W. Mechanism for activation of mutated epidermal growth factor receptors in lung cancer. *Proc Natl Acad Sci U S A* 110, E3595-3604 (2013).

59. Shan Y*, et al.* Oncogenic mutations counteract intrinsic disorder in the EGFR kinase and promote receptor dimerization. *Cell* 149, 860-870 (2012).
